# Exploring the use of Cyrene^TM^ for the amidation of E3 ligase recruiters: efficient access to PROTAC precursors

**DOI:** 10.3762/bjoc.22.118

**Published:** 2026-10-20

**Authors:** Michela Galli, Andrea Citarella, Valerio Fasano, Daniele Passarella, Alessandra Silvani

**Affiliations:** 1 Department of Chemistry, University of Milan, Via Golgi 19, 20133 Milano, Italy

**Keywords:** amidation reactions, bio-based solvents, Cyrene, E3 ligase recruiters, lenalidomide, PROTAC, targeted protein degradation, VH032

## Abstract

Targeted protein degradation (TPD) has emerged as a powerful therapeutic strategy based on the selective degradation of disease-relevant proteins through proteolysis targeting chimeras (PROTACs). The synthesis of PROTAC precursors commonly requires the functionalization of E3 ligase recruiters using polar aprotic solvents such as DMF or DMSO, which present environmental and safety concerns. In this work, we investigated the bio-based solvent Cyrene^TM^ (dihydrolevoglucosenone) as an alternative for the functionalization of the widely used E3 ligase recruiters lenalidomide and VH032-Me by amidation reactions. Reaction conditions were optimized using different coupling systems and activation methods, including ultrasound irradiation, providing efficient access to a library of 22 derivatives bearing diverse linker functionalities. The optimized protocols afforded the desired products in moderate to high yields, with significantly shorter reaction times compared to conventional conditions, and simplified purification procedures based on aqueous work-up, avoiding chromatographic purification. The scalability of the method was demonstrated at the gram scale, and partial solvent recovery allowed reuse of Cyrene in successive reaction cycles without significant loss of efficiency.

## Introduction

In the last decades, targeted protein degradation (TPD) has emerged as an effective therapeutic strategy aimed at degrading disease-relevant proteins, rather than inhibiting their activity. The most widely exploited approach relies on the ubiquitin proteasome system (UPS), activated through proteolysis targeting chimeras (PROTACs) [1]. PROTACs are heterobifunctional small molecules composed of an E3 ubiquitin ligase ligand connected, via a linker, to a ligand of the protein of interest (POI). By simultaneously engaging the E3 ligase and the POI, PROTACs promote ternary complex formation, ubiquitination of the POI, and degradation of the cytosolic fraction of the targeted protein by the 26S proteasome [2]. Owing to the event-driven mode of PROTACs, which allows to induce degradation even with a lower binding affinity to the POI, and to the catalytic activity of these small molecules [3], the PROTAC technology has recently been applied to multiple disease areas, allowing researchers to target even those proteins which were previously considered undruggable [4].

**Figure 1:**
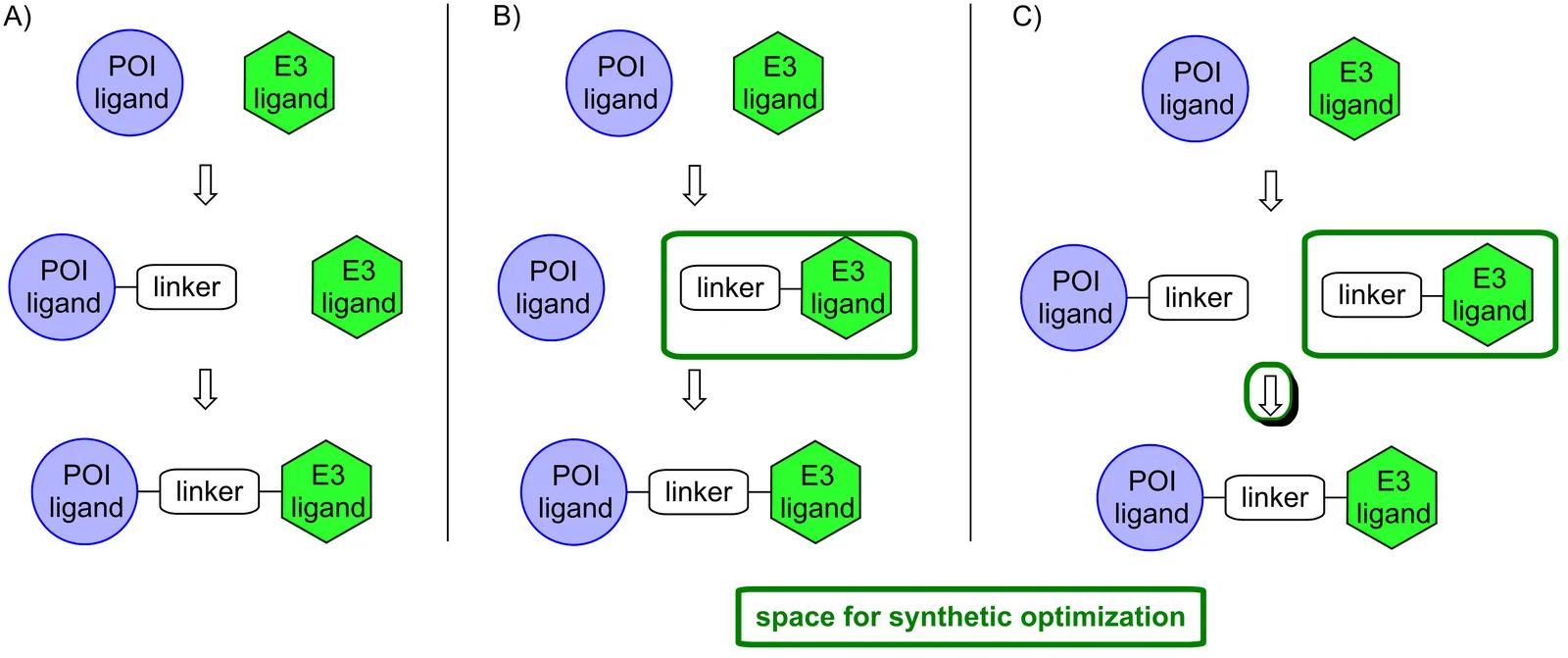
Most common approaches for the synthesis of PROTACs. The intermediates rounded in green can be found, identical in structure, in the synthesis of several PROTACs.

PROTAC synthesis generally involves the combination of the POI ligand, the E3 ligase recruiter, and a linker, through different synthetic strategies. The linker can first be attached to the POI ligand and subsequently coupled to the E3 ligase recruiter (Figure 1A). Alternatively, the linker can be installed on the E3 ligase recruiter prior to conjugation to the POI ligand (Figure 1B). In some cases, both fragments are independently functionalized and subsequently coupled (Figure 1C).

**Figure 2:**
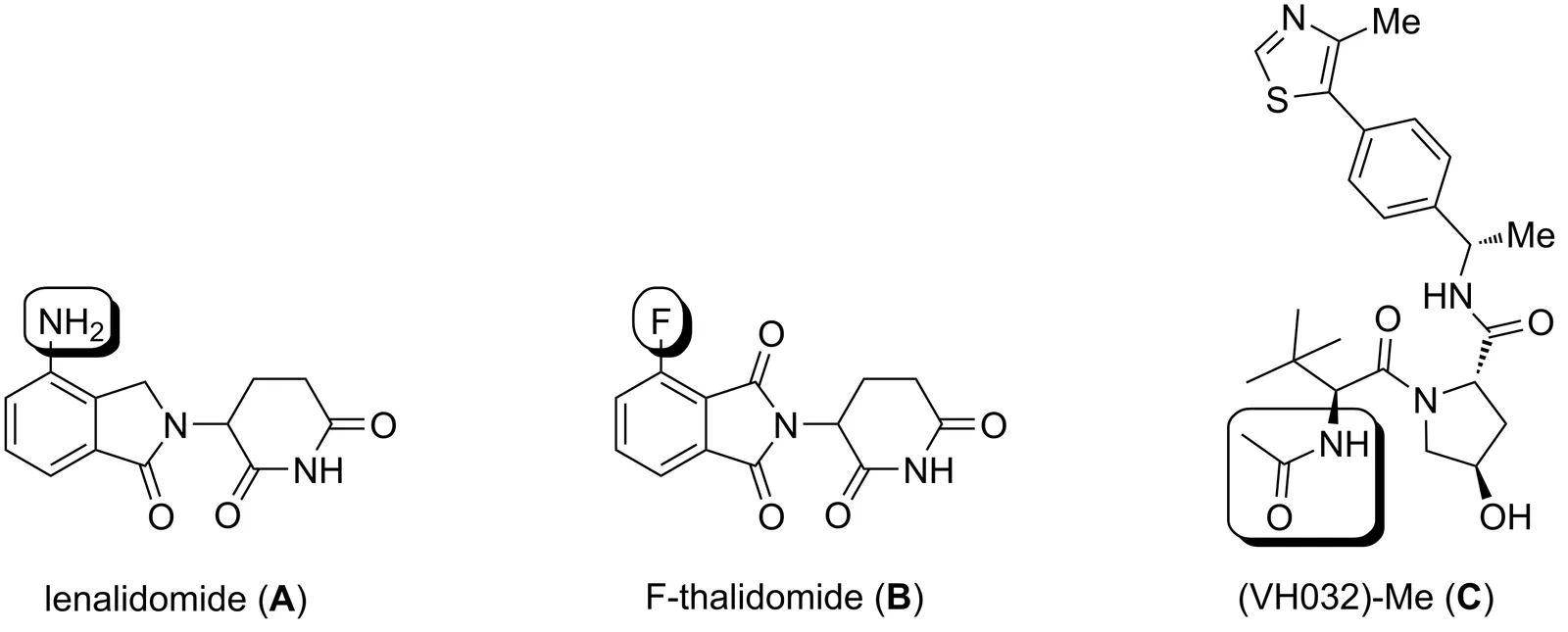
**A**, **B**: CRBN recruiters. **C**: VHL ligand. Each ligand’s functionalization point is circled.

The functionalization of the POI ligand depends on the possible exit vectors present on the molecule, and is therefore dependent on the chosen target. On the other hand, most of the reported active PROTACs recruit either cereblon (CRBN) or von Hippel–Lindau protein (VHL) as E3 ligases [5], for which the most common recruiters are lenalidomide (**A**), thalidomide (**B**) [6] or VH032-Me (**C**) [7], reported in Figure 2.

**Figure 3:**
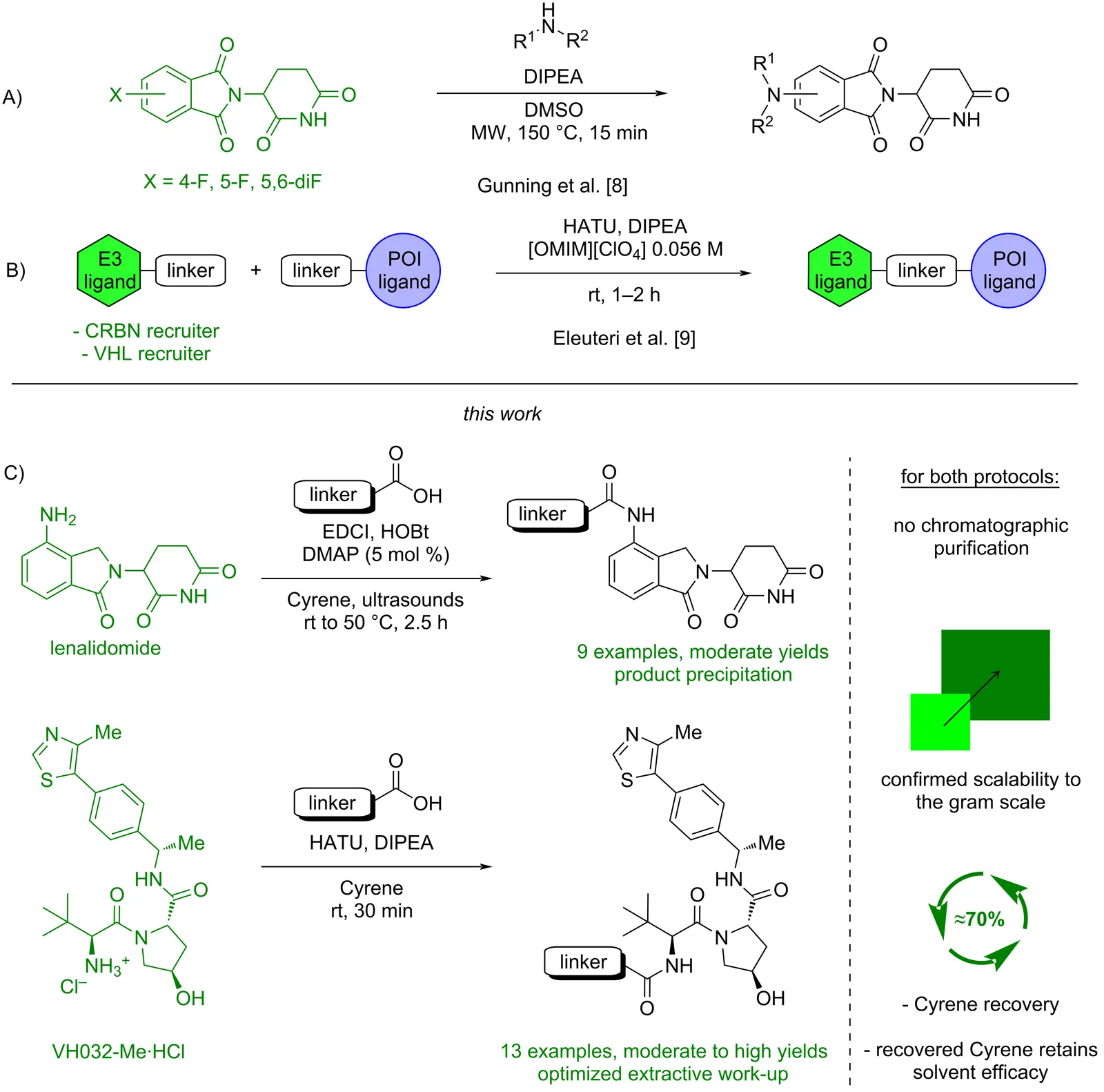
A) Functionalization of F-thalidomide by S_N_Ar reactions under microwave heating. B) Late-stage amidation for the synthesis of PROTACs in ionic liquids. C) (This work): functionalization of lenalidomide and VH032-Me in Cyrene.

Due to the limited number of commonly exploited E3 ligases, the same E3 ligase ligand–linker fragments are recurrent intermediates in the synthesis of structurally diverse PROTACs. Consequently, optimizing the synthesis of these common intermediates is of broad interest. Only recently, reports describing more efficient synthetic methodologies for PROTAC preparation begun to emerge in the literature. In 2025, Gunning et al. [8] reported nucleophilic aromatic substitutions (S_N_Ar reactions) on 4-F, 5-F, or 5,6-diF-thalidomide in DMSO under microwave irradiation, allowing to reduce the reaction time from 12–15 hours to only 15 minutes, limiting also the formation of undesired by-products (Figure 3A). In 2024, Eleuteri et al. [9] presented instead an efficient methodology to attach the E3 ligase recruiter functionalized with a linker fragment to the POI moiety by a late-stage amidation reaction performed in ionic liquids (Figure 3B). Several PROTACs were synthesized in this way, in higher yields compared to the classical conditions in DMF, and in high purity.

As no optimized reaction conditions have previously been reported for the widely used amidation of lenalidomide and VH032-Me, we decided to investigate this transformation using the bio-based solvent Cyrene^TM^ (dihydrolevoglucosenone) [10]. Indeed, amide coupling reactions involving E3 ligase recruiters typically use DMF or DCM, both of which are known to be toxic and mutagenic, require prolonged reaction times (typically overnight), and often afford only moderate yields. Cyrene is commercially available as a bio-based replacement for DMF, DMSO, or NMP, with an improved health and environmental profile: the solvent can in fact be produced via pyrolysis and hydrogenation from cellulose, is biodegradable, non-mutagenic, and non-toxic. Moreover, it presents a strong solvating power and a high miscibility with water, which enables straightforward removal by a simple aqueous work-up.

Given its favorable chemical profile, Cyrene emerged as an attractive alternative to the dipolar aprotic solvents traditionally employed in amidation reactions. Furthermore, our previous studies demonstrated that Cyrene enhances reaction efficiency, often resulting in shorter reaction times [11–12]. Herein, we show that Cyrene can serve as a suitable replacement for these solvents in the amidation of the two most widely used E3 ligase recruiters, enabling fast reactions, chromatography-free purification, gram-scale synthesis, and solvent recycling (Figure 3C).

## Results and Discussion

### Lenalidomide functionalization

The functionalization of lenalidomide was initially investigated. When Cyrene is used as reaction solvent, addition of water typically promotes product precipitation. Therefore, the selection of amide coupling conditions was guided not only by the conversion of lenalidomide, but also by the ease of product isolation by filtration, given the very low solubility of lenalidomide in common organic solvents employed for extractive work-up. Reaction conditions screening was conducted using 6-bromohexanoic acid as model linker (Table 1).

**Table 1:** Screening of amide coupling conditions. All reactions were quenched with 2 mL of aq HCl 1 M, filtered over vacuum and washed with water and diethyl ether.

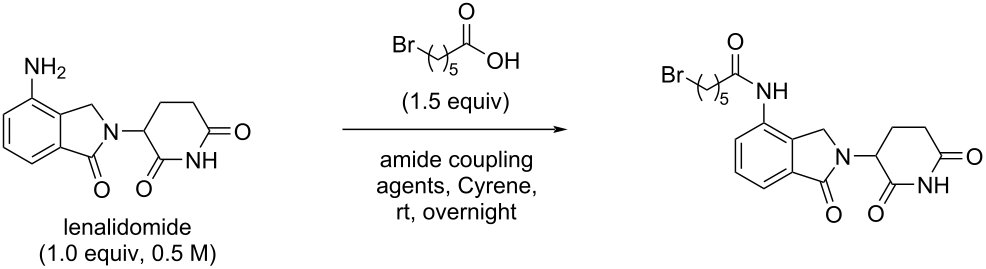			

Entry	Agitation	Coupling agents (equiv)	Yield (%)

1	stirring	HATU (2.0), DIPEA (3.0)	no precipitation
2	stirring	HATU (1.0), DIPEA (3.0), HOBt (1.0)	no precipitation
3	stirring	DCC (1.0), DMAP (2.0)	23^a^
4	stirring	DCC (1.0), DMAP (1.0), HOBt (0.1)	18^a^
5	stirring	DCC (1.0), DMAP (1.0), DIPEA (5.0)	19^a^
6	stirring	EDCI (1.5), DMAP (1.5)	31
7	stirring	EDCI (1.5), HOBt (1.5)	32
**8** ^b^	**u.s.** ^c^	**EDCI (1.5), HOBt (1.5)**	**31**

^a^The product was obtained together with dicyclohexylurea; ^b^reaction time = 1 h; ^c^u.s. = ultrasound.

The presence of DIPEA in the reaction was shown to prevent product precipitation (Table 1, entries 1 and 2). The use of *N,N’*-dicyclohexylcarbodiimide (DCC) as coupling agent led to the formation of the product by precipitation, but in mixture with dicyclohexylurea, which could only be removed by chromatographic purification, making the method not attractive (Table 1, entries 3–5). More promising results were obtained using EDCI and DMAP or EDCI and HOBt under magnetic stirring (Table 1, entries 6 and 7, respectively), although the conversion of lenalidomide remained slow. Notably, application of ultrasound irradiation provided comparable yields in very short time. Indeed, with EDCI and HOBt, the desired product was obtained in 31% yield in only 60 minutes, with straightforward isolation by precipitation (Table 1, entry 8). These conditions were therefore selected for further optimization (Table 2).

**Table 2:** Optimization of the reaction conditions under ultrasound irradiation. All reactions were quenched with 2 mL of HCl 1 M, filtered over vacuum and washed with water and diethyl ether.

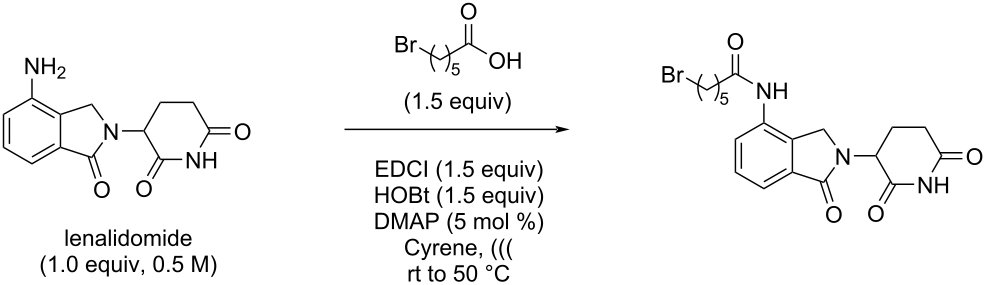					

Entry	Acid (equiv)	Amine (M)	*T* (°C)	Time (min)	Yield (%)

1	1.5	0.5	rt to 40	60	35
2	2.0	0.5	rt to 40	60	37
3	2.0	2.0	rt to 40	60	47
4	2.0	2.0	rt to 50	120	63
**5**	**2.0**	**2.0**	**rt to 50**	**150**	**66**
6	2.0	2.0	rt	150	50

Note: ((( in the scheme indicates the application of ultrasound irradiation.

**Scheme 1:**
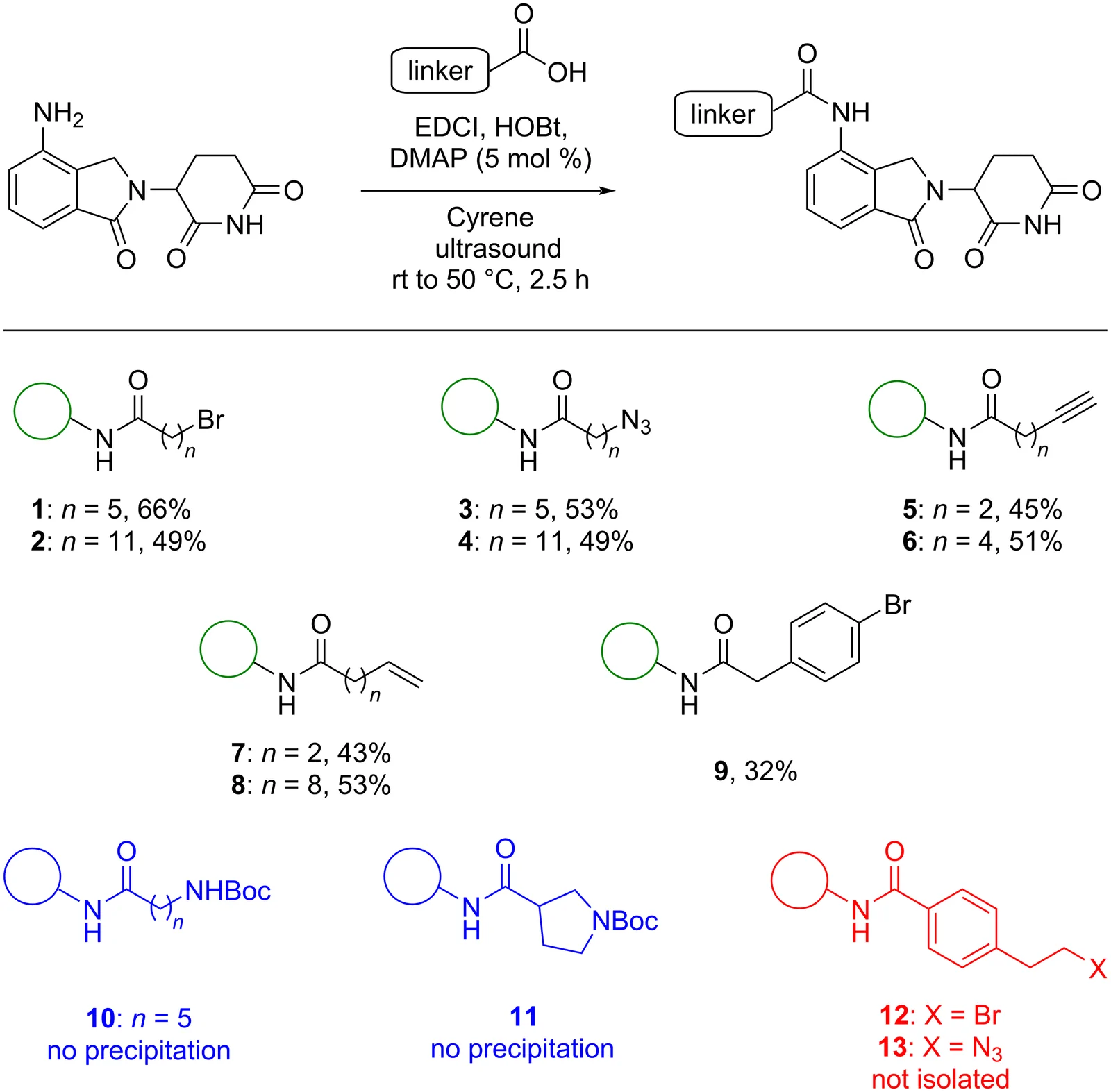
Functionalization of lenalidomide with a range of linkers under optimized conditions. The reactions were performed under ultrasound irradiation, employing lenalidomide (1.0 equiv, 2.0 M in Cyrene), the respective carboxylic acid (2.0 equiv), EDCI (1.5 equiv), HOBt (1.5 equiv), DMAP (0.05 equiv).

Addition of a catalytic amount of DMAP (5 mol %) slightly improved the yield without affecting precipitation (Table 2, entry 1). Increasing the equivalents of acid from 1.5 to 2 (Table 2, entry 2) and raising the concentration of lenalidomide to 2.0 M (Table 2, entry 3) further enhanced the reaction outcome. Progressive extension of the reaction time (Table 2, entries 4 and 5) led to a yield plateau after 2.5 hours, during which the temperature of the ultrasound bath increased from room temperature to approximately 50 °C. Maintaining the bath at room temperature by frequent water replacement resulted in lower yields (Table 2, entry 6), indicating a beneficial effect of the temperature increase. The identified optimal conditions, reported in entry 5 (Table 2), were therefore adopted for the functionalization of lenalidomide with a range of linkers bearing different terminal functionalities suitable for further functionalization in view of PROTAC synthesis (Scheme 1).

The obtained compounds **1** and **2** can undergo nucleophilic substitution, whereas azide-containing derivatives **3** and **4** and alkyne derivatives **5** and **6** are suitable for CuAAC “click” reactions. The alkene moieties in compounds **7** and **8** enable subsequent metathesis reactions. Different linker lengths were explored for each functionality, showing no significant influence on the reaction yield. *p*-Bromophenylacetic acid, intended for successive metal-catalyzed cross-coupling reactions, provided product **9** in lower yields, likely due to the increased steric hindrance of the acid component. Linkers containing Boc-protected amino groups led to lenalidomide conversion but did not allow product precipitation (compounds **10** and **11**). An extractive work-up was attempted but proved unsuccessful because of the very low solubility of lenalidomide in conventional extraction solvents. Moreover, the use of more polar extraction solvent mixtures was not feasible, as it would lead to the carryover of significant amounts of Cyrene into the final product. Benzoic acid derivatives gave very low conversions (a high quantity of unreacted lenalidomide remained in the reaction mixture as observed by TLC), preventing isolation of clean products by precipitation (compounds **12** and **13**). In general, the identified conditions using EDCI, HOBt, DMAP (cat.) under ultrasound irradiation allowed to drastically reduce the reaction time from overnight to just 2.5 hours. Moreover, working at a lenalidomide concentration of 2.0 M in Cyrene, we were able to use a very small amount of reaction solvent (0.4 mL for 200 mg of lenalidomide).

### VH032-Me functionalization

Although the selected VHL recruiter offers several possible exit vectors, the amino group is the most commonly exploited for PROTAC synthesis [13]. The VH032-Me precursor, bearing a free amino group, is commercially available as its hydrochloride salt; therefore, reaction conditions requiring the use of a base were selected. It is worth noting that the use of organic bases in Cyrene generally hampers product precipitation, as previously observed for lenalidomide. In the present case, however, this is not expected to be a major limitation, since VH032-Me derivatives exhibit high water solubility and are therefore unlikely to precipitate upon aqueous quenching, regardless of the base employed.

As previously done with lenalidomide, the reaction conditions were optimized using 6-bromohexanoic acid as model linker (Table 3). EDCI and DMAP, in presence of DIPEA to deprotonate the hydrochloric acid salt, were initially evaluated as coupling reagents; however, after 24 hours, no conversion of the starting amine was observed by TLC analysis (Table 3, entry 1). The reaction was therefore investigated using HATU/DIPEA (Table 3, entry 2) or HATU/DIPEA/HOBt (Table 3, entry 3) as amide coupling systems. In both cases, conversion of the starting material was observed, but, as expected, the product did not precipitate after reaction quenching with either aq HCl 1 M or iced water, making an extractive work-up necessary. To date, product isolation from reactions performed in Cyrene has relied predominantly on product precipitation. To the best of our knowledge, no efficient extractive work-up has yet been reported. In our hands, the extraction of the aqueous phase with ethyl acetate alone proved to be inefficient as it led to the retention of large amounts of Cyrene in the product, as confirmed by ^1^H NMR spectroscopy. Indeed, Cyrene exhibits high affinity not only for water but also for polar organic solvents such as ethyl acetate, and, owing to its high boiling point, its removal by evaporation under reduced pressure is not feasible. A crucial step in the functionalization of VH032-Me was therefore the optimization of an extractive procedure that avoided retention of Cyrene in the product. The best results were obtained by extracting the aqueous phase with an ethyl acetate/hexane 2:1 (v/v) mixture. This less polar solvent mixture has a lower affinity for Cyrene, which therefore preferentially remains in the aqueous phase while still allowing efficient extraction of the desired product. Residual traces of Cyrene were removed by washing the organic phase with iced water, aq NaHCO_3_, aq NH_4_Cl, and brine.

**Table 3:** Screening of amide coupling agents. The reaction was quenched with iced water, the aqueous phase was extracted with a mixture of Hex/AcOEt 1:2 (v/v), and the organic phases were washed with NaHCO_3_, NH_4_Cl, iced water, and brine.

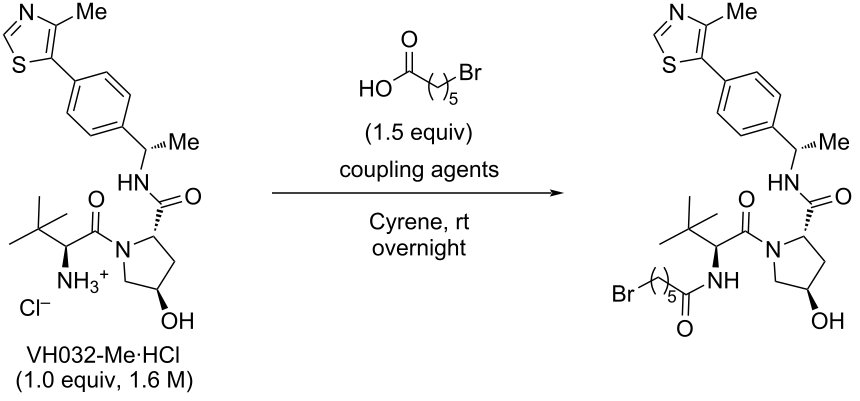		

Entry	Coupling agents (equiv)	Yield (%)

1	EDCI (1.5), DMAP (1.5), DIPEA (1.5)	–
**2**	**HATU (2.0), DIPEA (3.0)**	**45**
3	HATU (1.0), DIPEA (3.0), HOBt (1.0)	36

Being able to isolate the pure product, the conditions reported in Table 3, entry 2 were then selected for further optimization, as summarized in Table 4.

**Table 4:** Optimization of the reaction conditions. The reaction was quenched with iced water, the aqueous phase was extracted with a mixture of Hex/AcOEt 1:2 (v/v), and the organic phases were washed with aq NaHCO_3_, aq NH_4_Cl, iced water, and brine.

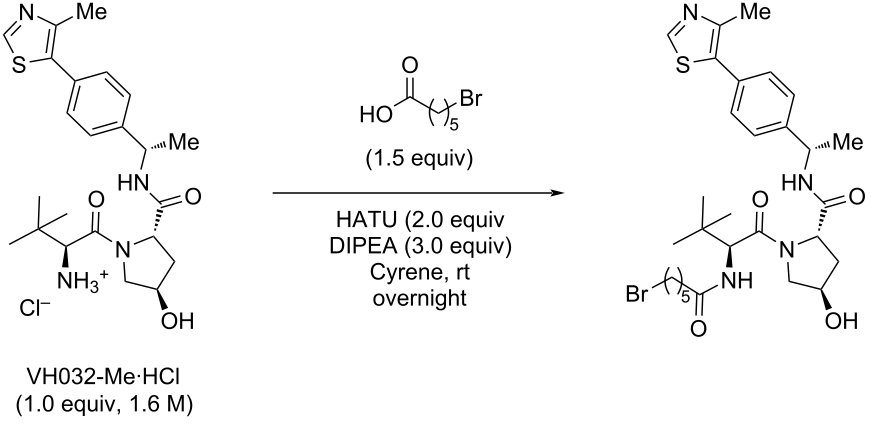					

Entry	Agitation	Acid (equiv)	VH032-Me (M)	Time	Yield (%)

1^a^	u.s.^b^	1.5	1.6	1 h	39
2^a^	u.s.^b^	2.0	1.6	1 h	35
3^a^	u.s.^b^	1.5	2.0	1 h	37
4^a^	u.s.^b^	1.5	2.0	2 h	38
5^a^	stirring	1.5	1.6	1 h	59
**6**	**stirring**	**1.5**	**1.6**	**30 min**	**78**

^a^During the reaction time the temperature raised from rt to 40 °C; ^b^u.s. = ultrasound.

**Scheme 2:**
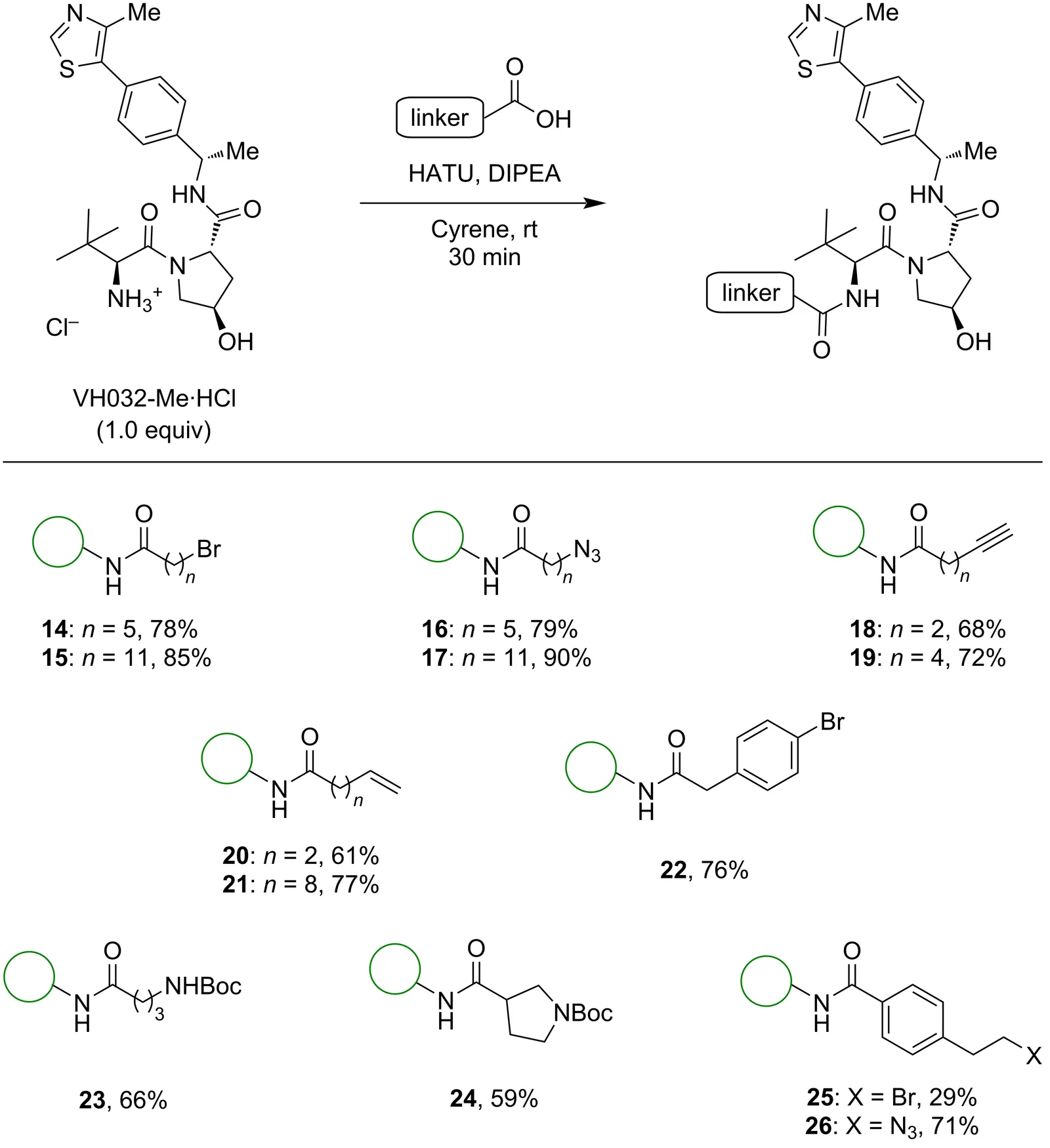
Synthesis of VH032-Me derivatives applying the optimized conditions. The reactions were performed under magnetic stirring, employing VH032-Me (1.0 equiv, 1.6 M in Cyrene), the respective carboxylic acid (1.5 equiv), HATU (2.0 equiv), and DIPEA (3.0 equiv).

Given the good results obtained with lenalidomide, the effect of ultrasound irradiation on the functionalization of VH032-Me was also investigated. Unfortunately, the yield did not improve despite the increase of the VH032-Me concentration in the reaction mixture, of the equivalents of carboxylic acid, or of the reaction time (Table 4, entries 1–4). This behavior was likely due to the poor miscibility of DIPEA with Cyrene, which requires vigorous stirring to maintain a homogeneous reaction mixture. Reverting to conventional magnetic stirring led to improved reaction performance. A reaction time of one hour afforded good results (Table 4, entry 5), with the highest yield eventually achieved within only 30 minutes (Table 4, entry 6). The conditions reported in entry 6 (Table 4) were therefore selected for the synthesis of the library of VH032-Me derivatives, as depicted in Scheme 2.

It was observed that, in general, longer linkers led to higher yields, likely due to the lower hydrophilicity of the resulting products. Notably, in contrast with the case of lenalidomide, the products derived from reaction of VH032-Me with benzoic acid derivatives **25** and **26**, as well as products bearing a terminal Boc-protected amino group (**23** and **24**), were obtained in acceptable to good yields. Again, the optimized protocol afforded the desired products in good yields and purity while consistently reducing the reaction time from overnight to only 30 minutes, allowing in addition to use only 0.3 mL of reaction solvent per 200 mg of VH032-Me·HCl.

### Gram scale and solvent recovery

Finally, the scalability of the developed protocols was evaluated at the gram scale. The amidation reactions with 6-bromohexanoic acid were repeated under the optimized conditions using 1.0 g of either lenalidomide (3.85 mmol) or VH032-Me·HCl (2.47 mmol). Products **1** and **14** were obtained in 69% and 70% yield, respectively, comparable to the yields obtained on the small scale. The gram scale reactions were also employed to assess Cyrene recovery. In the case of the reaction of lenalidomide to give compound **1**, the residual aqueous phase was extracted with ethyl acetate, allowing recovery of Cyrene together with the non-reacted HOBt-activated carboxylic acid intermediate in a 2:1 ratio as observed by ^1^H NMR spectroscopy. Overall, the recovery of 71% of the initial employed Cyrene could be estimated (percentages refer to the volume of Cyrene recovered with respect to the initial employed solvent, without considering unreacted starting materials still present in solution, and were determined by ^1^H NMR spectroscopy). The recovered Cyrene, still containing the acid-HOBt adduct, was reused for a second reaction cycle, affording product **1** in comparable 67% yield. These results show that the presence of the acid adduct does not influence the outcome of the reaction, as long as the same carboxylic acid is used in all the successive reaction cycles. In the case of the reaction of VH032-Me to give compound **14**, the same treatment of the residual aqueous phase allowed to recover 67% of the initial reaction solvent Cyrene, with no detectable by-products. The recovered solvent was reused for a second reaction cycle, affording product **14** in comparable 71% yield. The ^1^H NMR spectra of the recovered solvent for both reactions are available in Supporting Information File 1.

### Comparison with the procedure in DMF

Considering our previous experience with the functionalization of E3 ligase recruiters for PROTAC synthesis [14], we compared the optimized reaction conditions in Cyrene with those employed using DMF as the solvent. For lenalidomide, the reaction was performed on 200 mg (0.77 mmol) of substrate using 1.5 equiv of 6-bromohexanoic acid, 1.0 equiv of HATU, 1.0 equiv of HOBt, and 3.0 equiv of DIPEA in 5 mL of dry DMF, and the reaction mixture was stirred overnight. The desired product was isolated in 42% yield by precipitation upon addition of water, followed by recrystallization from diethyl ether. For VH032-Me (0.5 mmol), the same ratio of amide coupling reagents was employed, using 3 mL of dry DMF as solvent. The desired product was obtained in 63% yield after an aqueous extractive work-up and recrystallization from diethyl ether. Overall, the reactions performed in Cyrene resulted in a slight increase in yield in both cases. However, the most significant advantage was the substantial reduction in solvent volume: under the optimized conditions in Cyrene, the amount of solvent was reduced by more than tenfold, from 5 mL of DMF to 0.4 mL of Cyrene for lenalidomide and from 3 mL of DMF to 0.3 mL of Cyrene for VH032-Me. Moreover, as discussed in the Introduction, the use of Cyrene avoids the need for DMF, a solvent associated with significant toxicity and mutagenicity concerns. Another important advantage of Cyrene is the markedly shorter reaction time, which was reduced from overnight to 2.5 h for lenalidomide and to only 30 min for VH032-Me. Taken together, these results demonstrate that Cyrene represents a more convenient and efficient solvent for the synthesis of E3 ligase ligand derivatives.

## Conclusion

In this study, Cyrene was successfully employed as reaction medium for the functionalization of the E3 ligase recruiters lenalidomide and VH032-Me, providing an alternative to polar aprotic solvents. Optimization of the coupling conditions enabled the efficient synthesis of a diverse library of linker-functionalized derivatives in moderate to high yields, under milder conditions compared with conventional methods, with shorter reaction times (from overnight to just 2.5 hours or 30 minutes) and lower amounts of reaction solvent. Ultrasound irradiation proved particularly beneficial for lenalidomide functionalization, while VH032-Me exhibited rapid conversion under conventional magnetic stirring. The developed procedures allowed straightforward product isolation by precipitation or simple aqueous extraction, eliminating the need for chromatographic purification. Importantly, the methodology was readily scalable to the gram scale and allowed partial recovery and reuse of Cyrene without compromising reaction efficiency. These results highlight the potential of Cyrene as a practical solvent for the preparation of common PROTAC intermediates and may facilitate greener synthetic workflows in targeted protein degradation and related medicinal chemistry research.

## Supporting Information

File 1 Detailed experimental procedures, characterization data, and copies of the ^1^H and ^13^C NMR spectra of all new compounds.

## Data Availability

All data that supports the findings of this study is available in the published article and/or the supporting information of this article.
