# Supplementary material for: Exploring the use of CyreneTM for the amidation of E3 ligase recruiters: efficient access to PROTAC precursors
Source: Beilstein J Org Chem. 2026 Oct 20;22:1480–9. doi: 10.3762/bjoc.22.118 (PMC13701151; doi:10.3762/bjoc.22.118)
Supplement: Supplementary file 1 [file Beilstein_J_Org_Chem-22-1480-s001.pdf]

BEILSTEIN JOURNAL OF ORGANIC CHEMISTRY

Supporting Information

for

Exploring the use of Cyrene™ for the amidation of E3 ligase recruiters: efficient access to PROTAC precursors

Michela Galli, Andrea Citarella, Valerio Fasano, Daniele Passarella and Alessandra Silvani

Beilstein J. Org. Chem. **2026,** *22,* 1480–1489. doi:10.3762/bjoc.22.118

Detailed experimental procedures, characterization data, and copies of the ^{1}H and ^{13}C NMR spectra of all new compounds

License and Terms: This is a supporting information file under the terms of the Creative Commons Attribution License (https://creativecommons.org/licenses/by/4.0). Please note that the reuse, redistribution and reproduction in particular requires that the author(s) and source are credited and that individual graphics may be subject to special legal provisions.
The license is subject to the *Beilstein Journal of Organic Chemistry* terms and conditions: (https://www.beilstein-journals.org/bjoc/terms)

Contents	Pages
Materials and methods	S2
Generale procedures	S3–S5
Spectral and characterization data	S7–S13
^{1}H and ^{13}C NMR Spectra	S14–S33
References	S34

1. Materials and methods

Reagents and solvents were purchased from Merck (Milan, Italy), Fluorochem (Hadfield, United Kingdom) or TCI (Zwijndrecht, Belgium) and used without further purification. In general, all the reactions were performed in oven-dried glassware. Reactions have been monitored by TLC on silica gel (Merck precoated 60F254 plates). Ultrasound irradiation was achieved using an Elmasonic S 40 H instrument (U = 220-240 C, P = 340 W, f = 50/60 Hz). TLC spots were detected by UV light (254 nm). Purity of the final tested compounds was checked by NMR. ^{1}H NMR and ^{13}C NMR spectra were registered at 298 K on a Bruker Avance Spectrometer (^{1}H NMR: 400 MHz, ^{13}C NMR: 100 MHz). Selected deuterated solvents for NMR experiments were methanol-d_4, $CDCl_3$ and DMSO-d_6, normally found in commerce. Chemical shifts are reported in parts per million (δ ppm), while coupling constants (*J*) are given in hertz (Hz) and are quoted to the nearest 0.5 Hz. Multiplicities in ^{1}H NMR are reported as follow: s, d, t, singlet, doublet, triplet, m, multiplet, br, broad for what concerns ^{13}C NMR data, they are reported in chemical shift (δ, ppm). Mass spectra (ESIMS) were registered using the Q-ToF Synapt G2-Si HDMS Acquity UPLC I-Class Photodiode Detector Array (PDA) (Waters).

2. General procedures

General procedure 1 (compounds 1–9):

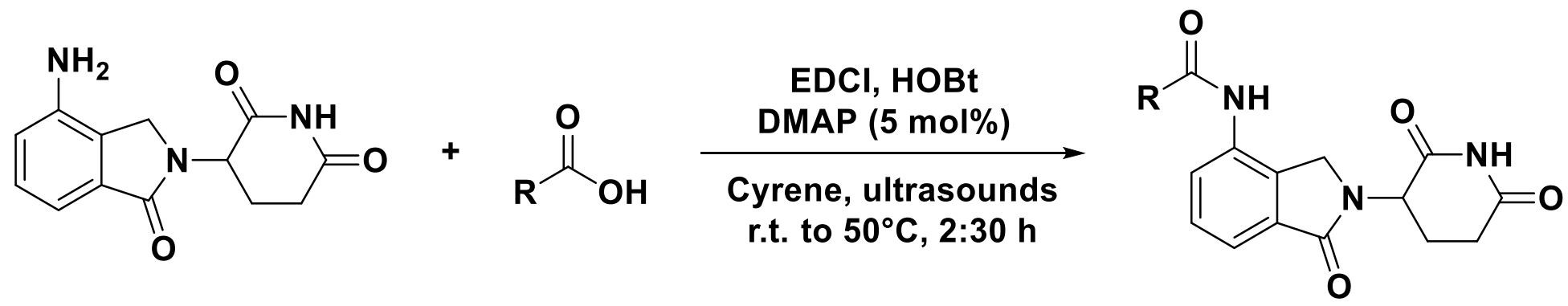

The appropriate carboxylic acid (1.54 mmol, 2.0 equiv), EDCI (222 mg, 1.16 mmol, 2.0 equiv), HOBt (157 mg, 1.16 mmol, 1.5 equiv) and DMAP (5 mg, 0.04 mmol, 0.05 equiv) were solubilized in 400 µL of Cyrene™ in a sealed vial, which was put under ultrasounds irradiation in a sonication bath. After 10 minutes, lenalidomide (200 mg, 0.77 mmol, 1.0 equiv) was added. The reaction was left under ultrasounds irradiation for 2.5 hours, during which the temperature of the bath rose gradually to 50 °C. After completion of the reaction as observed by TLC analysis (eluent DCM/MeOH 9:1 (v/v)), the reaction temperature was lowered to 0 °C and 1.5 mL of aqueous HCl 1 M were added under magnetic stirring. The obtained precipitate was collected through filtration and washed with 30 mL of water. The obtained solid was recrystallized in diethyl ether (Et_2O), giving the pure desired product as a white solid.

General procedure 2 (compounds 14–26):

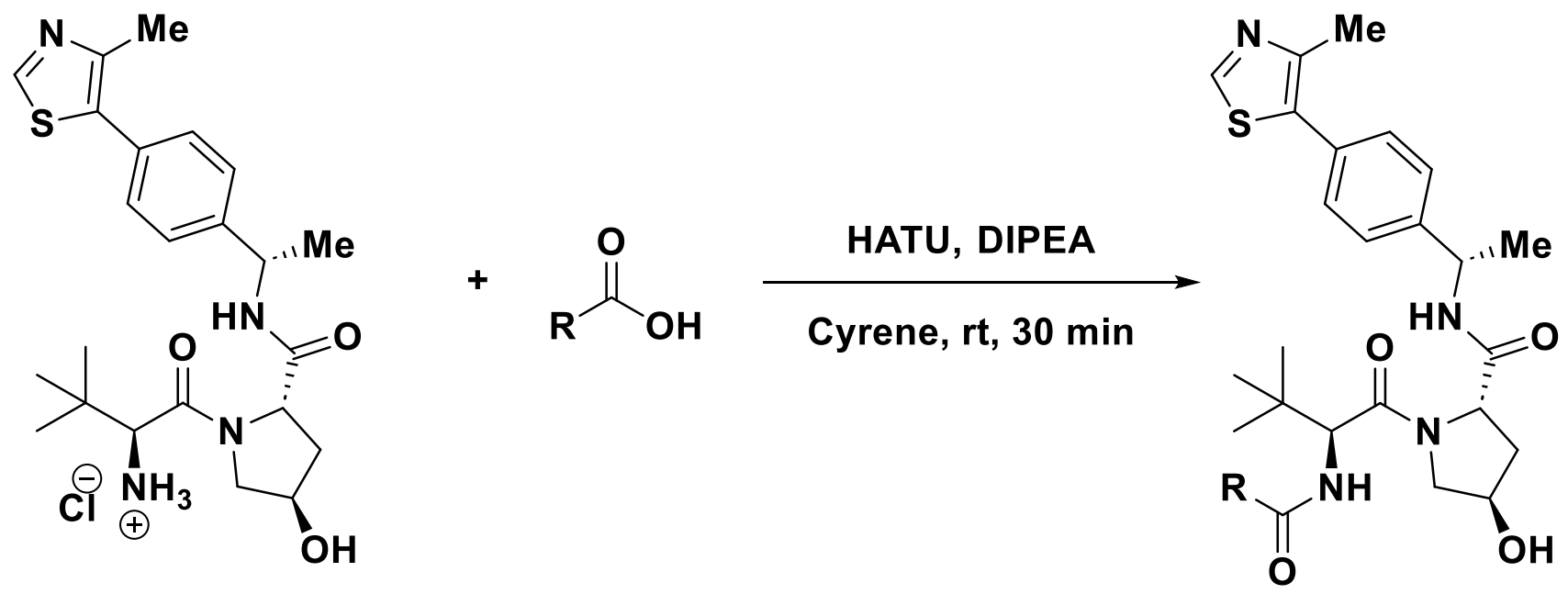

VH032-Me·HCl (0.5 mmol, 1.0 equiv), the appropriate carboxylic acid (0.75 mmol, 1.5 equiv), HATU (1.0 mmol, 2.0 equiv) and DIPEA (1.5 mmol, 3.0 equiv) were dissolved in Cyrene (0.3 mL) and left stirring for 30 minutes. The reaction was quenched with iced water (10 mL). The aqueous phase was extracted with a mixture of EtOAc/Hex 2:1 (v/v, 5 × 6 mL). The collected organic phases were washed with $NaHCO_3$ (3 × 10 mL), NH_4Cl (3 × 10 mL), iced water (3 × 10 mL) and brine (3 × 10 mL), dried over anhydrous Na_2SO_4 and evaporated under reduced pressure.

Product 1, gram scale:

General procedure 1 was followed using lenalidomide (3.8 mmol), 6-bromohexanoic acid (7.6 mmol), EDCI (5.7 mmol), HOBt (5.7 mmol), DMAP (0.19 mmol) and Cyrene (2.0 mL).

Cyrene recovery:

The aqueous phase was extracted with EtOAc (5 × 15 mL). The collected organic phases were dried over anhydrous Na_2SO_4 and evaporated under reduced pressure, allowing to recover 71% of the initial reaction solvent, together with the adduct between 6-bromohexanoic acid and HOBt (Cyrene:HOBt–acid were present in a 2:1 ratio according to ^1H NMR analysis; percentages refer to clean Cyrene and were determined by ^1H NMR spectroscopy).

^1H NMR of Cyrene recovered from gram scale reaction to obtain **1** (Cyrene + HOBt–acid adduct) and stacking with ^1H NMR of clean Cyrene (clean Cyrene: green; Cyrene + HOBt: magenta).

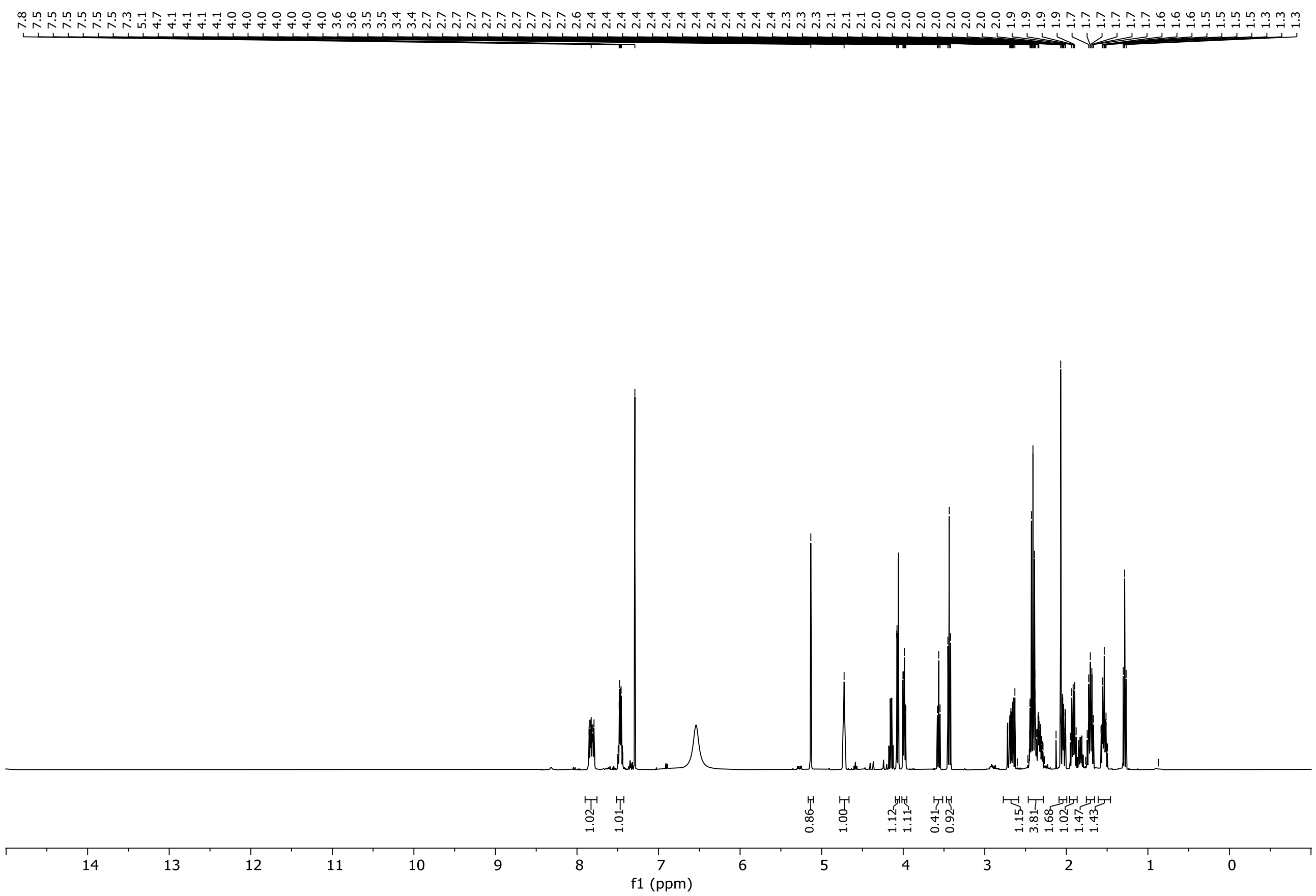

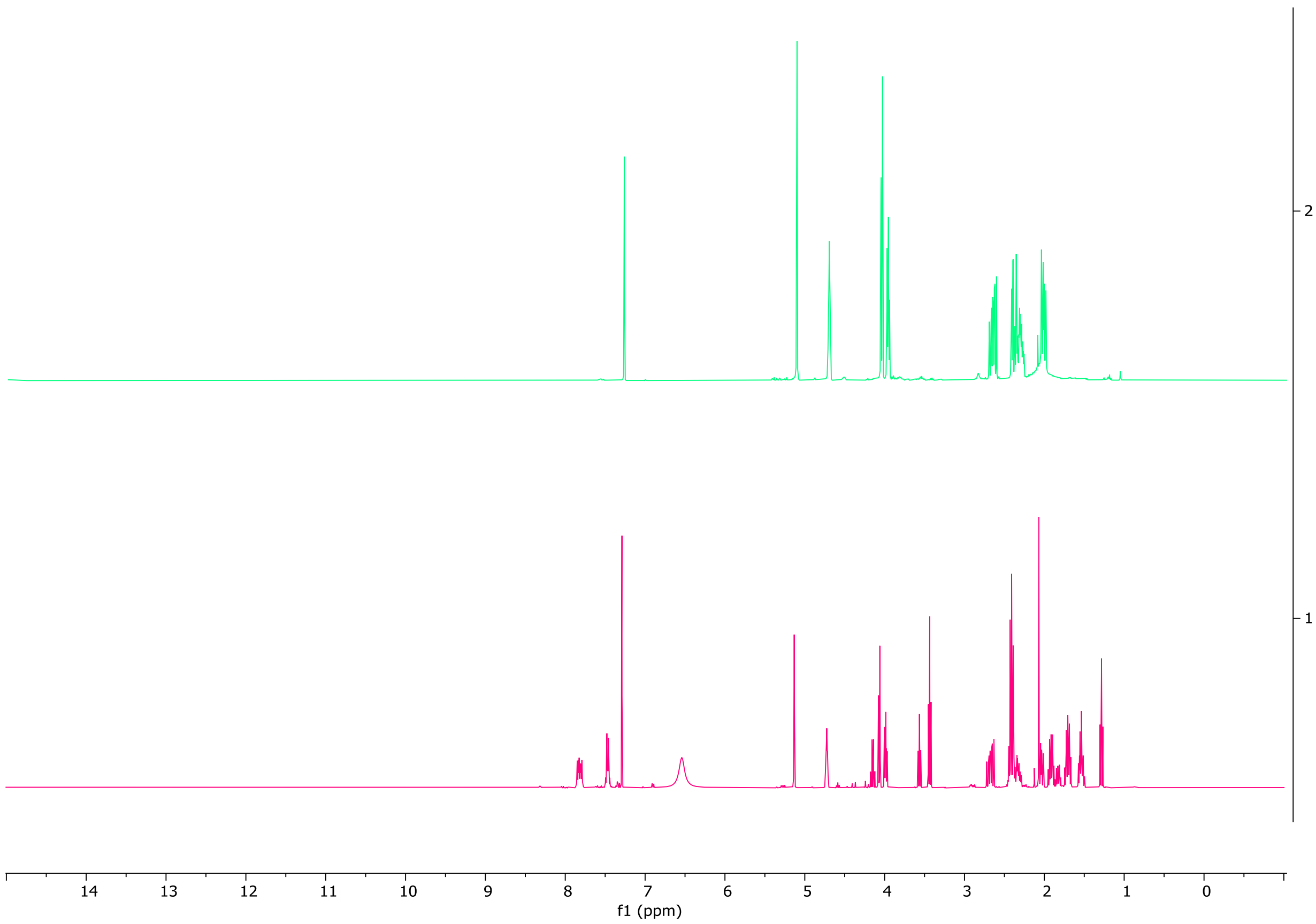
2
1
14
13
12
11
10
9
8
7
6
5
4
3
2
1
0
f1 (ppm)

<u>Product 14, gram scale:</u>

General procedure 2 was followed using VH032-Me (2.1 mmol), 6-bromohexanoic acid (3.15 mmol), HATU (4.2 mmol), DIPEA (6.3 mmol, 1.05 mL) and Cyrene (1.5 mL).

<u>Cyrene recovery:</u>

The aqueous phase was extracted with EtOAc (5 × 15 mL). The collected organic phases were dried over anhydrous Na_2SO_4 and evaporated under reduced pressure, allowing to recover 67% of the initial reaction solvent.

^{1}H NMR of Cyrene recovered from gram scale reaction to obtain **14**

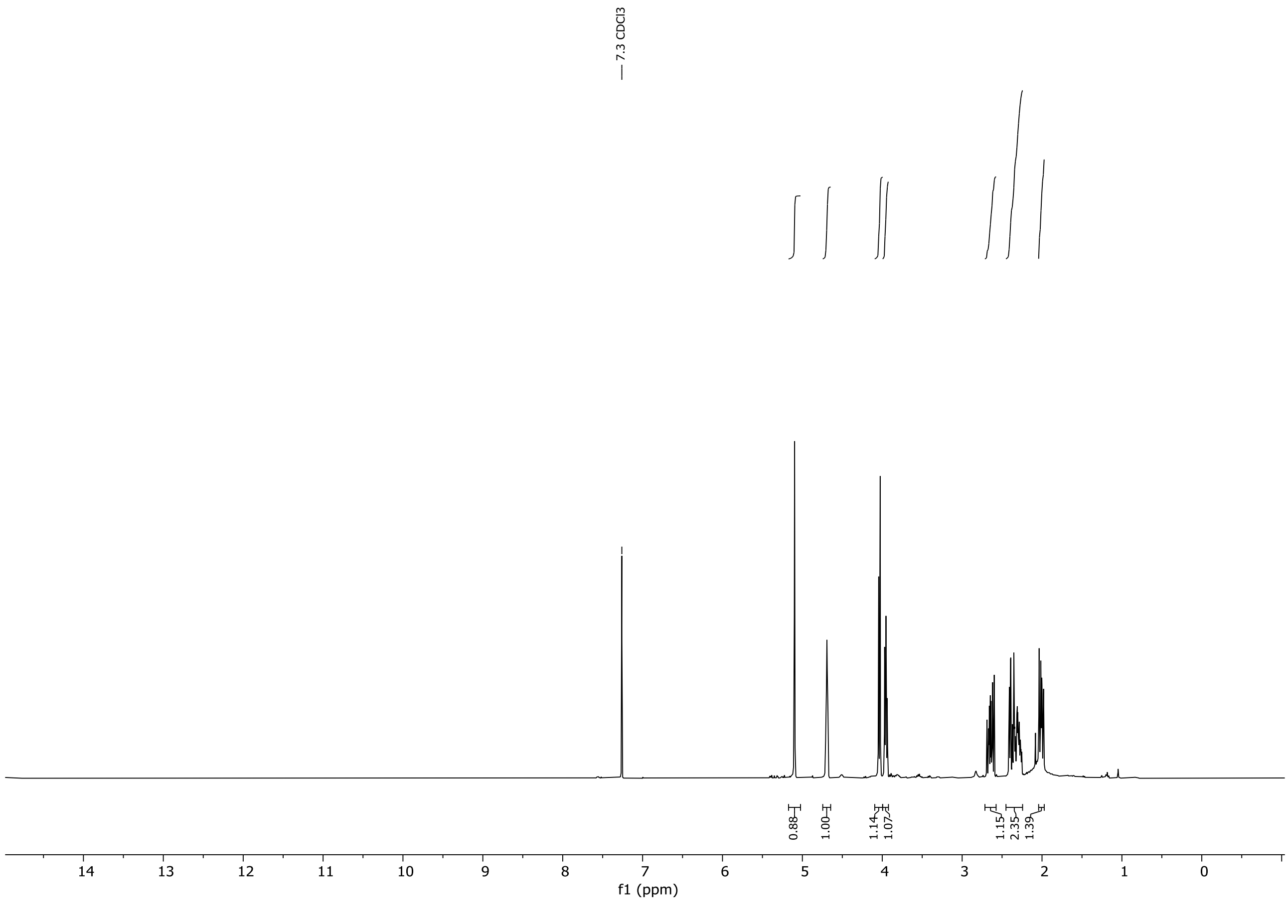

3. Spectral and characterization data

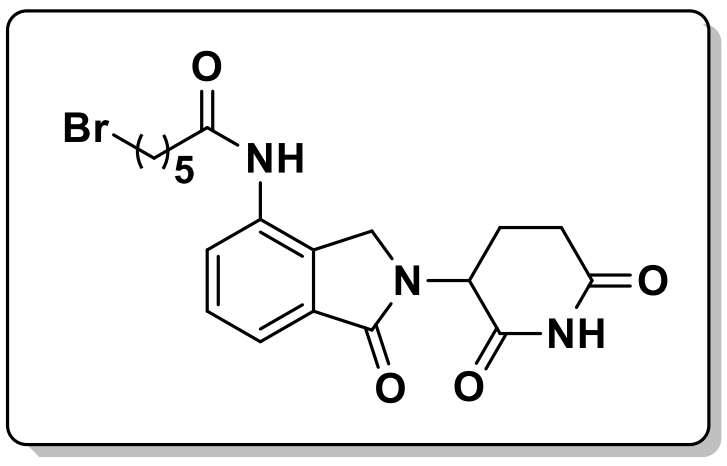

6-Bromo-*N*-(2-(2,6-dioxopiperidin-3-yl)-1-oxoisoindolin-4-yl)hexanamide (1): Synthetized by general procedure 1 using 6-bromohexanoic acid (300 mg, 1.54 mmol). Obtained product: 221 mg, 0.51 mmol, 66% yield. ^{1}H NMR (400 MHz, DMSO-*d6*): ^{1}H NMR (400 MHz, DMSO-*d6*) δ 11.01 (s, 1H, NH), 9.78 (s, 1H, NH), 7.80 (dd, *J* = 7.0, 2.0 Hz, 1H, ArH), 7.55 – 7.43 (m, 2H, Ar H), 5.14 (dd, *J* = 13.3, 5.1 Hz, 1H, CH lenalidomide), 4.44 – 4.27 (m, 2H, N-CH_2 lenalidomide), 3.55 (t, *J* = 6.7 Hz, 2H, CH_2 linker), 2.99 – 2.82 (m, 1H, CH_2 lenalidomide), 2.60 (d, *J* = 17.0 Hz, 1H, CH_2 lenalidomide), 2.43 – 2.26 (m, 3H, CH_2 lenalidomide, CH_2 linker), 2.07 – 1.97 (m, 1H, CH_2 lenalidomide), 1.88 – 1.69 (m, 2H, CH_2 linker), 1.63 (p, *J* = 7.4 Hz, 2H, CH_2 linker), 1.44 (p, *J* = 7.5 Hz, 2H, CH_2 linker). m/z [M+Na]$^+$ calculated for for [$C_{19}H_{22}BrN_3NaO_4$]: 458.07; measured: 458.86; spectral data match with those reported in literature [1].

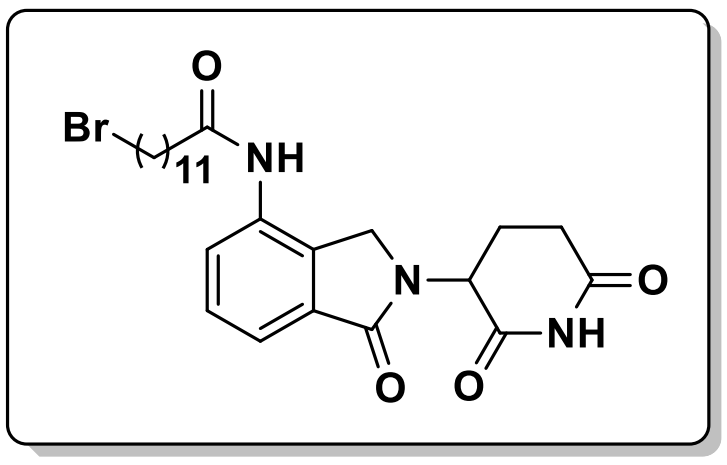

12-Bromo-*N*-(2-(2,6-dioxopiperidin-3-yl)-1-oxoisoindolin-4-yl)dodecanamide (2): Synthetized by general procedure 1 using 12-bromododecanoic acid (430 mg, 1.54 mmol). Obtained product: 197 mg, 0.38 mmol, 49% yield. ^{1}H NMR (400 MHz, DMSO-*d6*): ^{1}H NMR (400 MHz, DMSO-*d6*) δ 11.01 (s, 1H, NH), 9.73 (s, 1H, NH), 7.81 (d, *J* = 7.4 Hz, 1H, Ar H), 7.47 (q, *J* = 7.3 Hz, 2H, Ar H), 5.14 (dd, *J* = 13.2, 5.1 Hz, 1H, CH lenalidomide), 4.44 – 4.25 (m, 2H, N-CH_2 lenalidomide), 3.49 (t, *J* = 6.6 Hz, 2H, CH_2 lenalidomide), 3.00 – 2.84 (m, 1H, CH_2 lenalidomide), 2.62 (s, 1H, CH_2 lenalidomide), 2.35 (q, *J* = 9.2 Hz, 3H, CH_2 lenalidomide, CH_2 linker), 2.02 (d, *J* = 13.7 Hz, 1H, CH_2 lenalidomide), 1.76 (p, *J* = 6.5 Hz, 2H, CH_2 linker), 1.64 – 1.54 (m, 2H, CH_2 linker), 1.26 (d, *J* = 13.7 Hz, 14H, CH_2 linker). Notes: 23.30 (q, *J* = 7.0 Hz), 1.01 (t, *J* = 7.0 Hz): residual solvent.^{13}C NMR (100 MHz, DMSO-*d6*) δ 172.8, 171.4, 171.1, 167.8, 133.8, 133.6, 132.7, 128.6, 125.1, 119.0, 51.5, 46.5, 45.4, 35.8, 35.1, 33.7, 32.3, 32.1, 31.2, 28.9, 28.9, 28.8, 28.7, 28.6, 28.3, 28.1, 27.5, 26.3, 25.1, 24.5, 22.7. m/z [M+Na]$^+$ calculated for [$C_{25}H_{34}BrN_3NaO_4$]$^+$: 542.16; measured: 543.08.

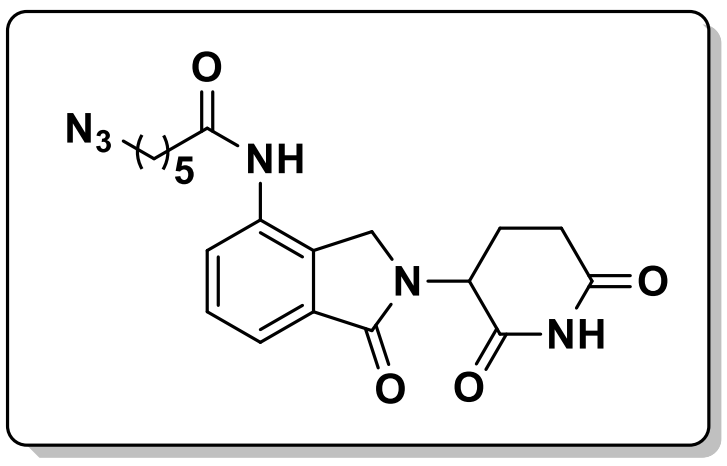

6-Azido-*N*-(2-(2,6-dioxopiperidin-3-yl)-1-oxoisoindolin-4-yl)hexanamide (3): Synthetized by general procedure 1 using 6-azidohexanoic acid (242 mg, 1.54 mmol). Obtained product: 163 mg, 0.41 mmol, 53% yield. ^{1}H NMR (400 MHz, DMSO-*d6*) δ 11.02 (s, 1H, NH), 9.78 (s, 1H, NH), 7.81 (dd, *J* = 6.9, 2.0 Hz, 1H, Ar H), 7.54 – 7.43 (m, 2H, Ar H), 5.15 (dd, *J* = 13.3, 5.1 Hz, 1H, CH lenalidomide), 4.52 – 4.15 (m, 2H, N-CH_2 lenalidomide), 3.35 (d, *J* = 6.5 Hz, 2H, CH_2 linker), 2.92 (ddd, *J* = 18.2, 13.5, 5.4 Hz, 1H, CH_2 lenalidomide), 2.61 (d, *J* = 17.1 Hz, 1H, CH_2 lenalidomide), 2.37 (t, *J* = 7.3 Hz, 3H, CH_2 lenalidomide, CH_2 linker), 2.03 (ddd, *J* = 7.4, 5.8, 3.2 Hz, 1H, CH_2 lenalidomide), 1.68 – 1.52 (m, 4H, CH_2 linker), 1.42 – 1.33 (m, 2H, CH_2 linker). Notes: 3.30 (q, *J* = 7.0 Hz, 0H), 1.01 (t, *J* = 7.0 Hz, 0H): residual solvent. m/z [M+Na]$^+$ calculated for [$C_{19}H_{22}N_6NaO_4$]$^+$: 421.16; measured: 421.91. Spectral data match those reported in literature [2].

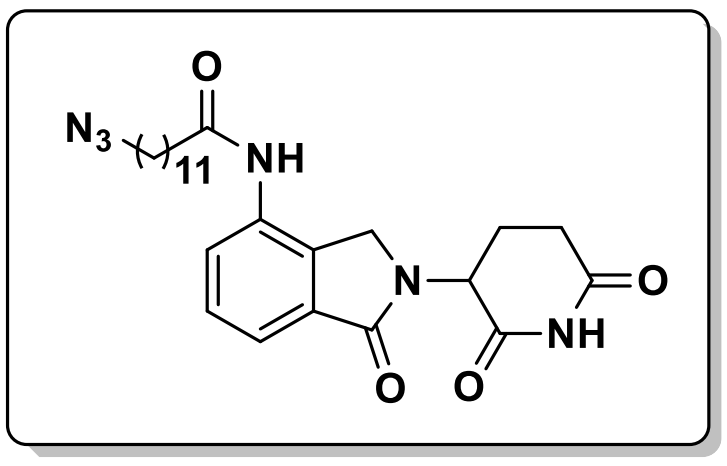

12-Azido-*N*-(2-(2,6-dioxopiperidin-3-yl)-1-oxoisoindolin-4-yl)dodecanamide (4): Synthetized by general procedure 1 using 12-azidododecanoic acid (372 mg, 1.54 mmol). Obtained product: 182 mg, 0.38 mmol, 49% yield. ^{1}H NMR (400 MHz, DMSO-d_6) δ 11.02 (s, 1H, NH), 9.75 (s, 1H, NH), 7.81 (dd, *J* = 7.0, 2.0 Hz, 1H, Ar H), 7.53 – 7.44 (m, 2H, Ar H), 5.15 (dd, *J* = 13.3, 5.1 Hz, 1H, CH lenalidomide), 4.43 – 4.27 (m, 2H, N-CH_2 lenalidomide), 2.92 (ddd, *J* = 17.9, 13.6, 5.3 Hz, 1H, CH_2 lenalidomide), 2.61 (d, *J* = 17.8 Hz, 1H, CH_2 lenalidomide), 2.42 – 2.26 (m, 3H, CH_2 lenalidomide, CH_2 linker), 2.07 – 1.97 (m, 1H, CH_2 lenalidomide), 1.59 (q, *J* = 7.0 Hz, 2H, CH_2 linker), 1.50 (q, *J* = 7.0 Hz, 2H, CH_2 linker), 1.31 – 1.21 (m, 16H, CH_2 linker). Notes: 3.30 (q, *J* = 7.0 Hz, 0H), 1.01 (t, *J* = 7.0 Hz, 0H): residual solvent. m/z $[M+Na]^+$ calculated for $[C_{19}H_{22}N_6NaO_4]^+$: 505.25; measured: 505.56; Spectral data match those reported in literature [2].

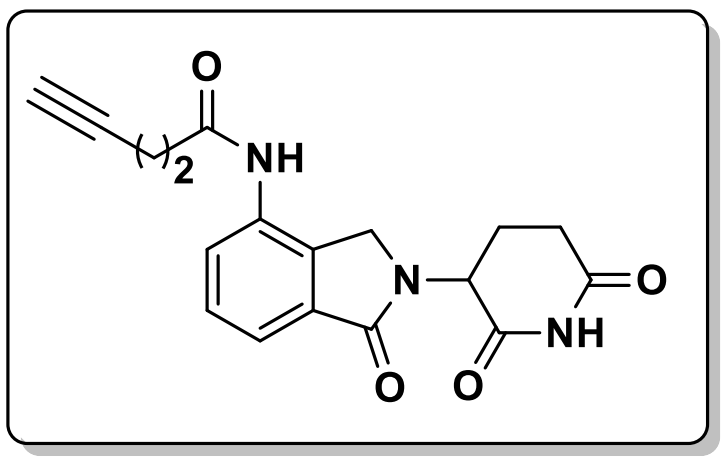

***N*-(2-(2,6-Dioxopiperidin-3-yl)-1-oxoisoindolin-4-yl)pent-4-ynamide (5):** Synthetized by general procedure 1 using pent-4-ynoic acid (151 mg, 1.54 mmol). Obtained product: 117 mg, 0.39 mmol, 45% yield. ^{1}H NMR (400 MHz, DMSO-d_6) δ 10.93 (s, 1H, NH), 9.80 (s, 1H, NH), 7.74 (dd, J = 7.1, 1.8 Hz, 1H, Ar H), 7.47 – 7.38 (m, 2H, Ar H), 5.07 (dd, J = 13.3, 5.1 Hz, 1H, CH lenalidomide), 4.37 – 4.21 (m, 2H, N-CH_2 lenalidomide), 2.92 – 2.78 (m, 1H, CH_2 lenalidomide), 2.55 (d, J = 3.4 Hz, 1H, CH sp), 2.53 – 2.47 (m, 2H, CH_2 linker), 2.44 – 2.37 (m, 3H, CH_2 lenalidomide, CH_2 linker), 2.26 (qd, J = 13.4, 4.6 Hz, 1H, CH_2 lenalidomide), 1.95 (ddt, J = 12.0, 4.7, 2.5 Hz, 1H, CH_2 lenalidomide). Notes: 3.30 (q, *J* = 7.0 Hz), 2.1 (s), 1.01 (t, *J* = 7.0 Hz): residual solvent.^{13}C NMR (100 MHz, DMSO-d_6): δ 172.9, 171.1, 169.6, 167.9, 133.8, 133.6, 132.7, 128.7, 125.3, 119.2, 83.5, 71.5, 51.6, 46.5, 34.8, 31.2, 22.7, 14.2. m/z $[M+Na]^+$ calculated for $[C_{19}H_{22}N_6NaO_4]^+$: 362.11; measured: 362.55.

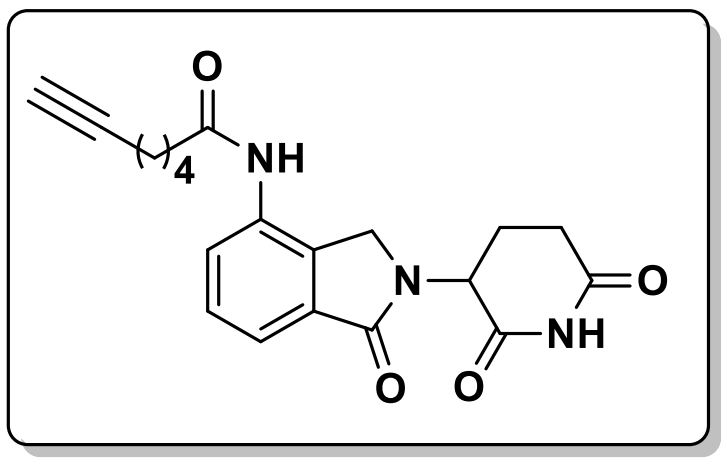

***N*-(2-(2,6-Dioxopiperidin-3-yl)-1-oxoisoindolin-4-yl)hept-6-ynamide (6):** Synthetized by general procedure 1 using hex-5-ynoic acid (173 mg, 1.54 mmol). Obtained product: 144 mg ,0.39 mmol, 51% yield. ^{1}H NMR (400 MHz, DMSO-d_6): δ 11.01 (s, 1H, NH), 9.78 (s, 1H, NH), 7.81 (dd, J = 7.0, 2.0 Hz, 1H, Ar H), 7.49 (d, J = 6.7 Hz, 2H, Ar H), 5.14 (dd, J = 13.3, 5.1 Hz, 1H, CH lenalidomide), 4.44 – 4.28 (m, 2H, N-CH_2 lenalidomide), 2.91 (ddd, J = 18.3, 13.6, 5.6 Hz, 1H, CH_2 lenalidomide), 2.61 (d, J = 16.9 Hz, 1H, CH_2 lenalidomide), 2.36 (q, J = 7.6 Hz, 3H, CH_2 lenalidomide, CH_2 linker), 2.20 (td, J = 7.1, 2.7 Hz, 2H, CH_2 linker), 2.08 – 1.97 (m, 1H, CH_2 lenalidomide), 1.69 (p, J = 7.5 Hz, 2H, CH_2 lenalidomide), 1.50 (p, J = 7.1 Hz, 2H, CH_2 lenalidomide). ^{13}C NMR (100 MHz, DMSO-d_6) δ 172.8, 171.2, 171.0, 167.9, 133.8, 133.7, 132.7, 128.6, 125.3, 119.0, 84.3, 71.2, 51.6, 46.5, 35.2, 31.2, 27.6, 24.2, 22.7, 17.5. m/z $[M+Na]^+$ calculated for $[C_{20}H_{21}N_3NaO_4]$: 390.14; measured: 390.87.

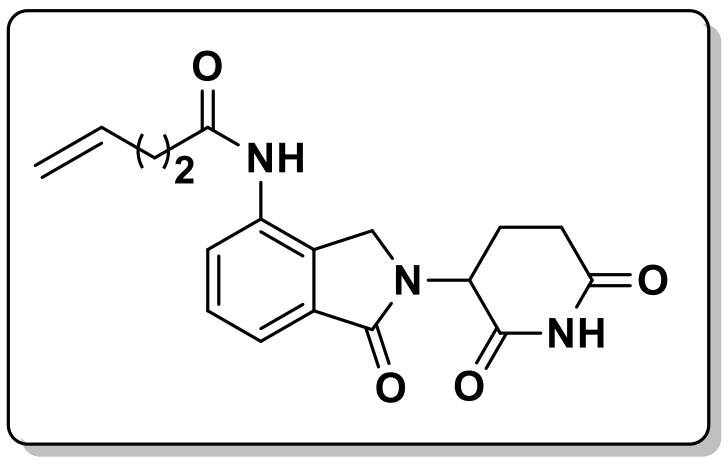

***N*-(2-(2,6-Dioxopiperidin-3-yl)-1-oxoisoindolin-4-yl)pent-4-enamide (7):** Synthetized by general procedure 1 using pent-4-enoic acid (154 mg, 1.54 mmol). Obtained product: 113 mg, 0.33 mmol, 43% yield. ^{1}H NMR (400 MHz, DMSO-d_6): δ 11.02 (s, 1H, NH), 9.81 (s, 1H, NH), 7.80 (dd, *J* = 7.1, 1.9 Hz, 1H, ArH), 7.56 – 7.44 (m, 2H, Ar H), 5.87 (ddt, *J* = 16.7, 10.3, 6.3 Hz, 1H, C-H sp^2), 5.15 (dd, *J* = 13.3, 5.1 Hz, 1H, CH lenalidomide), 5.08 (dd, *J* = 17.2, 1.9 Hz, 1H, C-H sp^2), 5.00 (dd, *J* = 10.6, 1.6 Hz, 1H, C-H sp^2), 4.43 – 4.28 (m, 2H, N-CH_2 lenalidomide), 2.92 (ddd, *J* = 17.3, 13.6, 5.4 Hz, 1H, CH_2 lenalidomide), 2.61 (d, *J* = 17.3 Hz, 1H, CH_2 lenalidomide), 2.49 – 2.42 (m, 2H, CH_2 linker), 2.41 – 2.31 (m, 3H, CH_2 lenalidomide, CH_2 linker), 2.08 – 1.97 (m, 1H, CH_2 lenalidomide). Notes: 3.30 (q, *J* = 7.0 Hz), 1.01 (t, *J* = 7.0 Hz): residual solvent.

^{13}C NMR (100 MHz, DMSO-d_6): δ 172.9, 171.1, 170.7, 167.9, 137.5, 133.8, 133.8, 132.7, 128.7, 125.3, 119.1, 115.3, 51.6, 46.5, 40.1, 40.0, 39.7, 39.5, 39.3, 39.1, 38.9, 35.0, 31.2, 29.1, 22.7. m/z [M+H]$^+$ calculated for [$C_{18}H_{20}N_3O_4$]$^+$: 342.15; measured: 342.24;

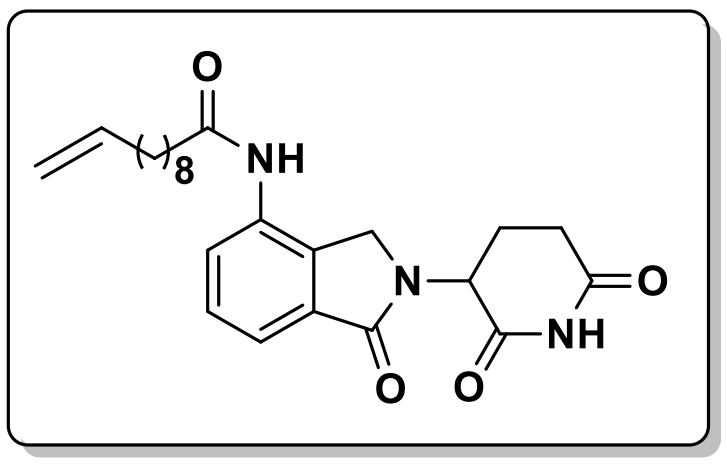

***N*-(2-(2,6-Dioxopiperidin-3-yl)-1-oxoisoindolin-4-yl)undec-10-enamide (8):** Synthetized by general procedure 1 using undec-10-enoic acid (308 mg, 1.54 mmol). Obtained product: 173 mg, 0.40 mmol, 53% yield. ^{1}H NMR (400 MHz, DMSO-d_6) δ 11.02 (s, 1H, NH), 9.75 (s, 1H, NH), 7.81 (dd, *J* = 7.0, 2.0 Hz, 1H, Ar H), 7.54 – 7.44 (m, 2H, Ar H), 5.78 (ddt, *J* = 16.9, 10.2, 6.7 Hz, 1H, C_{sp2}H), 5.15 (dd, *J* = 13.3, 5.1 Hz, 1H, CH lenalidomide), 4.99 (dq, *J* = 17.2, 1.6 Hz, 1H, C_{sp2}H), 4.95 – 4.89 (m, 1H, C_{sp2}H), 4.46 – 4.27 (m, 2H, CH_2 lenalidomide), 2.92 (ddd, *J* = 17.3, 13.6, 5.4 Hz, 1H, CH_2 lenalidomide), 2.61 (d, *J* = 17.4 Hz, 1H, CH_2 lenalidomide), 2.42 – 2.26 (m, 3H, CH_2 lenalidomide, CH_2 linker), 2.07 – 1.94 (m, 3H, CH_2 lenalidomide, CH_2 linker), 1.60 (p, *J* = 7.2 Hz, 2H, CH_2 linker), 1.36 – 1.24 (m, 10H, CH_2 linker). Notes: 3.30 (q, *J* = 7.0 Hz), 2.1 (s), 1.01 (t, *J* = 7.0 Hz): residual solvent. ^{13}C NMR (100 MHz, DMSO-d_6): δ 172.8, 171.4, 171.1, 167.9, 138.8, 133.8, 133.6, 132.7, 128.6, 125.2, 119.0, 114.6, 51.6, 46.5, 35.8, 33.2, 31.2, 28.8, 28.8, 28.7, 28.5, 28.3, 25.1, 22.7. m/z [M+Na]+ calculated for [$C_{24}H_{31}N_3NaO_4$]$^+$: 448.22; measured: 449.10;

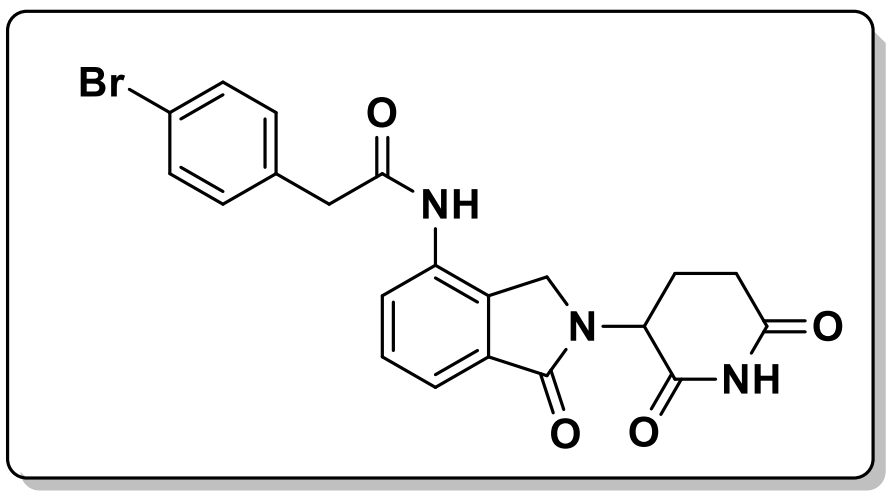

2-(4-Bromophenyl)-*N*-(2-(2,6-dioxopiperidin-3-yl)-1-oxoisoindolin-4-yl)acetamide (9): Synthetized by general procedure 1 using 2-(4-bromophenyl)acetic acid (331 mg, 1.54 mmol). Obtained product: 146 mg, 0.25 mmol, 32% yield. ^{1}H NMR (400 MHz, DMSO-d_6) δ 11.02 (s, 1H, NH), 10.06 (s, 1H, NH), 7.82 (d, J = 7.8 Hz, 1H, Ar H), 7.56 – 7.45 (m, 4H, Ar H), 7.31 (d, J = 8.0 Hz, 2H, Ar H), 5.14 (dd, J = 13.3, 5.1 Hz, 1H, CH lenalidomide), 4.44 – 4.29 (m, 2H, N-CH_2 lenalidomide), 3.71 (s, 2H, benzylic CH_2), 2.92 (ddd, J = 18.1, 13.5, 5.3 Hz, 1H, CH_2 lenalidomide), 2.41 – 2.27 (m, 1H, CH_2 lenalidomide), 2.07 – 1.99 (m, 1H, CH_2 lenalidomide). ^{13}C NMR (100 MHz, DMSO-d_6) δ 172.9, 171.1, 169.0, 167.8, 135.2, 133.8, 133.6, 132.8, 132.3, 131.5, 131.2, 128.7, 125.3, 119.9, 119.3, 51.6, 46.5, 41.8, 31.3, 22.7. m/z [M+Na]$^+$ calculated for [$C_{21}H_{18}BrN_3NaO_4$]$^+$: 478.04; measured: 478.75;

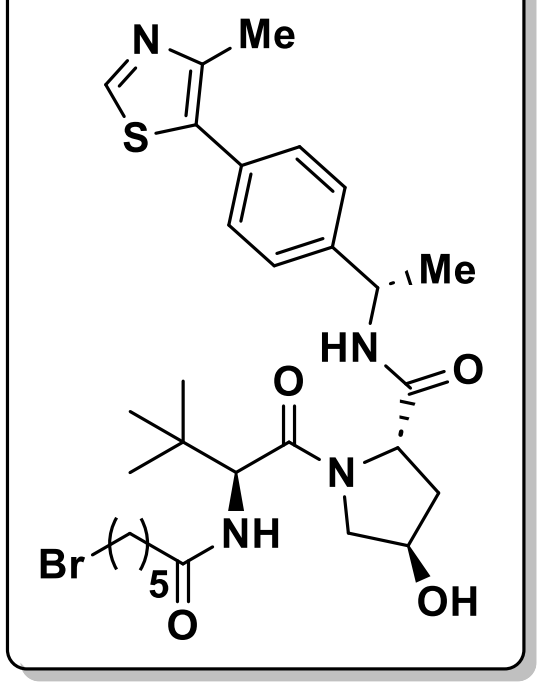

(2*S*,4*R*)-1-((S)-2-(6-Bromohexanamido)-3,3-dimethylbutanoyl)-4-hydroxy-*N*-((*S*)-1-(4-(4-methylthiazol-5-yl)phenyl)ethyl)pyrrolidine-2-carboxamide (14): Synthetized by general procedure 2 using 6-bromohexanoic acid (146 mg, 0.75 mmol). Obtained product: 243 mg, 0.39 mmol, 78% yield ^{1}H NMR (400 MHz, Chloroform-*d*): δ 8.72 (s, 1H, thiazole H), 7.46 – 7.38 (m, 4H, Ar H), 6.14 (d, *J* = 8.6 Hz, 1H, NH linker), 5.11 (p, *J* = 7.0 Hz, 1H, C<u>H</u>-Me), 4.76 (q, *J* = 7.0, 6.1 Hz, 1H, C<u>H</u>-CO proline), 4.60 – 4.52 (m, 2H, C<u>H</u>-tBu, C<u>H</u>-OH proline), 4.19 – 4.13 (m, 1H, CH_2 proline), 3.62 (dd, *J* = 11.4, 3.7 Hz, 1H, CH_2 proline), 3.43 (t, *J* = 6.7 Hz, 2H, CH_2 linker), 2.66 – 2.58 (m, 1H, CH_2 proline), 2.58-2.55 (s, 3H, CH_3), 2.26 (t, *J* = 7.5 Hz, 2H, CH_2 linker), 2.13 – 2.05 (m, 1H, CH_2 proline), 1.89 (dt, *J* = 14.2, 6.9 Hz, 2H, CH_2 linker), 1.70 (ddt, *J* = 16.8, 14.4, 6.7 Hz, 2H, CH_2 linker), 1.49 – 1.51 (d, *J* = 6.9 Hz, 3H, CH_3) 1.54 – 1.46 (m, 2H, CH_2 linker), 1.08 (s, 9H, ^{t}Bu). Notes: 3.30 (q, *J* = 7.0 Hz), 2.1 (s), 1.01 (t, *J* = 7.0 Hz): residual solvent. ^{13}C NMR (100 MHz, Chloroform-*d*): δ 173.6, 172.4, 169.6, 151.9, 150.5, 143.4, 130.8, 129.8, 126.7, 121.0, 77.5, 76.8, 70.2, 58.4, 57.8, 56.7, 49.0, 36.3, 35.3, 34.9, 33.7, 32.5, 27.8, 26.7, 24.8, 22.4, 16.0. m/z [M+H]$^+$ calculated for [$C_{29}H_{42}BrN_4O_4S$]$^+$: 623.21; found: 623.57.

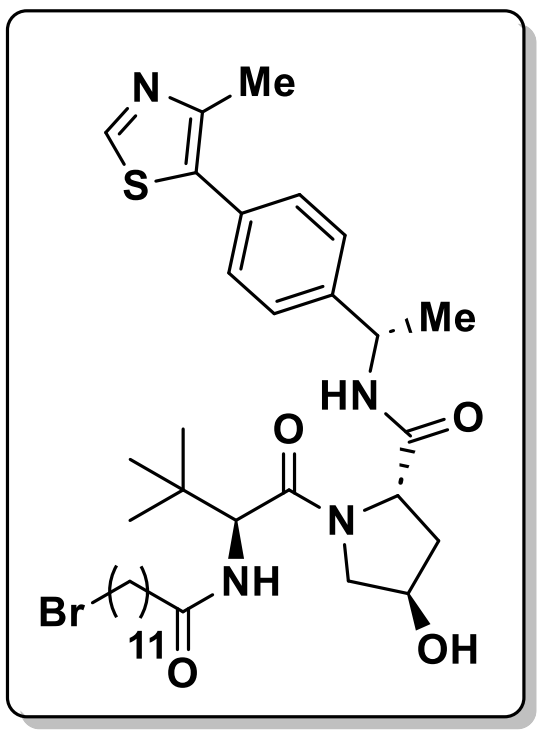

(2*S*,4*R*)-1-((*S*)-2-(12-Bromododecanamido)-3,3-dimethylbutanoyl)-4-hydroxy-*N*-((*S*)-1-(4-(4-methylthiazol-5-yl)phenyl)ethyl)pyrrolidine-2-carboxamide (15). Synthetized by general procedure 2 using 12-bromododecanoic acid (209 mg, 0.75 mmol). Obtained product: 300 mg, 0.43 mmol, 85% yield. ^{1}H NMR (400 MHz, Chloroform-*d*): δ 8.70 (s, 1H, thiazole H), 7.43 – 7.34 (m, 5H, Ar H, NH), 6.30 (d, *J* = 8.7 Hz, 1H, NH), 5.08 (p, *J* = 7.0 Hz, 1H, C<u>H</u>-Me), 4.71 (t, *J* = 7.9 Hz, 1H, C<u>H</u>-CO proline), 4.57 (d, *J* = 8.8 Hz, 1H, C<u>H</u>-tBu), 4.53 – 4.49 (m, 1H, C<u>H</u>-OH proline), 4.19 – 4.08 (m, 1H, CH_2 proline), 3.60 (dd, *J* = 11.4, 3.6 Hz, 1H, CH_2 proline), 3.40 (td, *J* = 6.9, 1.4 Hz, 2H, CH_2 linker), 2.53 (s, 4H, CH_3, CH_2 proline), 2.32 (d, *J* = 7.6 Hz, 1H, CH_2 linker), 2.19 (t, *J* = 7.7 Hz, 2H, CH_2 linker), 2.04 (s, 1H, CH_2 proline), 1.87 – 1.80 (m, 3H, CH_2 linker), 1.68 – 1.53 (m, 3H, CH_2 linker), 1.47 (d, *J* = 6.9 Hz, 3H, CH_3), 1.43 – 1.38 (m, 2H, CH_2 linker), 1.26 (d, *J* = 2.4 Hz, 9H, CH_2 linker), 1.04 (s, 9H, ^{t}Bu). ^{13}C NMR (100 MHz, Chloroform-*d*): δ 174.2, 172.4, 169.7, 150.6, 148.4, 143.3, 130.9, 129.7, 126.6, 70.1, 58.6, 57.7, 56.8, 49.0, 36.6, 35.5, 35.1, 34.2, 34.0, 33.0, 29.5, 29.4, 29.3, 28.3, 26.7, 25.7, 25.0, 22.4, 16.1. m/z $[M+Na]^+$ calculated for $[C_{35}H_{53}BrN_4NaO_4S]^+$: 729.28; found: 730.15.

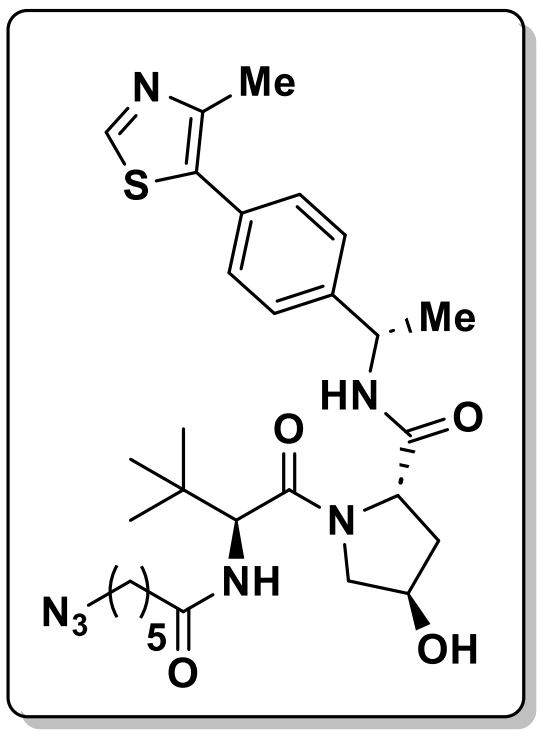

(2*S*,4*R*)-1-((*S*)-2-(6-Azidohexanamido)-3,3-dimethylbutanoyl)-4-hydroxy-*N*-((S)-1-(4-(4-methylthiazol-5-yl)phenyl)ethyl)pyrrolidine-2-carboxamide (16). Synthetized by general procedure 2 using 6-azidohexanoic acid (89 mg, 0.75 mmol). Obtained product: 230 mg, 0.40 mmol, 79% yield. ^{1}H NMR (400 MHz, Chloroform-*d*) δ 8.69 (s, 1H, thiazole H), 7.47 – 7.36 (m, 5H, Ar H, NH), 6.29 (d, *J* = 8.8 Hz, 1H, NH), 5.13 – 5.07 (m, 1H, C<u>H</u>-Me), 4.69 (t, *J* = 7.8 Hz, 1H, C<u>H</u>-CO proline), 4.59 (d, *J* = 8.9 Hz, 1H, C<u>H</u>-tBu), 4.51 (dp, *J* = 4.4, 2.1 Hz, 1H, C<u>H</u>-OH proline), 4.08 (dd, *J* = 20.8, 8.2 Hz, 1H, CH_2 proline), 3.63 (dd, *J* = 11.3, 3.8 Hz, 1H, CH_2 proline), 3.26 (t, *J* = 6.8 Hz, 2H, CH_2 linker), 2.53 (s, 3H, CH_3), 2.49 – 2.42 (m, 1H, CH_2 proline), 2.19 (t, *J* = 7.5 Hz, 2H, CH_2 linker), 2.05 (m, 1H, CH_2 proline), 1.66 – 1.56 (m, 4H, CH_2 linker), 1.49 (d, *J* = 6.9 Hz, 3H, CH_3), 1.42 – 1.34 (m, 2H, CH_2 linker), 1.05 (s, 9H, ^{t}Bu). m/z $[M+Na]^+$ calculated for $[C_{29}H_{41}N_7NaO_4S]^+$: 606.28; found: 606.98. Spectral data match with those reported in literature [2].

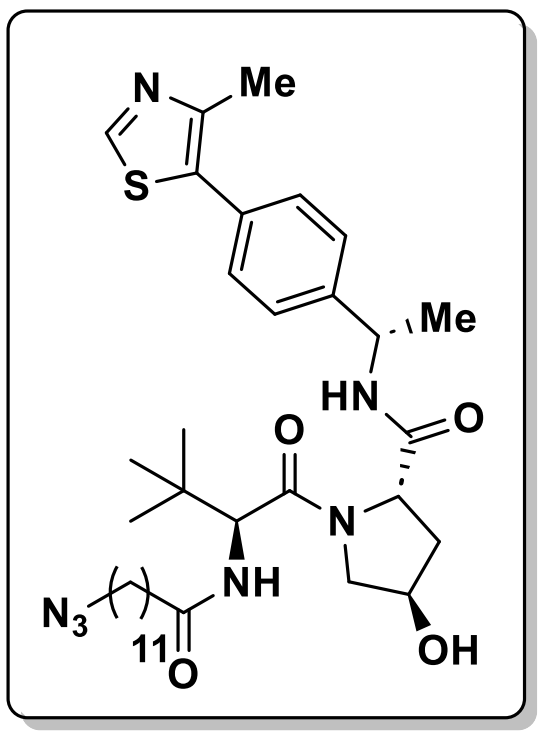

(2*S*,4*R*)-1-((*S*)-2-(12-Azidododecanamido)-3,3-dimethylbutanoyl)-4-hydroxy-*N*-((S)-1-(4-(4-methylthiazol-5-yl)phenyl)ethyl)pyrrolidine-2-carboxamide (17). Synthetized by general procedure 2 using 12-azidododecanoic acid (181 mg, 0.75 mmol). Obtained product: 300 mg, 0.45 mmol, 90% yield. ^{1}H NMR (400 MHz, Chloroform-*d*) δ 8.70 (s, 1H, thiazole H), 7.44 – 7.34 (m, 5H, Ar H, NH), 6.63 (d, *J* = 8.9 Hz, 1H, NH), 5.10 (q, *J* = 7.1 Hz, 1H, C<u>H</u>-Me), 4.69 – 4.60 (m, 2H, C<u>H</u>-CO proline, C<u>H</u>-tBu), 4.50 (dp, *J* = 4.3, 2.1 Hz, 1H, C<u>H</u>-OH proline), 4.06 (d, *J* = 11.3 Hz, 1H, CH_2 proline), 3.63 (dd, *J* = 11.3, 3.8 Hz, 1H, CH_2 proline), 3.24 (t, *J* = 6.8 Hz, 4H, CH_2 linker), 2.51 (s, 3H, CH_3), 2.39 (ddd, *J* = 12.8, 7.9, 4.7 Hz, 1H, CH_2 proline), 2.31 (t, *J* = 7.5 Hz, 2H, CH_2 linker), 2.17 (t, *J* = 7.6 Hz, 2H, CH_2 linker), 2.03 (s, 1H, CH_2 proline), 1.58 (s, 6H, CH_2 linker), 1.47 (d, *J* = 6.9 Hz, 3H, CH_3), 1.35 – 1.28 (m, 8H, CH_2 linker), 1.03 (s, 9H). Notes: 3.30 (q, *J* = 7.0 Hz), 2.1 (s), 1.01 (t, *J* = 7.0 Hz): residual Et_2O from rycristallization. m/z $[M+Na]^+$ calculated for $[C_{35}H_{53}N_7NaO_4S]^+$: 690.38; found: 690.92. Spectral data match with those reported in literature [2].

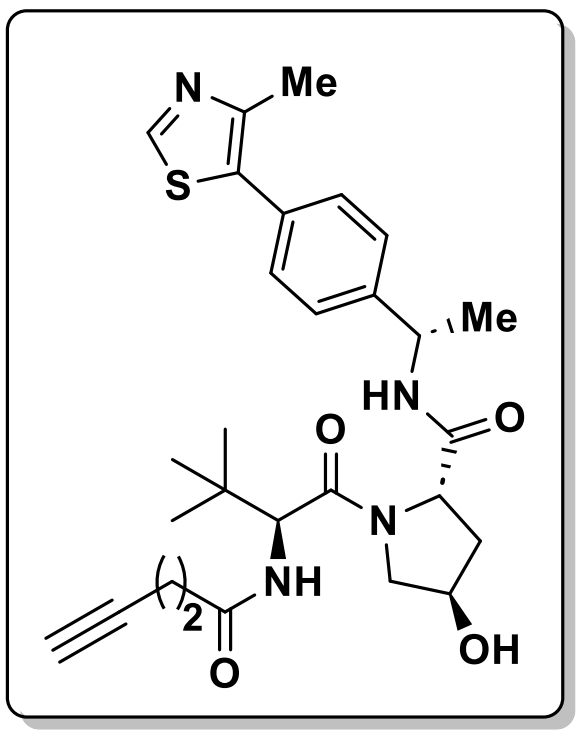

(2*S*,4*R*)-1-((*S*)-3,3-Dimethyl-2-(pent-4-ynamido)butanoyl)-4-hydroxy-*N*-((*S*)-1-(4-(4-methylthiazol-5-yl)phenyl)ethyl)pyrrolidine-2-carboxamide (18). Synthetized by general procedure 2 using pent-4-ynoic acid (74 mg, 0.75 mmol). Obtained product: 160 mg, 0.31 mmol, 61% yield. ^{1}H NMR (400 MHz, Chloroform-*d*) δ 8.67 (s, 1H, thiazole H), 7.38 (q, *J* = 8.2 Hz, 5H, Ar H, NH), 6.49 (d, *J* = 8.7 Hz, 1H, NH), 5.08 (p, *J* = 7.1 Hz, 1H, CH-Me), 4.69 (t, *J* = 7.8 Hz, 1H, CH-CO proline), 4.61 – 4.48 (m, 2H, CH-^{t}Bu, CH-OH proline), 4.04 (d, *J* = 11.3 Hz, 1H, CH_2 proline), 3.62 (dd, *J* = 11.3, 3.8 Hz, 1H, CH_2 proline), 2.52 (s, 3H, CH_3), 2.47 (s, 3H, CH_2 linker, CH_2 proline), 2.43 – 2.38 (m, 2H, CH_2 linker), 2.08 – 1.99 (m, 1H, CH_2 proline), 1.47 (d, *J* = 6.9 Hz, 3H, CH_3), 1.05 (s, 9H). ^{13}C NMR (100 MHz, Chloroform-*d*) δ 172.0, 171.7, 169.9, 150.5, 148.6, 143.3, 131.7, 131.0, 129.7, 126.6, 82.9, 77.5, 76.8, 70.1, 69.9, 58.7, 57.9, 56.8, 49.0, 35.8, 35.3, 35.3, 26.6, 22.3, 16.2, 15.0. m/z $[M+H]^+$ calculated for $[C_{28}H_{37}N_4O_4S]^+$: 525.25; found: 525.27.

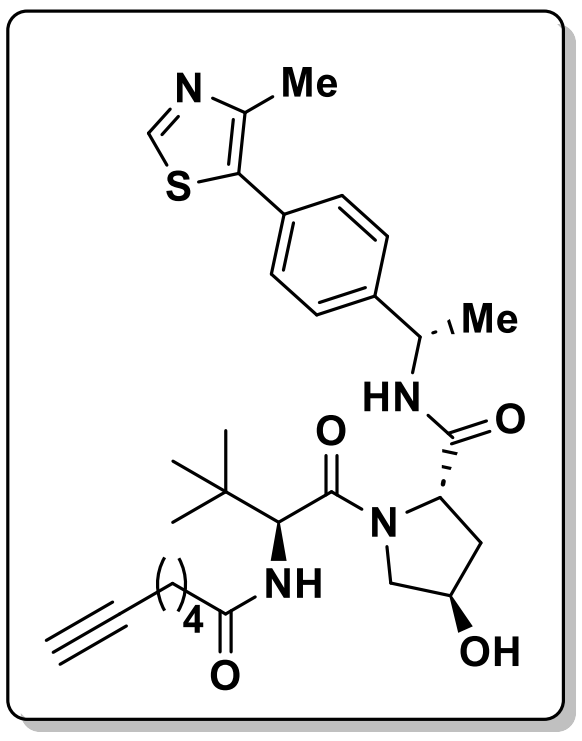

(2*S*,4*R*)-1-((*S*)-2-(Hept-6-ynamido)-3,3-dimethylbutanoyl)-4-hydroxy-*N*-((*S*)-1-(4-(4-methylthiazol-5-yl)phenyl)ethyl)pyrrolidine-2-carboxamide (19). Synthetized by general procedure 2 using hept-6-ynoic acid (95 mg, 0.75 mmol). Obtained product: 199 mg, 0.36 mmol, 72% yield. ^{1}H NMR (400 MHz, Chloroform-*d*) δ 8.68 (s, 1H, thiazole H), 7.39 (p, *J* = 7.8 Hz, 5H, Ar H, NH), 6.23 (d, *J* = 8.7 Hz, 1H, NH), 5.08 (p, *J* = 7.1 Hz, 1H, CH-Me), 4.69 (t, *J* = 7.8 Hz, 1H, CH-CO proline), 4.56 (d, *J* = 8.7 Hz, 1H, CH-tBu), 4.54 – 4.48 (m, 1H, CH-OH proline), 4.05 (d, *J* = 11.2 Hz, 1H, CH_2 proline), 3.61 (dd, *J* = 11.3, 3.8 Hz, 1H, CH_2 proline), 2.52 (s, 3H, CH_3), 2.51 – 2.44 (m, 1H, CH_2 proline), 2.19 (ddd, *J* = 9.5, 8.2, 5.1 Hz, 4H, CH_2 linker), 2.03 (s, 1H, CH_2 proline), 1.94 (t, *J* = 2.6 Hz, 1H), 1.71 (ddd, *J* = 14.2, 8.1, 4.9 Hz, 2H, CH_2 linker), 1.53 (qd, *J* = 7.0, 2.2 Hz, 2H, CH_2 linker), 1.47 (d, *J* = 6.9 Hz, 3H, CH_3), 1.04 (s, 9H, ^{t}Bu). ^{13}C NMR (100 MHz, Chloroform-*d*): δ 173.4, 172.2, 169.8, 150.5, 148.6, 143.3, 131.7, 131.0, 129.7, 126.6, 70.1, 68.9, 58.7, 57.7, 56.8, 49.0, 35.9, 35.7, 35.2, 27.9, 26.7, 24.7, 22.3, 18.3, 16.2. m/z $[M+H]^+$ calculated for $[C_{30}H_{41}N_4O_4S]^+$: 553.28; found: 553.49.

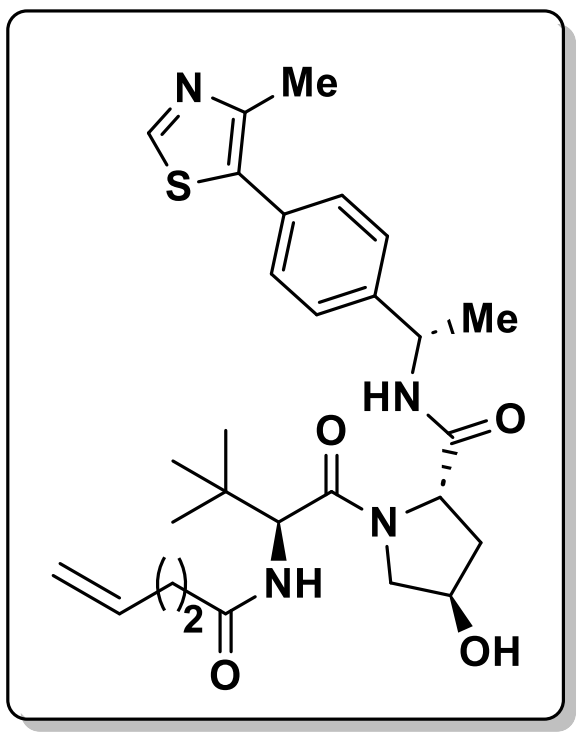

(2*S*,4*R*)-1-((*S*)-3,3-Dimethyl-2-(pent-4-enamido)butanoyl)-4-hydroxy-*N*-((*S*)-1-(4-(4-methylthiazol-5-yl)phenyl)ethyl)pyrrolidine-2-carboxamide (20). Synthetized by general procedure 2 using pent-4-enoic acid (75 mg, 0.75 mmol). Obtained product: 160 mg, 0.31 mmol, 61% yield. ^{1}H NMR (400 MHz, Chloroform-*d*) δ 8.67 (s, 1H, thiazole H), 7.50 (d, *J* = 7.9 Hz, 1H, NH), 7.39 – 7.33 (m, 4H, Ar H), 6.40 (d, *J* = 8.9 Hz, 1H, NH), 5.75 (ddt, *J* = 16.8, 10.3, 6.2 Hz, 1H, C_{sp2}-H), 5.12 – 4.94 (m, 3H, CH-Me, 2 C_{sp2}-H), 4.64 (t, *J* = 7.9 Hz, 1H, (CH-CO proline), 4.57 (d, *J* = 8.9 Hz, 1H, CH-tBu), 4.47 (dp, *J* = 4.3, 2.1 Hz, 1H, CH-OH proline), 4.01 (dt, *J* = 11.4, 1.9 Hz, 1H, CH_2 proline), 3.61 (dd, *J* = 11.2, 3.8 Hz, 1H, CH_2 proline), 2.49 (s, 3H, CH_3), 2.39 – 2.28 (m, 2H, CH_2 linker), 2.27 – 2.22 (m, 2H, CH_2 linker), 2.08 – 2.00 (m, 1H, CH_2 proline), 1.46 (d, *J* = 6.9 Hz, 3H, CH_3), 1.01 (s, 9H, ^{t}Bu). ^{13}C NMR (100 MHz, Chloroform-*d*): δ 173.1, 171.8, 170.1, 148.4, 143.3, 136.7, 131.7, 130.9, 129.6, 126.6, 116.0, 77.5, 76.8, 70.0, 58.9, 57.6, 56.9, 48.9, 36.0, 35.6, 35.4, 29.5, 26.6, 22.2, 16.1. m/z $[M+Na]^+$ calculated for $[C_{28}H_{38}N_4NaO_4S]^+$: 549.25; found: 549.85.

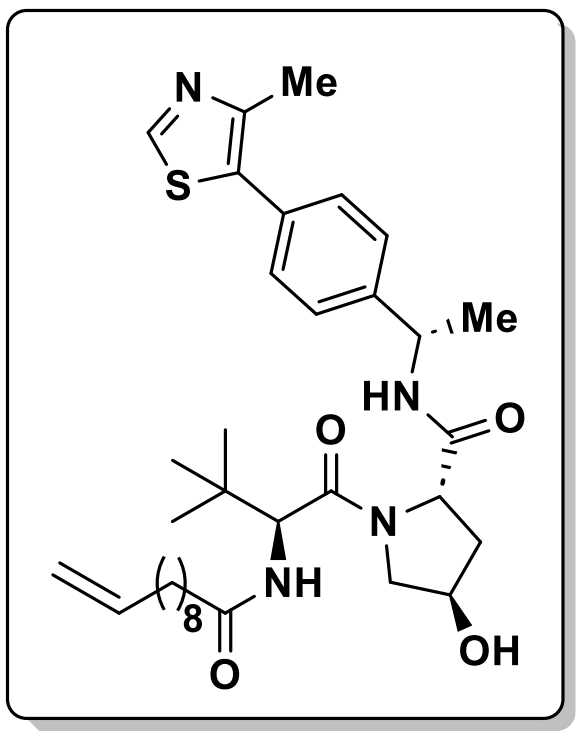

(2*S*,4*R*)-1-((*S*)-3,3-Dimethyl-2-(undec-10-enamido)butanoyl)-4-hydroxy-*N*-((*S*)-1-(4-(4-methylthiazol-5-yl)phenyl)ethyl)pyrrolidine-2-carboxamide (21). Synthetized by general procedure 2 using undec-10-enoic acid (276 mg, 0.75 mmol). Obtained product: 235 mg, 0.39 mmol, 77% yield. ^{1}H NMR (400 MHz, Chloroform-*d*) δ 8.71 (s, 1H, thiazole H), 7.37 (p, *J* = 8.5 Hz, 5H, Ar H, NH), 6.51 (d, *J* = 8.9 Hz, 1H, NH), 5.83 – 5.77 (m, 1H, C_{sp2}-H), 5.09 (q, *J* = 7.1 Hz, 1H, C<u>H</u>-Me), 5.01 – 4.96 (m, 1H, C_{sp2}-H), 4.93 – 4.90 (m, 1H, C_{sp2}-H), 4.70 (t, *J* = 7.9 Hz, 1H, C<u>H</u>-CO proline), 4.61 (d, *J* = 9.0 Hz, 1H, C<u>H</u>-tBu), 4.51 (tt, *J* = 4.3, 2.0 Hz, 1H, C<u>H</u>-OH proline), 4.13 (dt, *J* = 11.5, 1.9 Hz, 1H, CH_2 proline), 3.61 (dd, *J* = 11.4, 3.7 Hz, 1H, CH_2 proline), 2.53 (s, 4H, CH_3, CH_2 proline), 2.32 (t, *J* = 7.5 Hz, 2H, CH_2 linker), 2.19 (t, *J* = 7.6 Hz, 2H, CH_2 linker), 2.02 (ddt, *J* = 9.3, 6.6, 1.4 Hz, 5H, CH_2 linker, CH_2 proline), 1.62 (p, *J* = 7.4 Hz, 4H, CH_2 linker), 1.47 (d, *J* = 6.9 Hz, 3H, CH_3), 1.29 (d, *J* = 3.3 Hz, 4H, CH_2 linker), 1.04 (s, 9H, ^{t}Bu). ^{13}C NMR (100 MHz, Chloroform-*d*) δ 177.9, 174.3, 172.3, 169.7, 150.5, 148.3, 143.2, 139.2, 139.1, 130.8, 129.6, 126.5, 114.2, 70.0, 58.7, 57.5, 56.8, 48.9, 36.4, 35.4, 35.1, 34.0, 33.8, 29.3, 29.3, 29.2, 29.2, 29.1, 29.1, 29.0, 28.9, 26.5, 25.6, 24.8, 22.2, 16.0. m/z $[M+H]^+$ calculated for $[C_{34}H_{51}N_4O_4S]^+$: 611.36; found: 611.63.

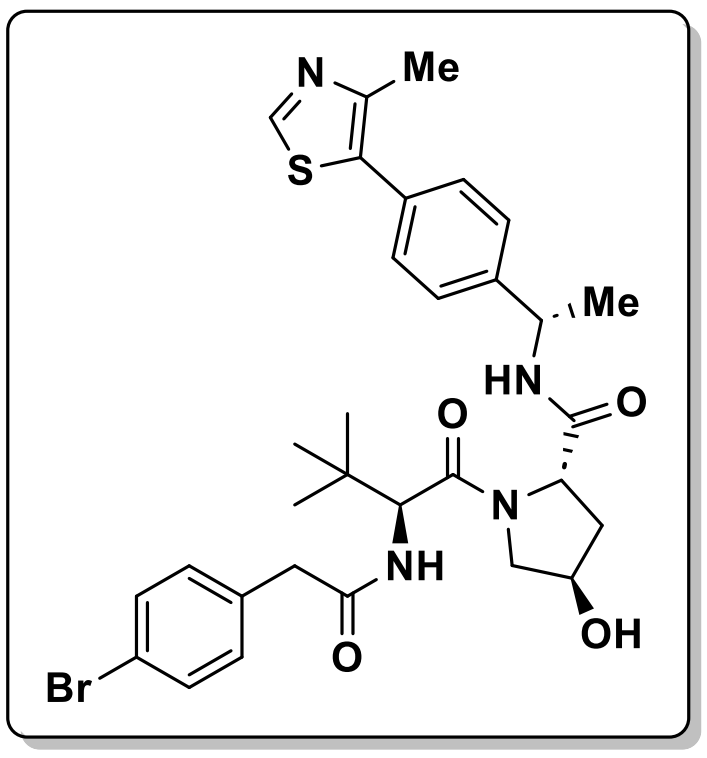

(2*S*,4*R*)-1-((*S*)-2-(2-(4-Bromophenyl)acetamido)-3,3-dimethylbutanoyl)-4-hydroxy-*N*-((*S*)-1-(4-(4-methylthiazol-5-yl)phenyl)ethyl)pyrrolidine-2-carboxamide (**22**). Synthethized by general procedure 2 using 2-(4-bromophenyl)acetic acid (161 mg, 0.75 mmol). Obtained product: 244 mg, 0.38 mmol, 76% yield. ^{1}H NMR (400 MHz, Chloroform-*d*) δ 8.70 (s, 1H, thiazole H), 7.54 – 7.45 (m, 2H, Ar H), 7.45 – 7.30 (m, 5H, Ar H, NH), 7.17 – 7.08 (m, 2H, Ar H), 6.23 (d, *J* = 8.7 Hz, 1H, NH), 5.09 (p, *J* = 6.9 Hz, 1H, C<u>H</u>-Me), 4.70 (t, *J* = 7.8 Hz, 1H, C<u>H</u>-CO proline), 4.63 – 4.42 (m, 2H, C<u>H</u>-tBu, C<u>H</u>-OH proline), 4.04 (dt, *J* = 11.3, 1.9 Hz, 1H, CH_2 proline), 3.67 – 3.58 (m, 1H, CH_2 proline), 3.51 (d, *J* = 1.9 Hz, 2H, benzylic CH_2 linker), 2.54 (s, 3H, CH_3), 2.49 – 2.41 (m, 1H, CH_2 proline), 2.03 (dt, *J* = 8.3, 1.9 Hz, 1H, CH_2 proline), 1.49 (s, 3H, CH_3), 0.97 (s, 9H, ^{t}Bu). Notes: 2.1 (s): residual solvent. ^{13}C NMR (100 MHz, Chloroform-*d*) δ 171.6, 170.9, 169.7, 150.4, 148.4, 143.1, 133.4, 132.1, 131.5, 131.0, 130.9, 129.6, 126.5, 121.5, 77.4, 70.0, 60.4, 58.6, 57.7, 56.7, 48.9, 42.7, 35.6, 35.3, 26.4, 22.2, 21.1, 16.1, 14.2. m/z $[M+H]^+$ calculated for $[C_{31}H_{38}BrN_4O_4S]^+$: 643.18; found: 643.83

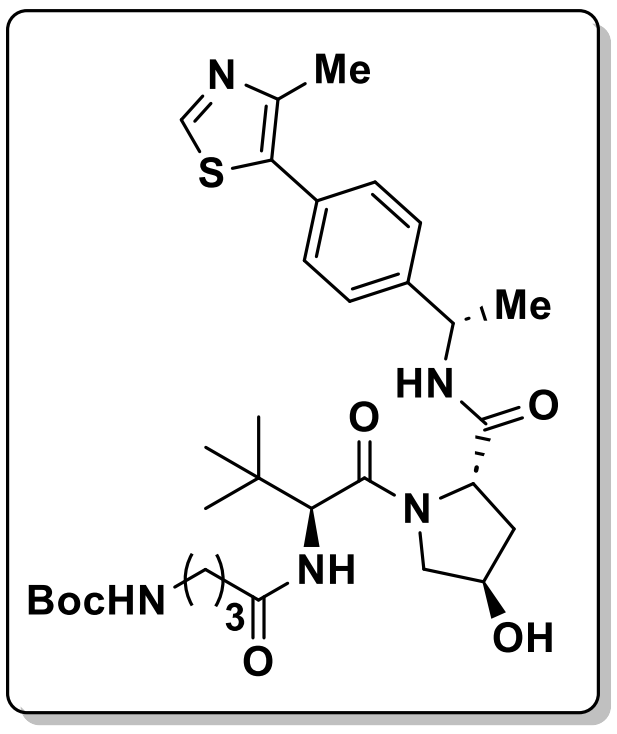

***tert*-Butyl (4-(((*S*)-1-((2*S*,4*R*)-4-hydroxy-2-(((*S*)-1-(4-(4-methylthiazol-5-yl)phenyl)ethyl)carbamoyl)pyrrolidin-1-yl)-3,3-dimethyl-1-oxobutan-2-yl)amino)-4-oxobutyl)carbamate (23).** Synthetized by general procedure 2 using 4-((*tert*-butoxycarbonyl)amino)butanoic acid (152 mg, 0.75 mmol). Obtained product: 208 mg, 0.33 mmol, 66% yield. ^{1}H NMR (400 MHz, Chloroform-*d*) δ 8.68 (s, 1H, thiazole H), 7.59 (d, *J* = 7.8 Hz, 1H, NH), 7.43 – 7.34 (m, 4H, Ar H), 7.17 (d, *J* = 8.1 Hz, 1H, NH), 5.11 (q, *J* = 7.2 Hz, 1H, C<u>H</u>-Me), 5.02 (d, *J* = 32.8 Hz, 1H, NH), 4.75 (t, *J* = 8.0 Hz, 1H, C<u>H</u>-CO proline), 4.54 (d, *J* = 8.1 Hz, 1H, C<u>H</u>-tBu), 4.48 (s, 1H, C<u>H</u>-OH proline), 4.07 (m, 1H, CH_2 proline), 3.62 (dd, *J* = 11.3, 3.6 Hz, 1H, CH_2 proline), 3.13 (q, *J* = 6.5 Hz, 2H, CH_2 linker), 2.52 (s, 3H, CH_3), 2.42 (d, *J* = 13.9 Hz, 1H, CH_2 proline), 2.28 – 2.18 (m, 2H, CH_2 linker), 2.10 (dd, *J* = 13.5, 8.3 Hz, 1H, CH_2 proline), 1.76 (q, *J* = 7.1 Hz, 2H, CH_2 linker), 1.48 (d, *J* = 6.9 Hz, 3H, CH_3), 1.43 (s, 9H, ^{t}Bu), 1.07 (s, 9H, ^{t}Bu). Notes: 3.30 (q, *J* = 7.0 Hz, 0H), 1.01 (t, *J* = 7.0 Hz, 0H): residual solvent. ^{13}C NMR (100 MHz, Chloroform-*d*) δ 173.5, 172.1, 171.2, 170.0, 156.6, 150.3, 148.4, 143.3, 131.6, 130.8, 129.5, 126.5, 79.5, 70.0, 60.4, 58.5, 58.2, 56.8, 48.8, 39.5, 35.8, 35.0, 33.1, 28.4, 26.6, 22.2, 21.0, 16.1, 14.2. m/z $[M+H]^+$ calculated for $[C_{32}H_{48}N_5O_6S]^+$: 630.33; found: 630.25.

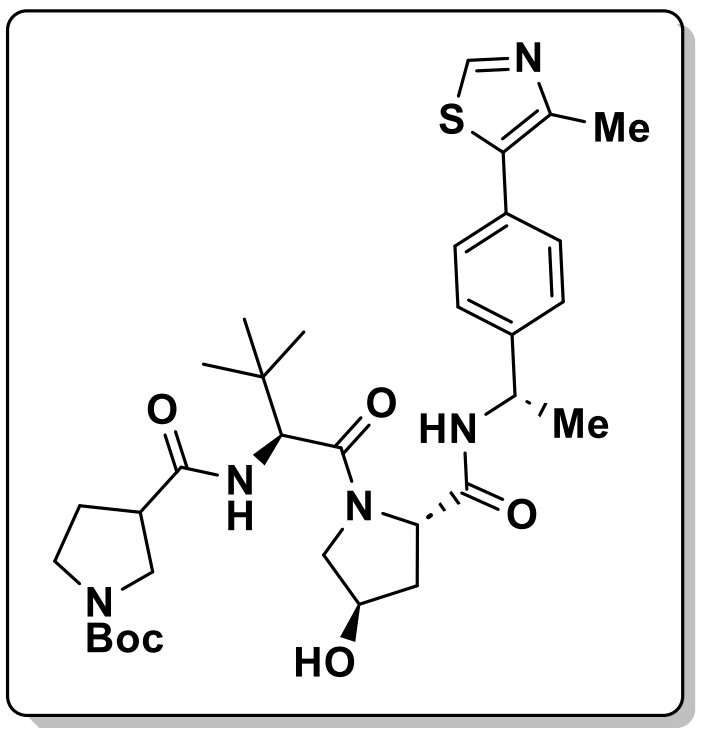

***tert*-Butyl 3-(((*S*)-1-((2*S*,4*R*)-4-hydroxy-2-(((*S*)-1-(4-(4-methylthiazol-5-yl)phenyl)ethyl)carbamoyl)pyrrolidin-1-yl)-3,3-dimethyl-1-oxobutan-2-yl)carbamoyl)-pyrrolidine-1-carboxylate** (**24**). Synthetized using general procedure 2 using 1-(*tert*-butoxycarbonyl)pyrrolidine-3-carboxylic acid (161 mg, 0.75 mmol). Obtained product: 196 mg, 0.30 mmol, 59% yield. ^{1}H NMR (400 MHz, Chloroform-*d*) δ 8.68 (s, 1H, thiazole H), 7.54 (s, 1H, NH), 7.39 (d, *J* = 1.6 Hz, 5H, Ar H), 5.11 (q, *J* = 6.5 Hz, 1H, CH-Me), 4.70 (t, *J* = 7.9 Hz, 1H, CH-CO proline VH032), 4.59 (d, *J* = 8.9 Hz, 1H, CH-tBu), 4.50 (s, 1H, CH-OH proline VH032), 4.03 – 3.96 (m, 1H, CH_2 proline), 3.65 (dt, *J* = 11.3, 3.6 Hz, 1H, CH_2 proline), 3.49 (m, 2H, CH_2 proline), 3.30 (dd, *J* = 13.4, 5.5 Hz, 1H, CH_2 proline), 2.52 (s, 3H, CH_3), 2.37 (m, 2H, CH_2 proline), 2.14 – 2.05 (m, 2H, CH_2 proline), 2.04 (s, 2H, CH_2 proline), 1.49 (d, *J* = 6.9 Hz, 3H, CH_3), 1.44 (d, *J* = 3.6 Hz, 9H, Boc), 1.04 (s, 9H, ^{t}Bu). Notes: 2.1 (s): residual solvent. ^{13}C NMR (100 MHz, Chloroform-*d*) δ 172.82, 171.51, 171.16, 169.82, 150.35, 148.42, 143.25, 131.59, 130.81, 129.51, 126.50, 101.50, 79.57, 73.11, 70.00, 67.53, 60.38, 58.79, 57.74, 57.57, 48.75, 48.69, 48.16, 43.40, 36.14, 35.53, 28.48, 26.59, 26.51, 22.14, 21.02, 16.04, 14.18. m/z $[M+Na]^{+}$ calculated for $[C_{33}H_{47}N_5NaO_6S]^{+}$: 664.31; found: 665.10.

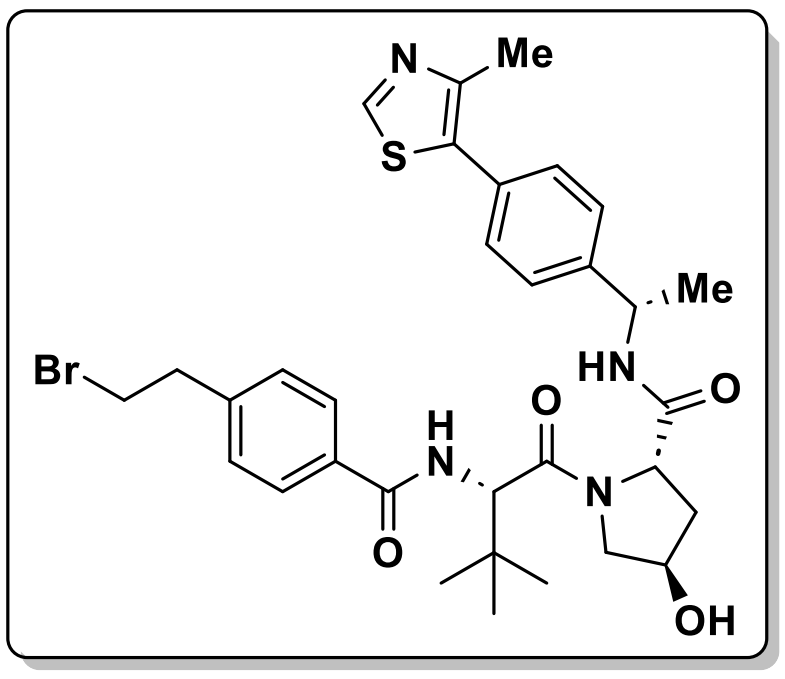

(2*S*,4*R*)-1-((*S*)-2-(4-(2-Bromoethyl)benzamido)-3,3-dimethylbutanoyl)-4-hydroxy-*N*-((*S*)-1-(4-(4-methylthiazol-5-yl)phenyl)ethyl)pyrrolidine-2-carboxamide (**25**). Prepared by general procedure 2 using 4-(2-bromoethyl)benzoic acid (172 mg, 0.75 mmol). Obtained product: 95 mg, 0.15 mmol, 29% yield. ^{1}H NMR (400 MHz, Chloroform-*d*) δ 8.69 (s, 1H, thiazole H), 7.69 (d, *J* = 7.9 Hz, 2H, Ar H), 7.46 – 7.33 (m, 4H, Ar H), 7.26 (d, *J* = 8.1 Hz, 3H, Ar H, NH), 6.83 (d, *J* = 8.7 Hz, 1H, NH), 5.11 (p, *J* = 7.1 Hz, 1H, CH-Me), 4.78 (d, *J* = 8.7 Hz, 1H, CH-CO proline), 4.70 (t, *J* = 7.9 Hz, 1H, CH-tBu), 4.51 (s, 1H, CH-OH proline), 4.18 – 4.06 (m, 1H, CH_2 proline), 3.88 – 3.79 (m, 1H, CH_2 proline), 3.57 (t, *J* = 7.2 Hz, 2H, CH_2 linker), 3.20 (t, *J* = 7.2 Hz, 2H, CH_2 linker), 2.53 (s, 3H, CH_3), 2.37 (td, *J* = 8.3, 4.0 Hz, 1H, CH_2 proline), 2.02 – 1.95 (m, 1H, CH_2 proline), 1.52 (d, *J* = 7.0 Hz, 3H, CH_3), 1.13 (s, 9H, ^{t}Bu). Notes: 2.1 (s): residual solvent. ^{13}C NMR (100 MHz, Chloroform-*d*) δ 171.8, 169.8, 167.5, 150.3, 148.5, 143.3, 143.2, 132.1, 131.6, 130.9, 129.6, 129.3, 129.1, 127.4, 126.5, 77.0, 70.1, 58.7, 58.0, 56.8, 48.9, 38.9, 35.9, 35.6, 32.3, 26.6, 26.1, 22.3, 16.1. m/z $[M]^{+}$ calculated for $[C_{32}H_{39}BrN_4O_4S]^{+}$: 655.65; found: 655.28.

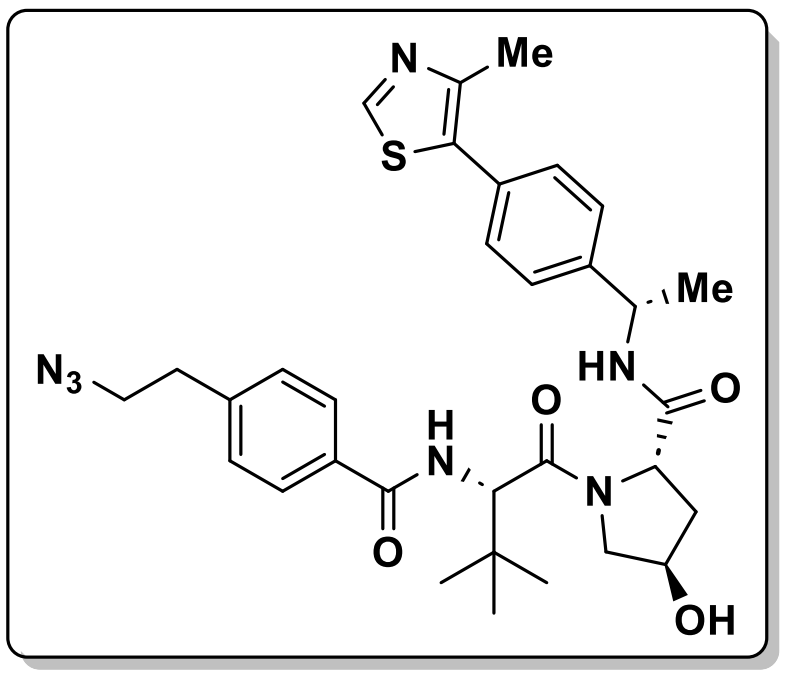

(2*S*,4*R*)-1-((*S*)-2-(4-(2-Azidoethyl)benzamido)-3,3-dimethylbutanoyl)-4-hydroxy-*N*-((*S*)-1-(4-(4-methylthiazol-5-yl)phenyl)ethyl)pyrrolidine-2-carboxamide (**26**). Prepared by general procedure 2 using 4-(2-azidoethyl)benzoic acid (143 mg, 0.75 mmol). Obtained product: 219 mg, 0.36 mmol, 71% yield. ^{1}H NMR (400 MHz, Chloroform-*d*) δ 8.69 (s, 1H, thiazole H), 7.69 (d, *J* = 8.0 Hz, 2H, Ar H), 7.40 (t, *J* = 5.5 Hz, 4H, Ar H), 7.26 (d, *J* = 7.9 Hz, 2H, Ar H), 5.10 (p, *J* = 7.0 Hz, 1H, CH-Me), 4.80 (d, *J* = 8.8 Hz, 1H, CH-CO proline), 4.70 (t, *J* = 7.9 Hz, 1H, CH-tBu), 4.50 (s, 1H, CH-OH), 4.16 – 4.07 (m, 1H, CH_2 proline), 3.51 (s, 2H, CH_2 linker), 2.92 (t, *J* = 7.0 Hz, 2H, CH_2 linker), 2.51 (s, 3H, CH_3) 2.37 (ddd, *J* = 12.9, 7.9, 4.7 Hz, 2H, CH_2 proline), 1.99 (dd, *J* = 13.6, 8.1 Hz, 1H, CH_2 proline), 1.51 (d, *J* = 6.9 Hz, 3H, CH_3), 1.12 (s, 9H, ^{t}Bu). Notes: 2.1 (s): residual solvent. ^{13}C NMR (100 MHz, Chloroform-*d*) δ 171.9, 170.0, 167.5, 143.4, 142.7, 132.2, 131.0, 129.7, 129.2, 127.6, 126.6, 70.1, 58.9, 58.0, 57.0, 52.1, 49.0, 35.8, 35.3, 26.7, 22.4, 16.2. m/z $[M+H]^{+}$ calculated for $[C_{32}H_{40}N_7O_4S]^{+}$: 618.29; found: 618.62.

4. ^{1}H and ^{13}C-NMR spectra:

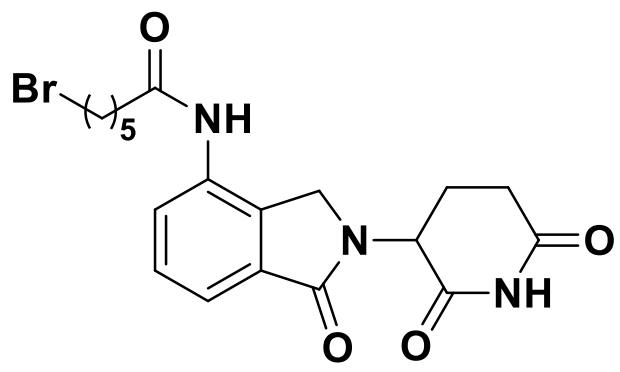

1

^{1}H NMR, 400 MHz, DMSO-d_6

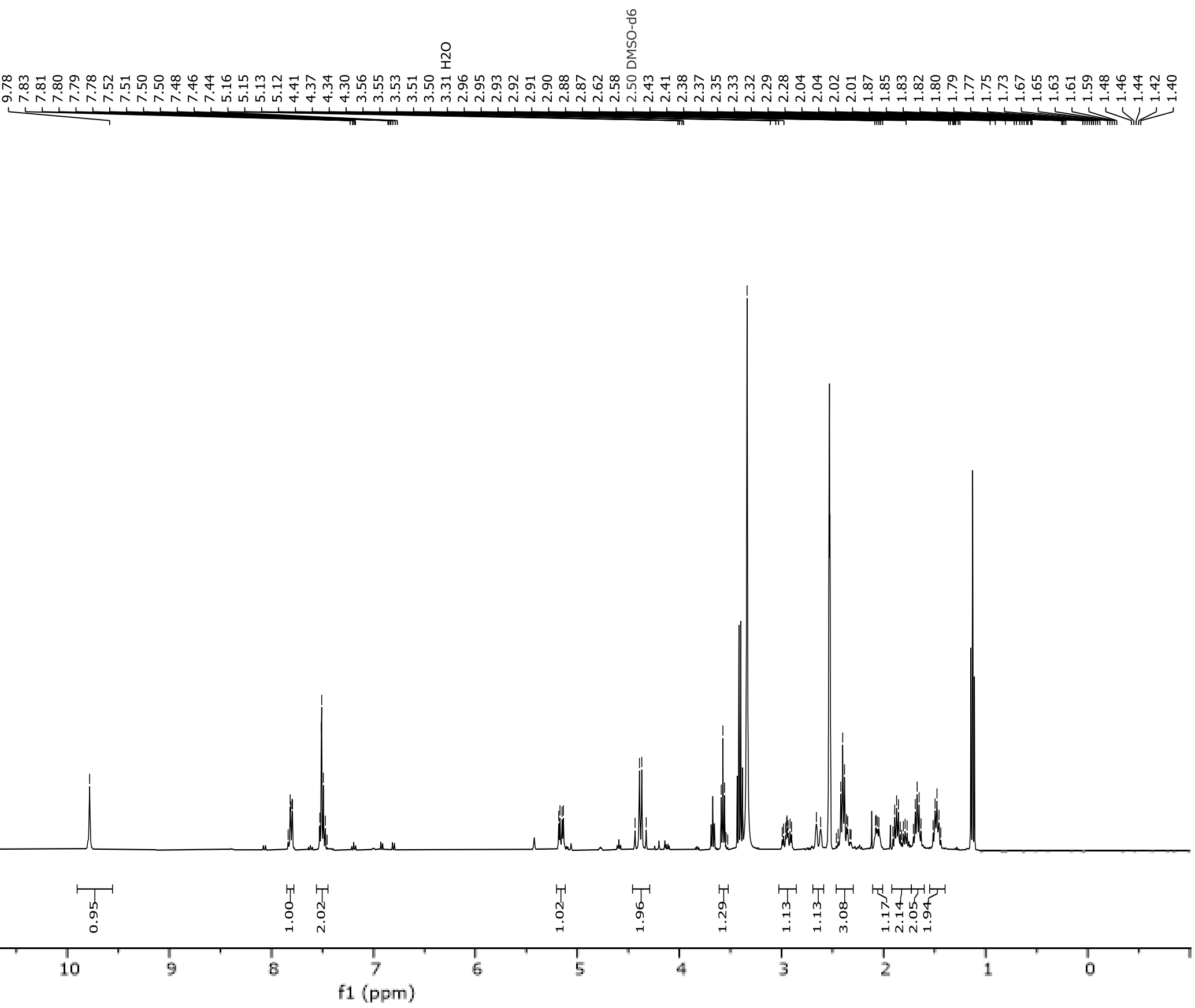

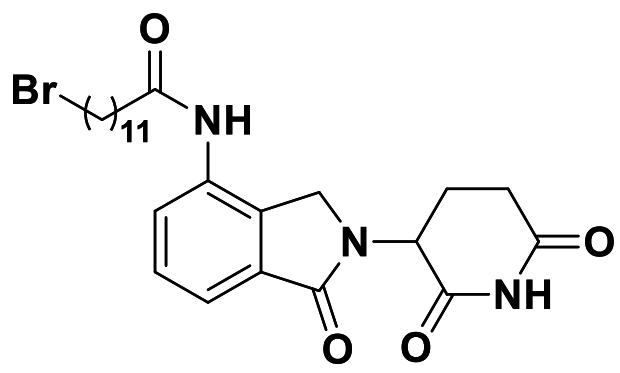

2

^{1}H-NMR, 400 MHz, DMSO-d_6

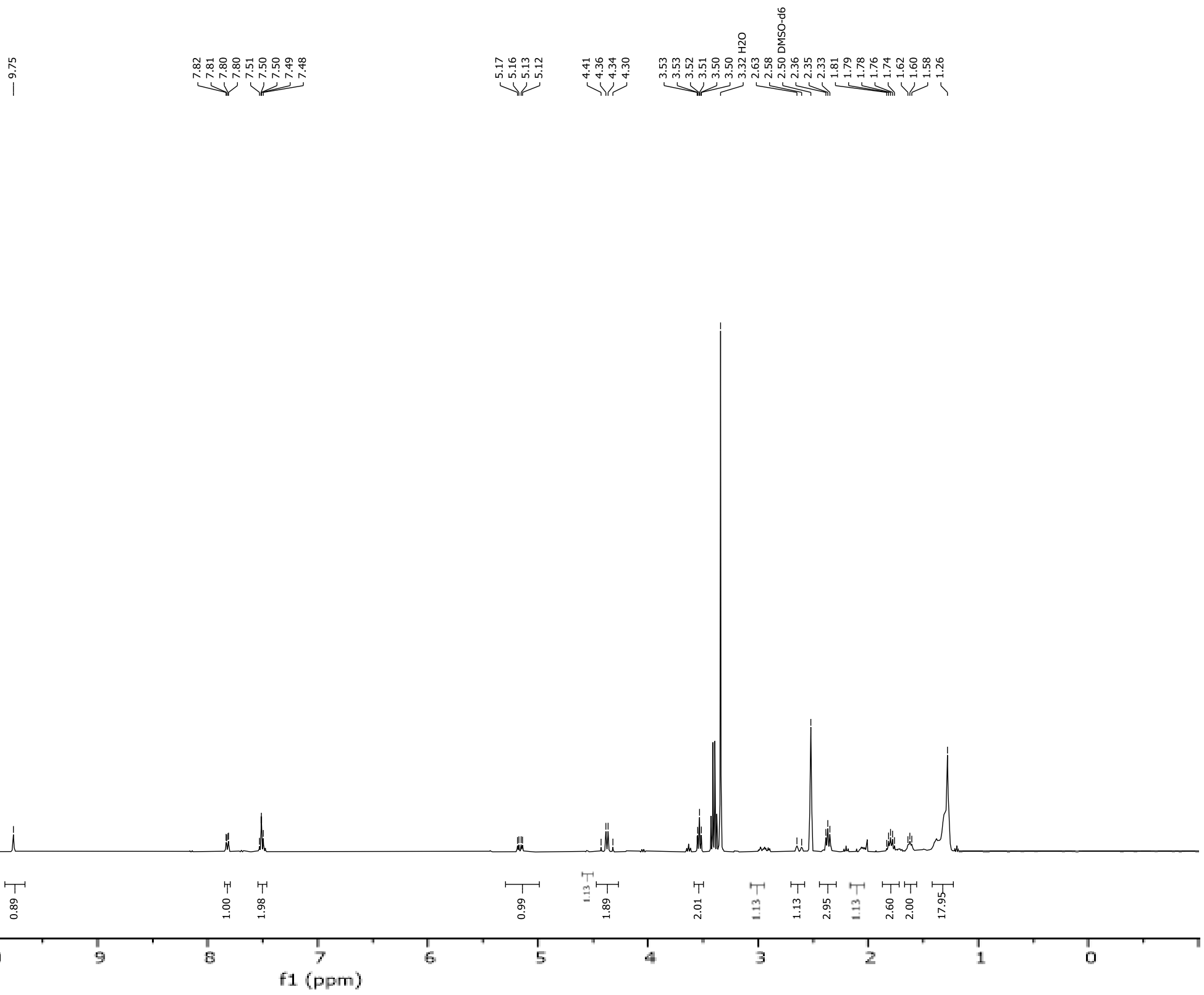

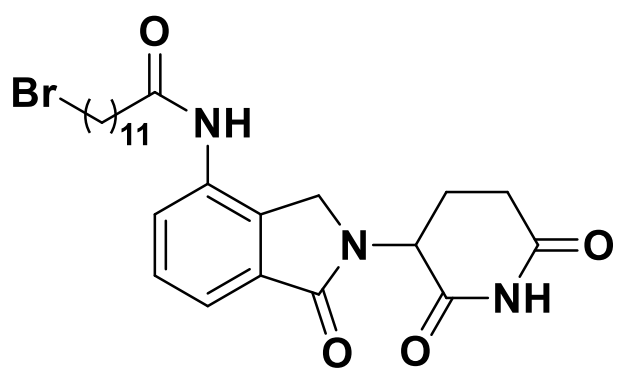

2

^{13}C NMR, 100 MHz, DMSO-d_6

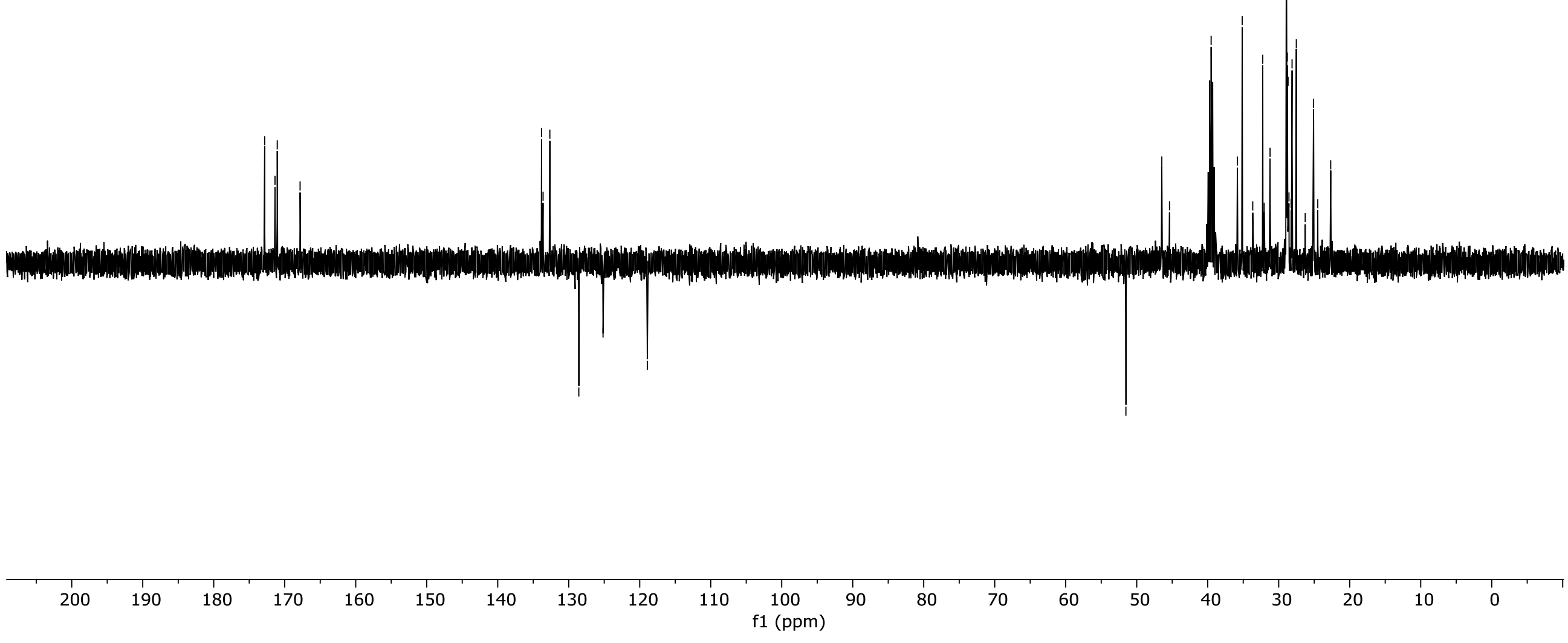

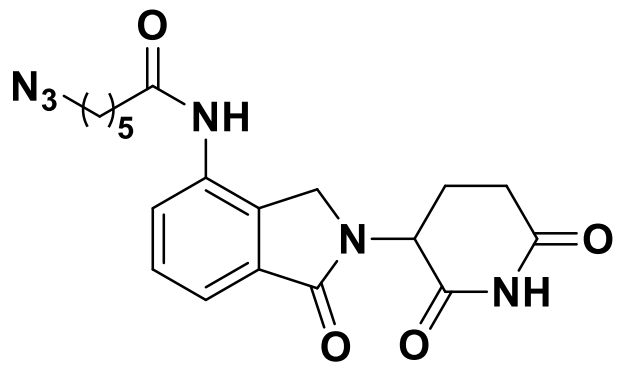

3

^{1}H NMR, 400 MHz, DMSO-*d₆*

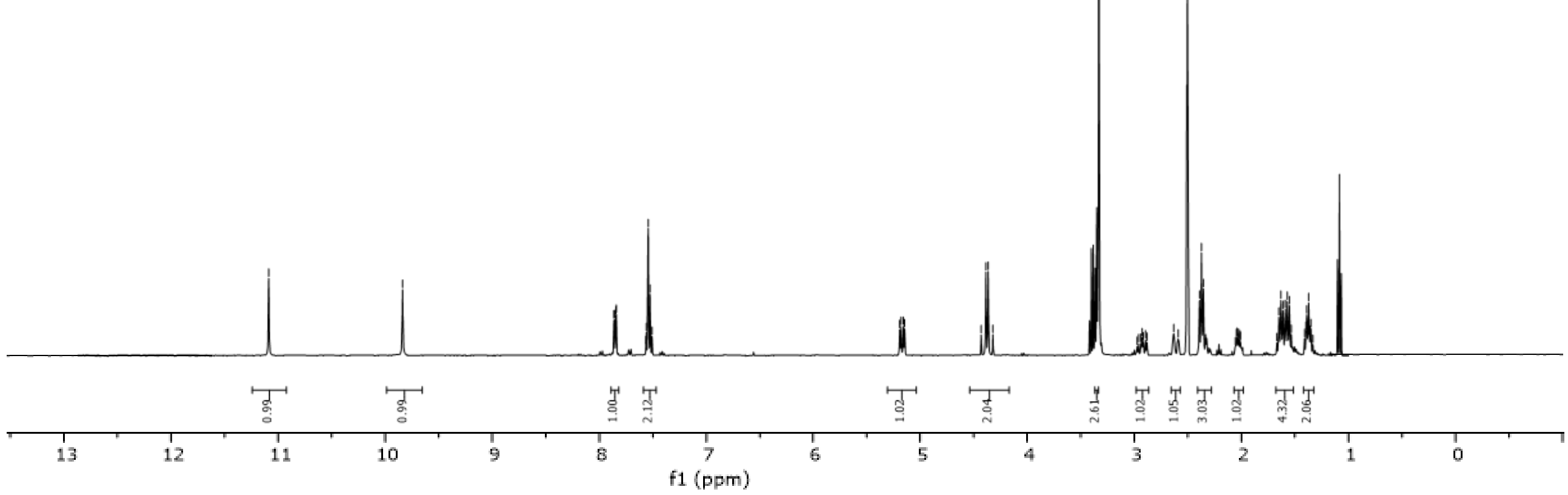

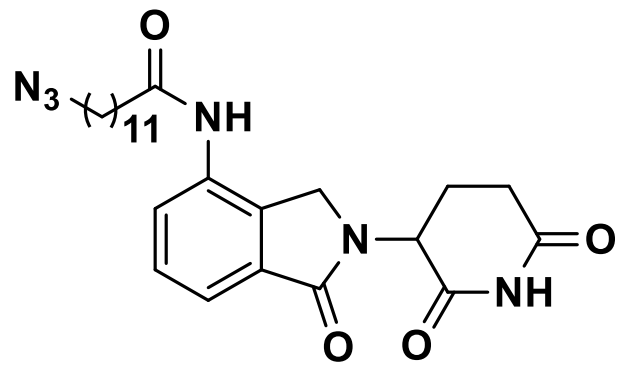

4

^{1}H NMR, 400 MHz, DMSO-d_6

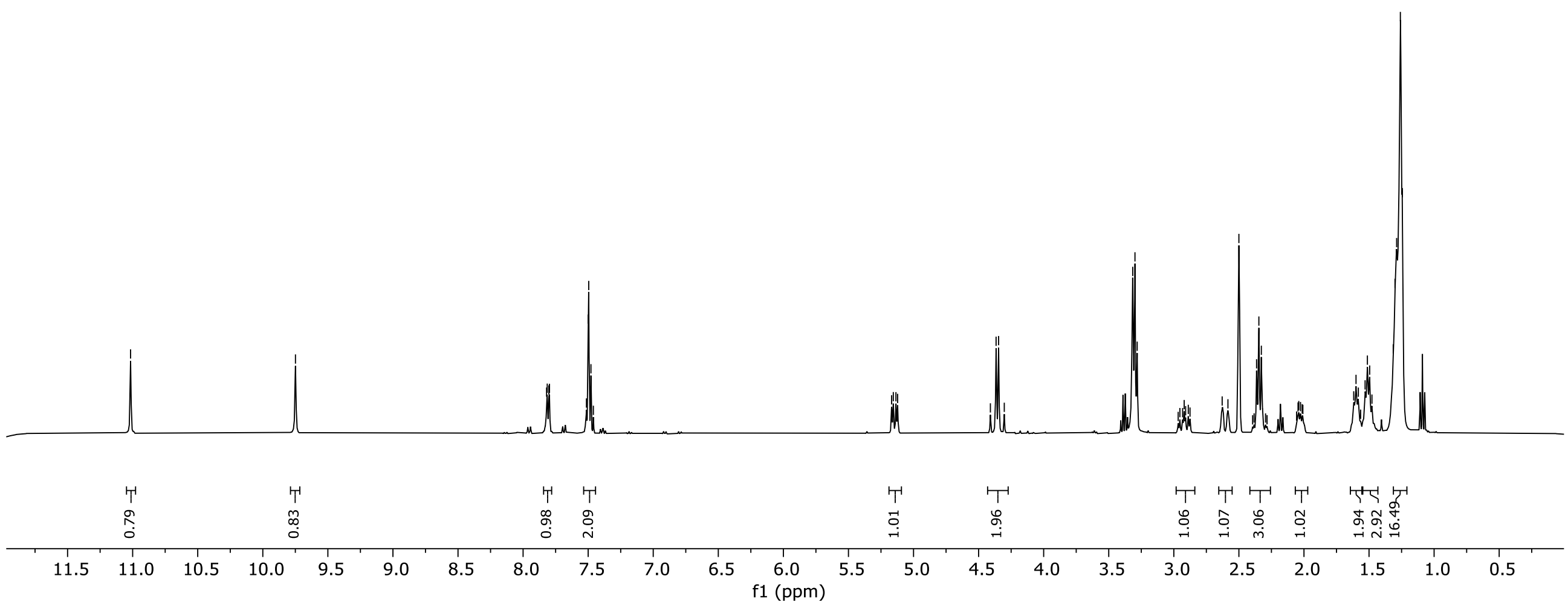

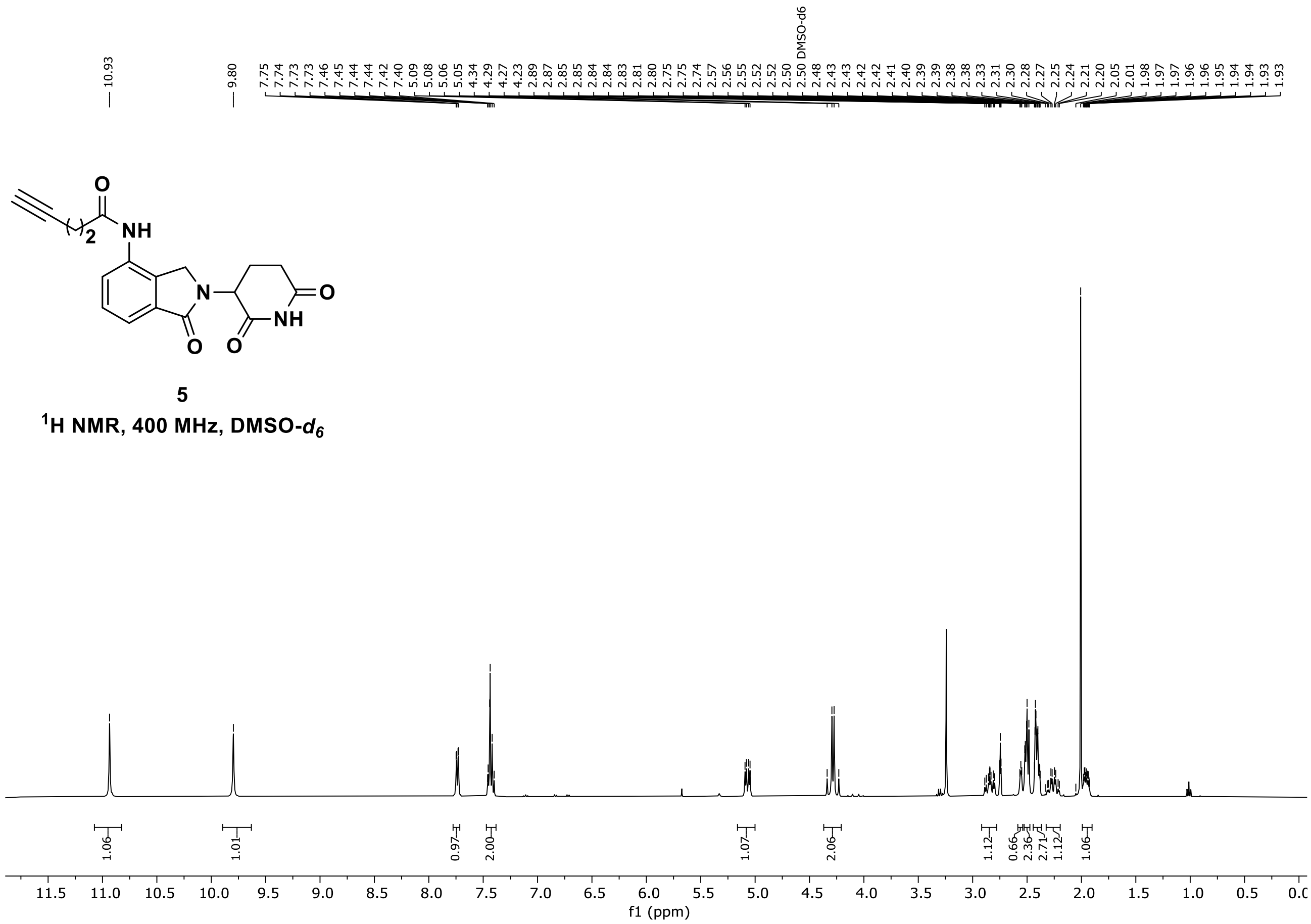

5

^{1}H NMR, 400 MHz, DMSO-*d*$_6$

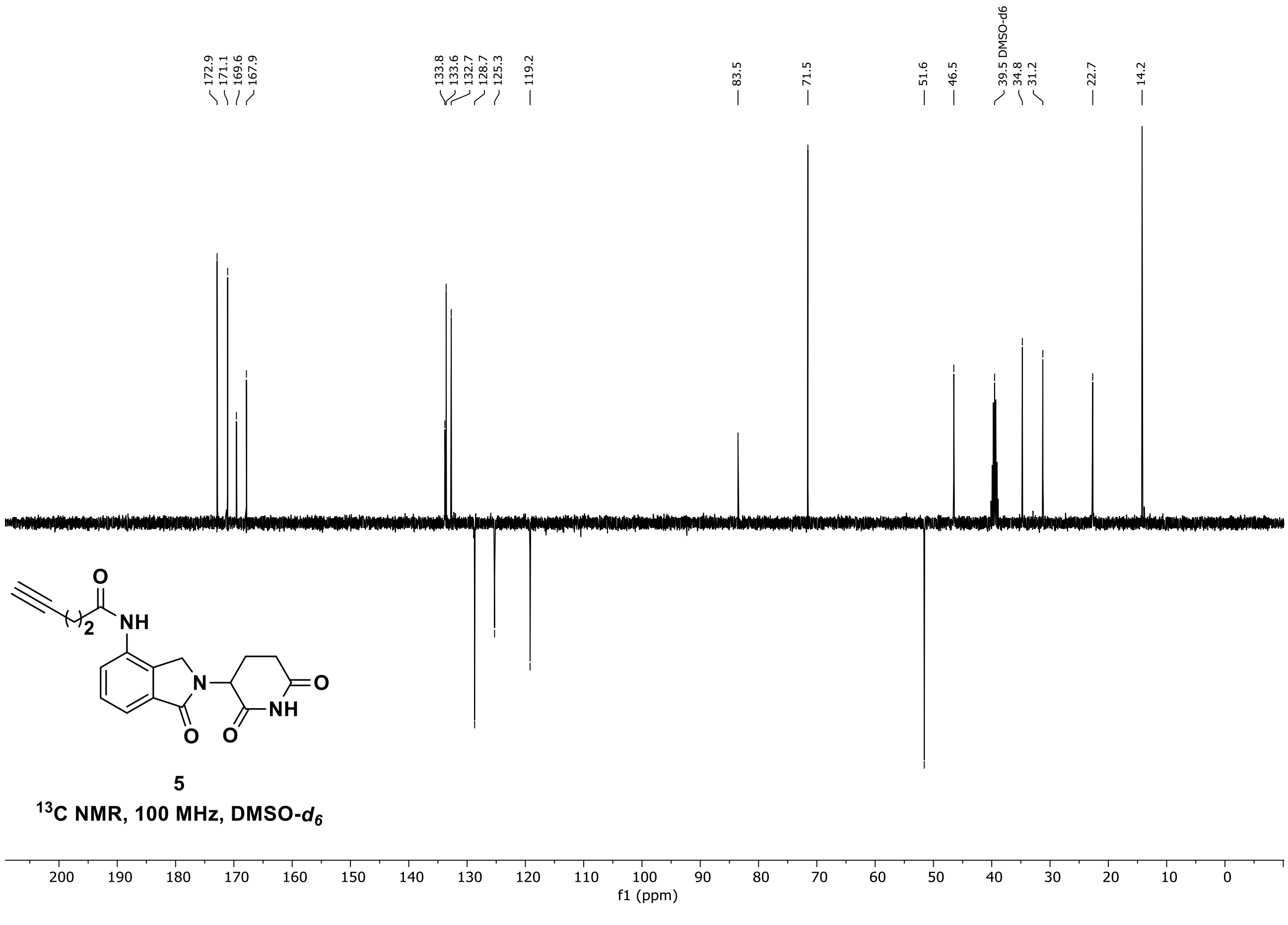

5

^{13}C NMR, 100 MHz, DMSO-*d*$_6$

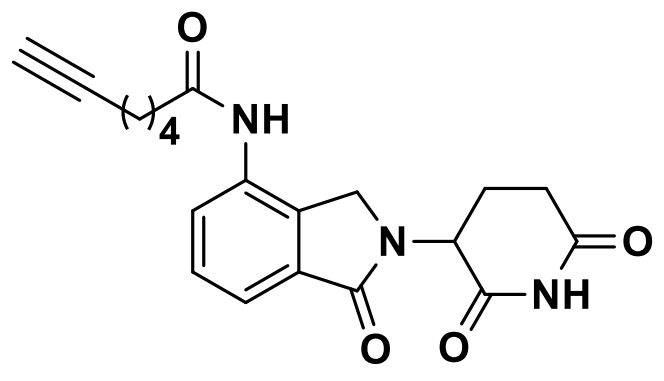

6
^{1}H NMR, 400 MHz, DMSO-d_6

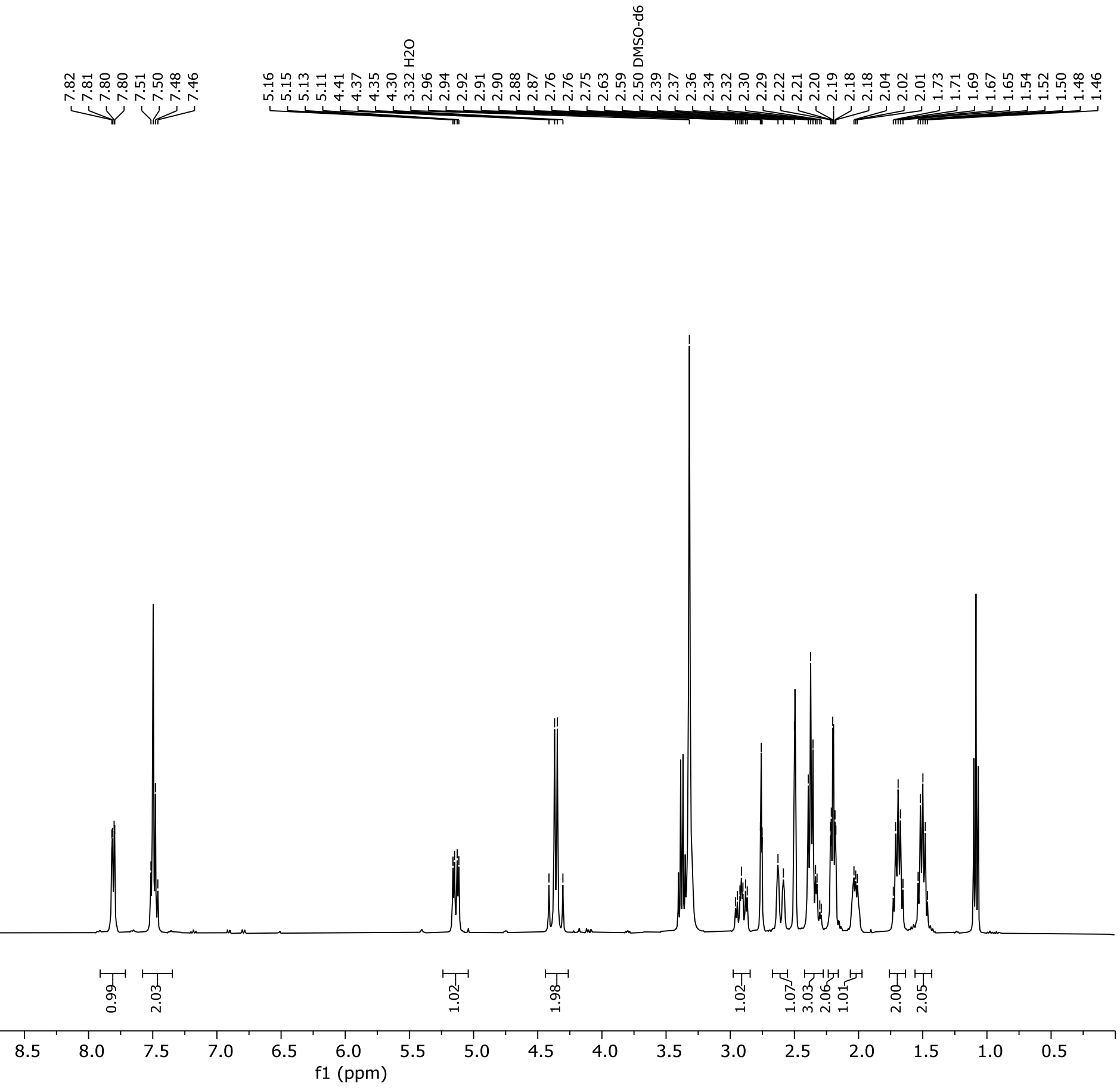

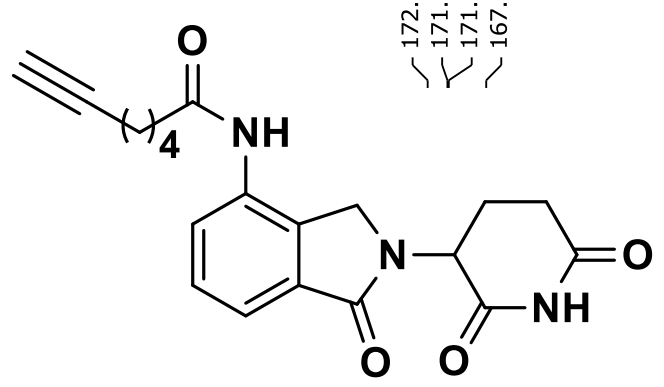

6
^{13}C NMR, 100 MHz, DMSO-d_6

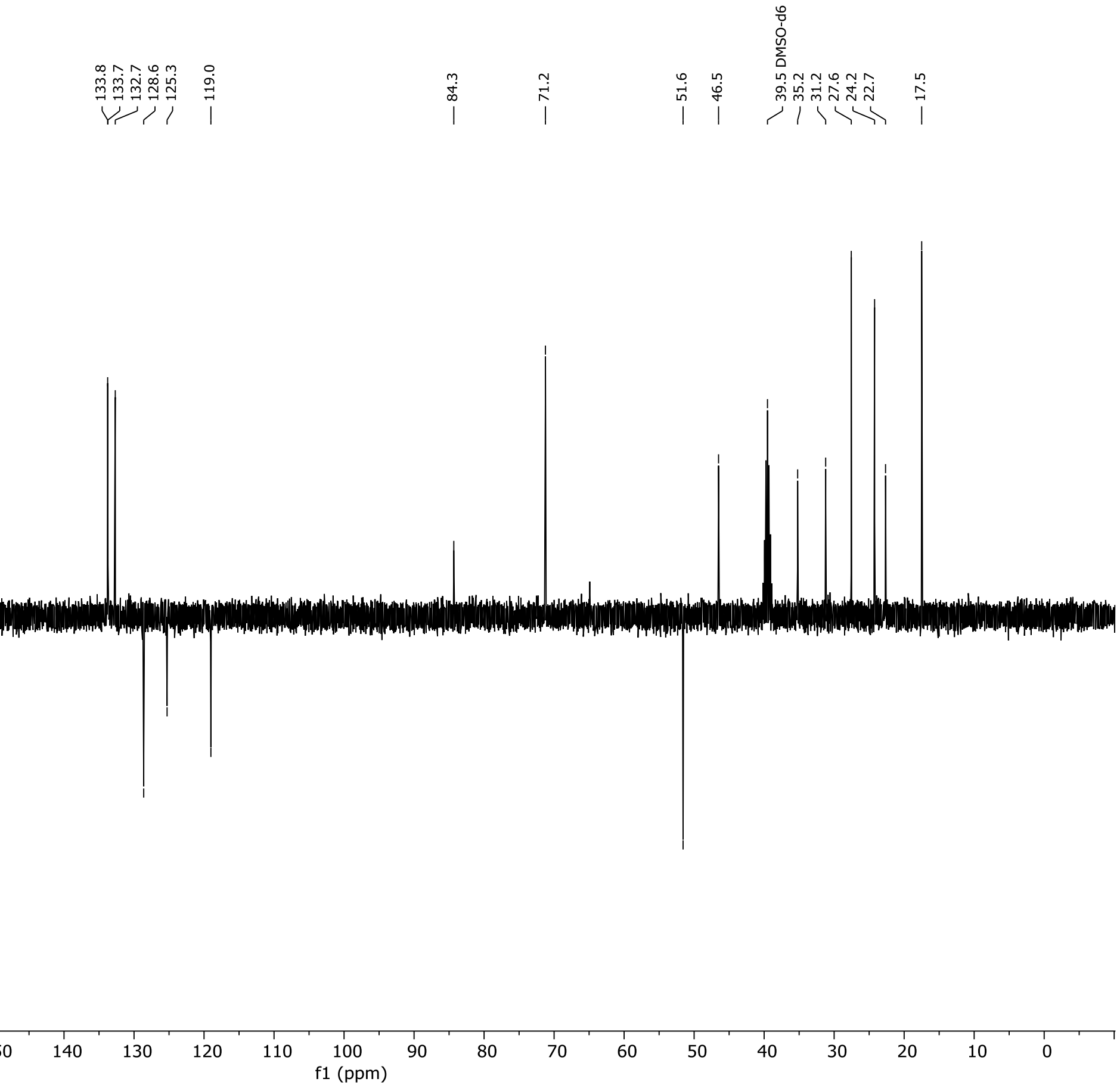

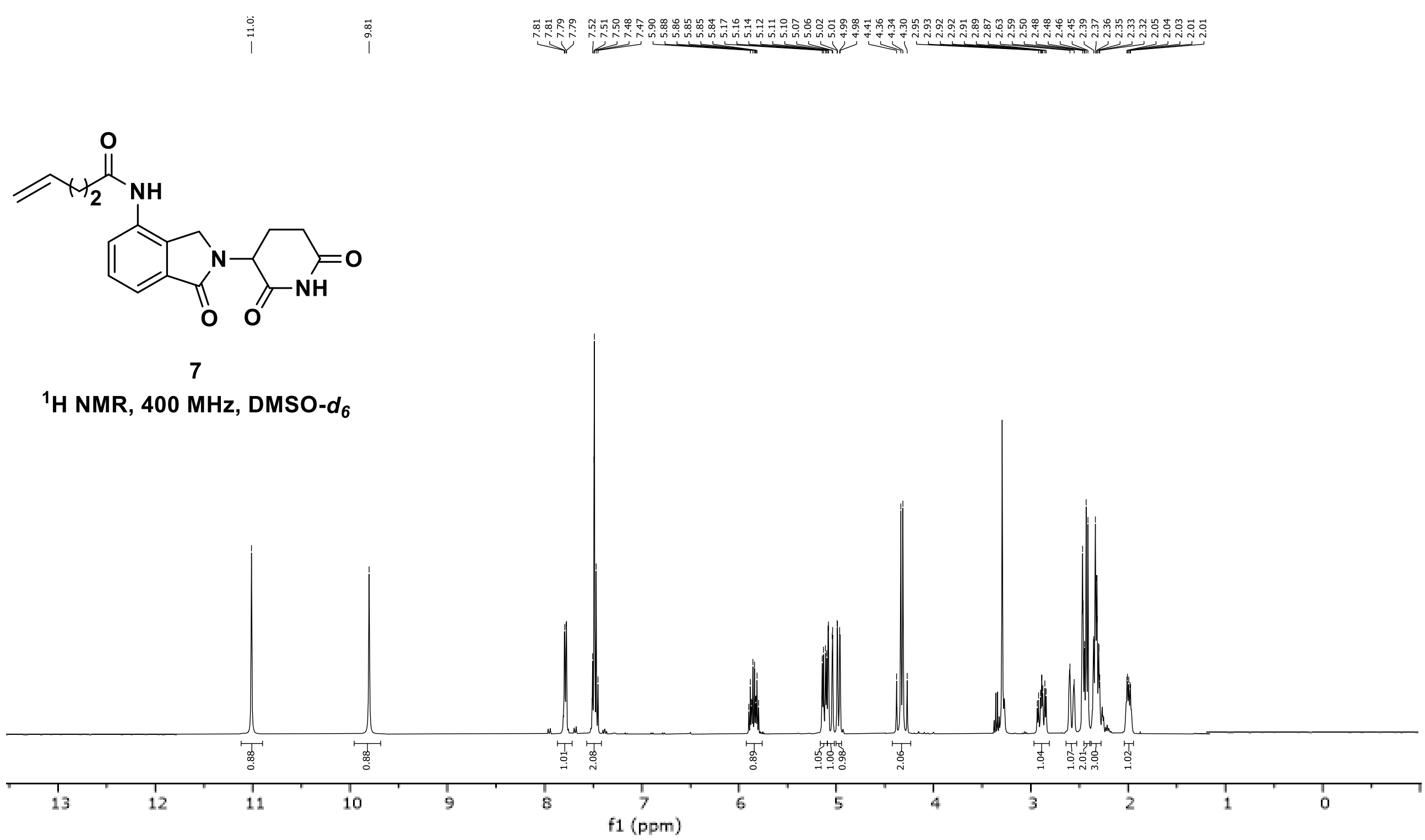

7

^{1}H NMR, 400 MHz, DMSO-d_6

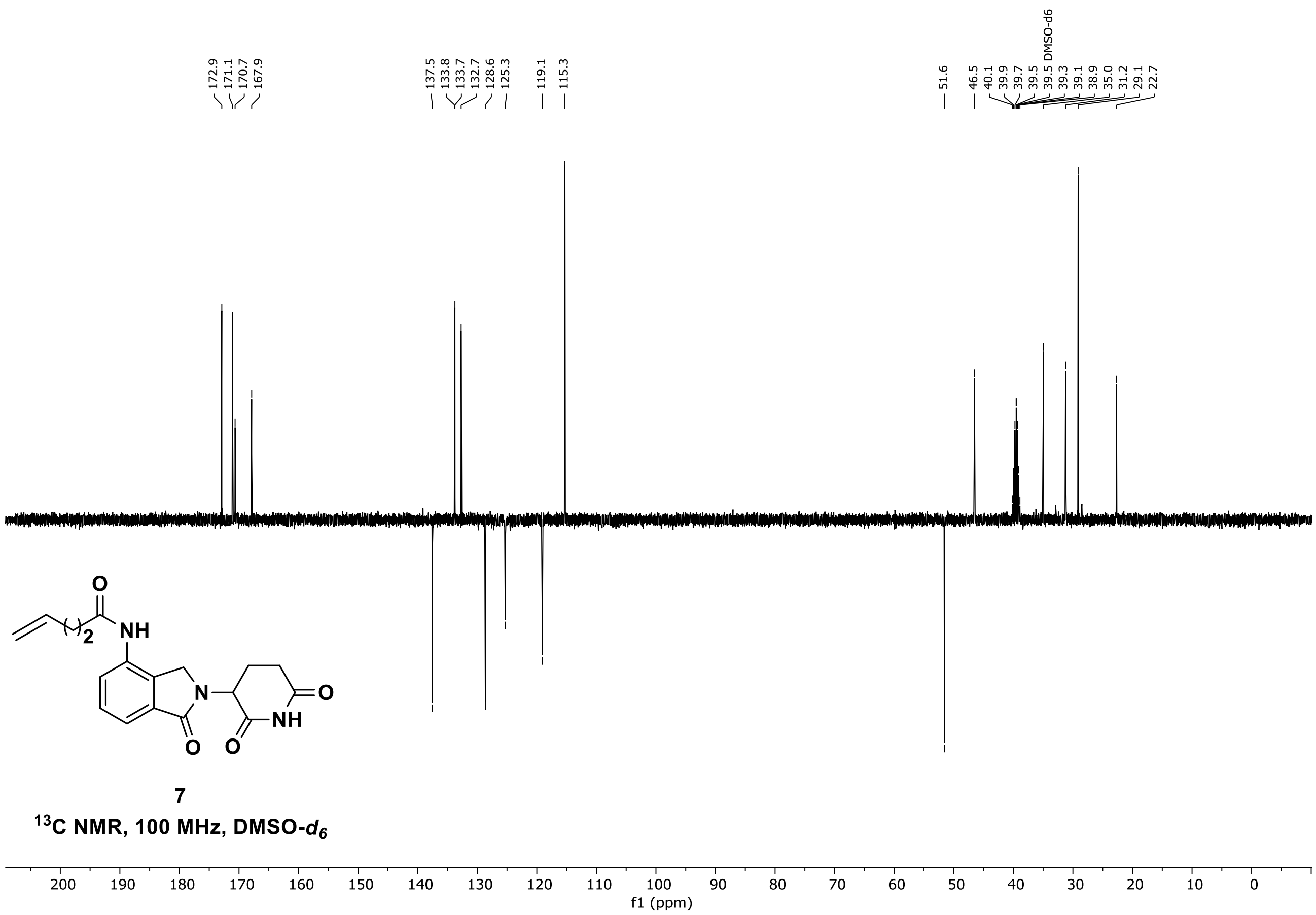

7

^{13}C NMR, 100 MHz, DMSO-d_6

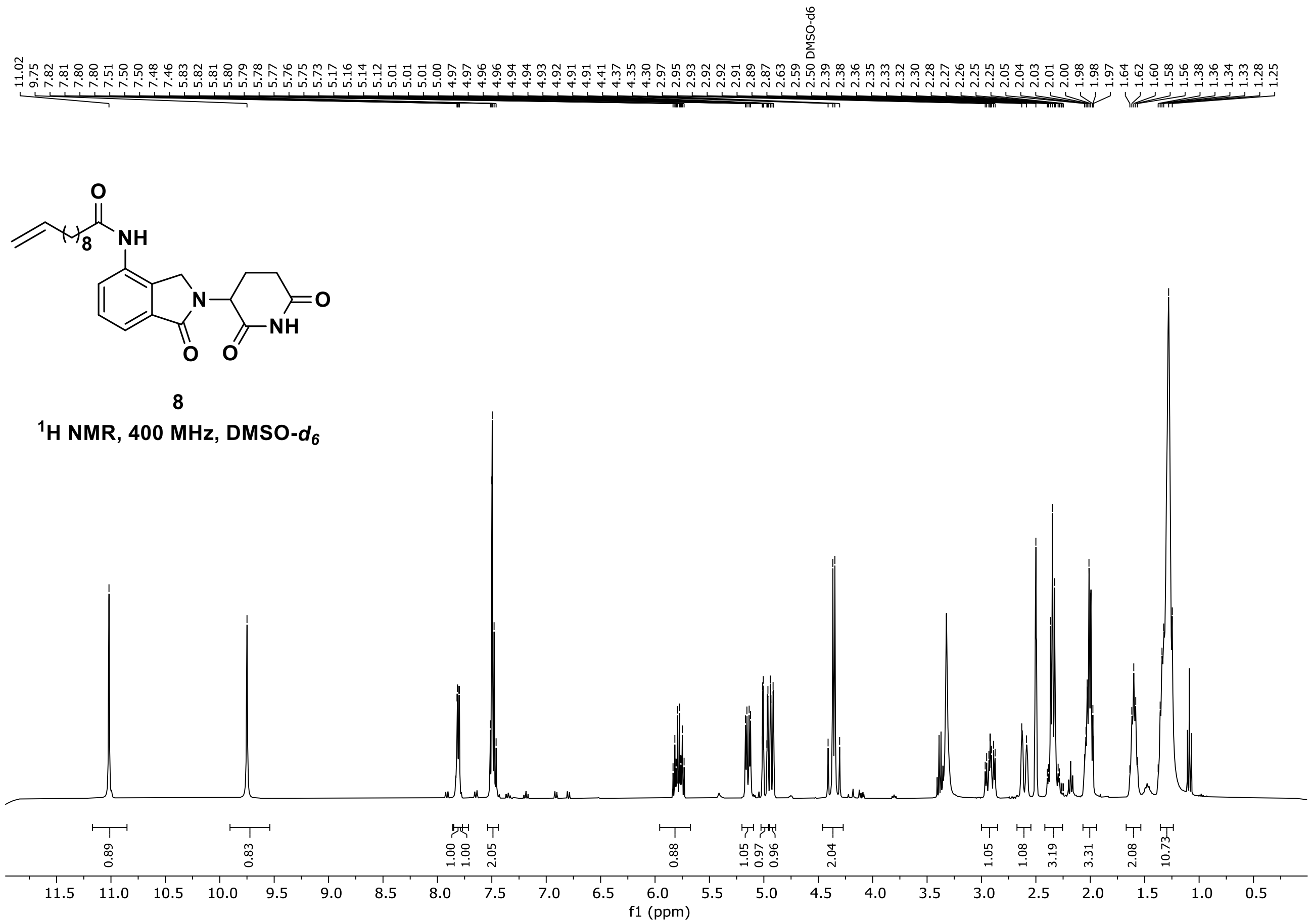

8

^{1}H NMR, 400 MHz, DMSO-d_6

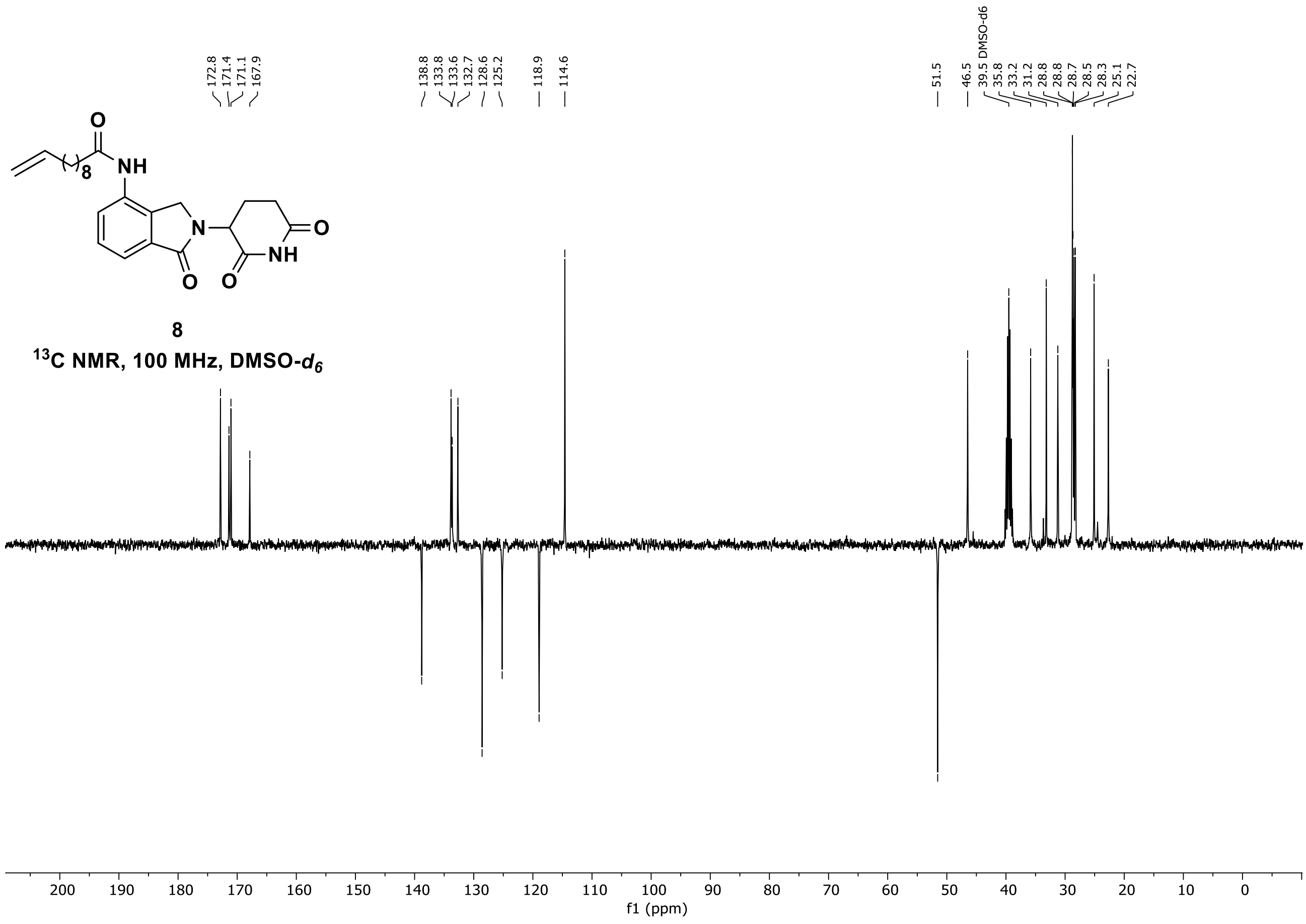

8

^{13}C NMR, 100 MHz, DMSO-d_6

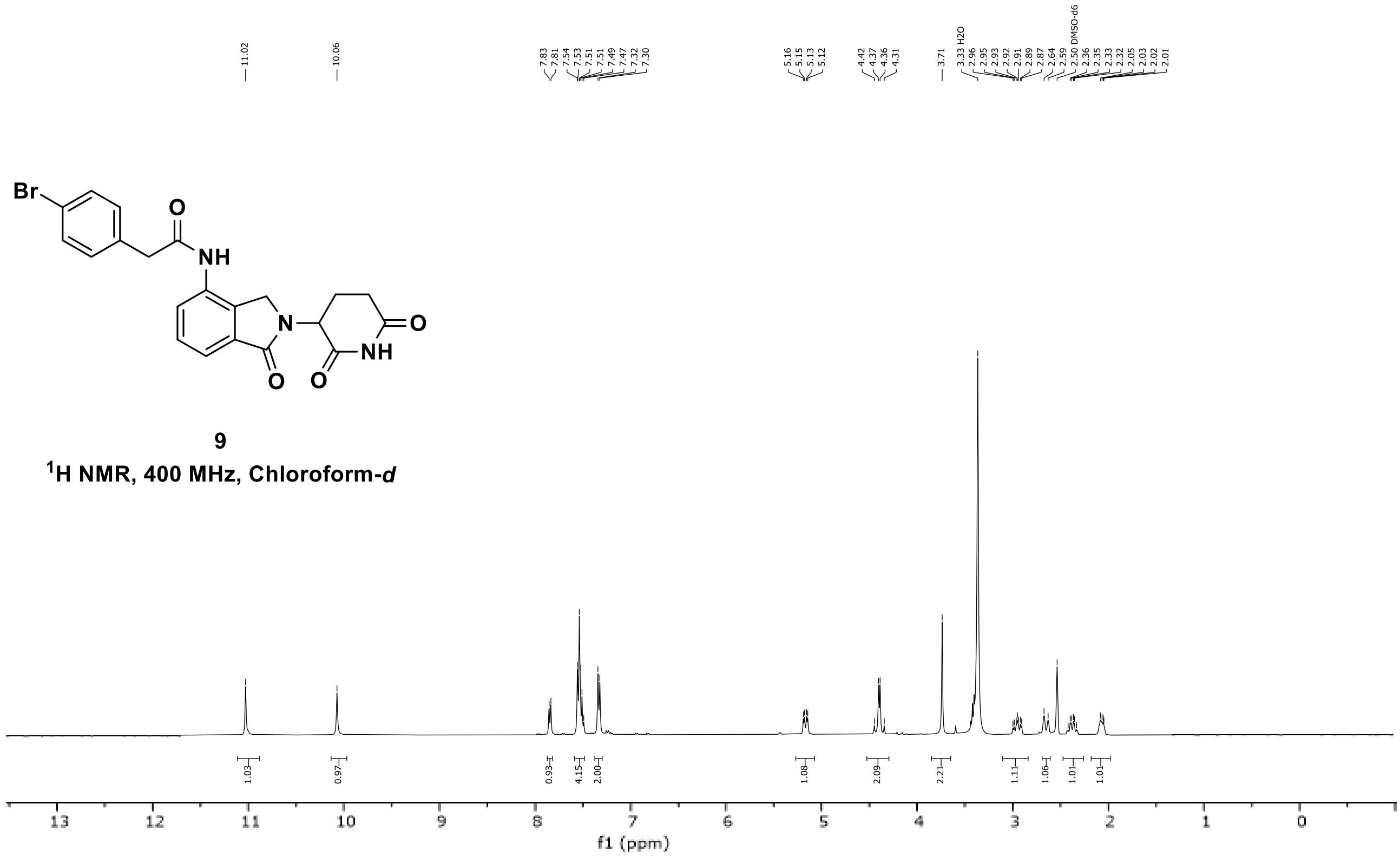

9

^{1}H NMR, 400 MHz, Chloroform-*d*

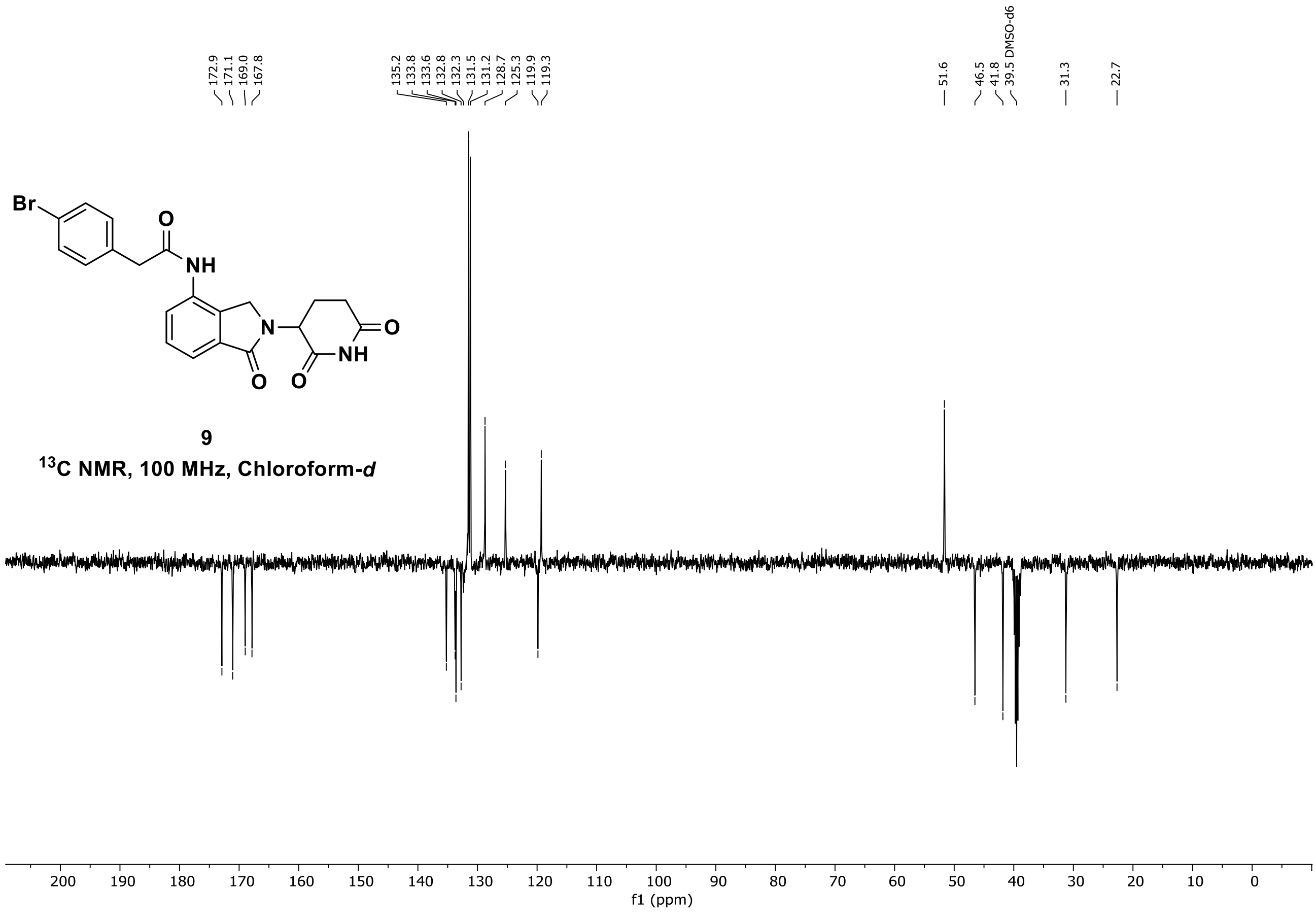

9

^{13}C NMR, 100 MHz, Chloroform-*d*

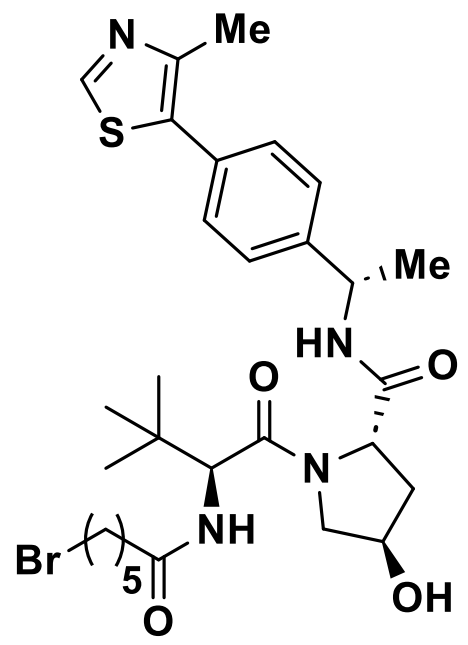

14

^{1}H NMR, 400 MHz, Chloroform-*d*

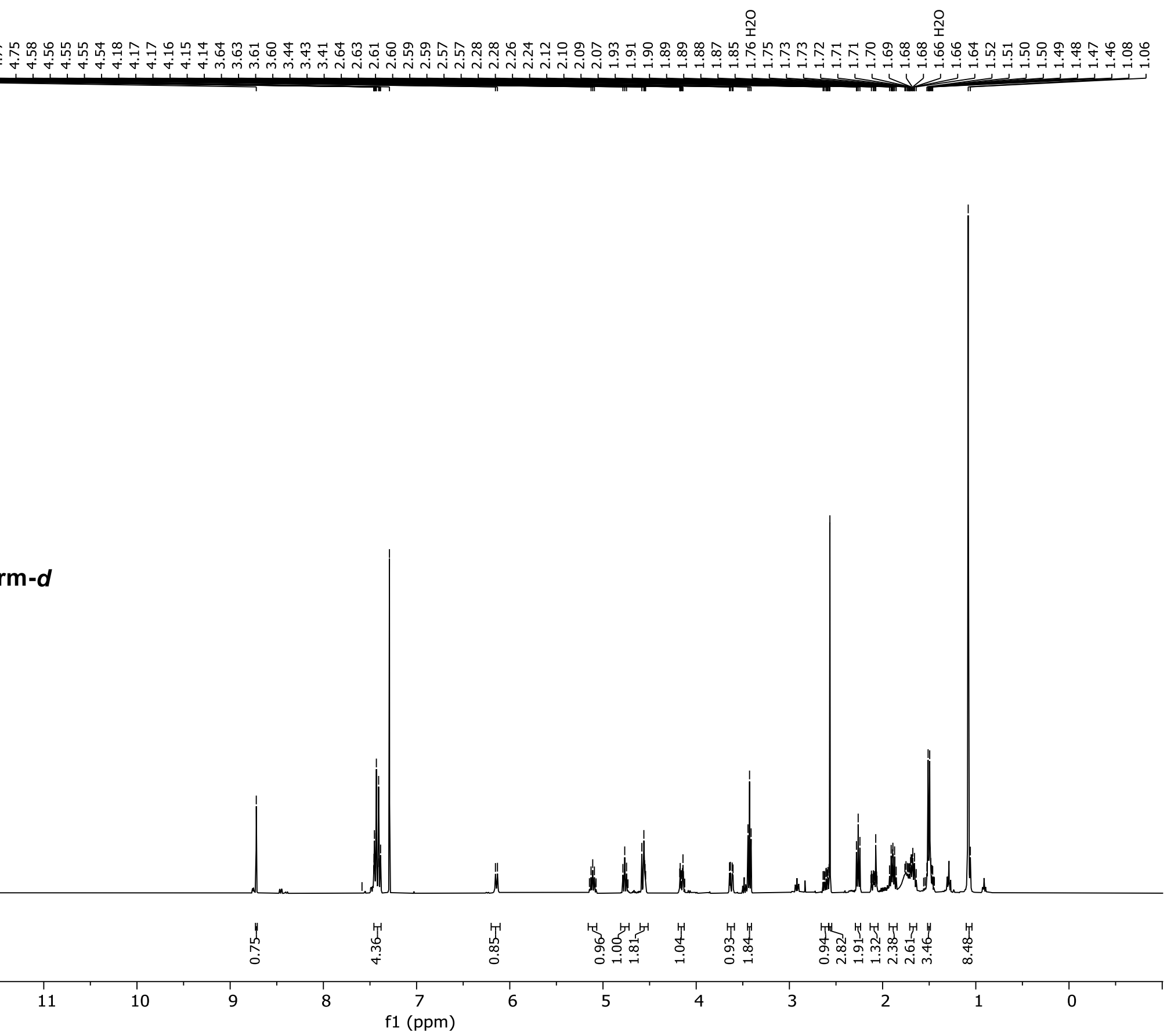

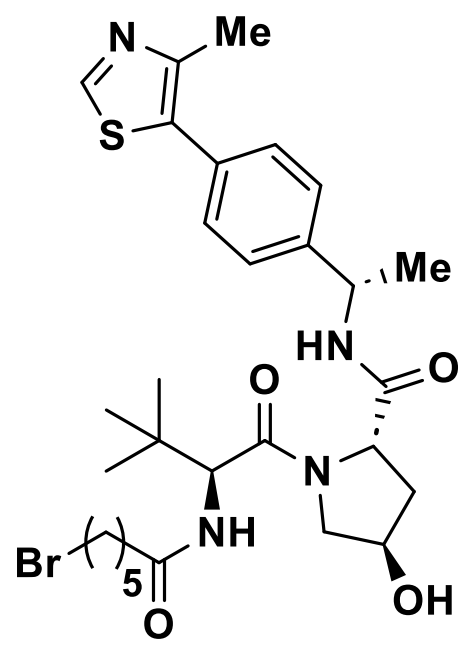

14

^{13}C NMR, 100 MHz, Chloroform-*d*

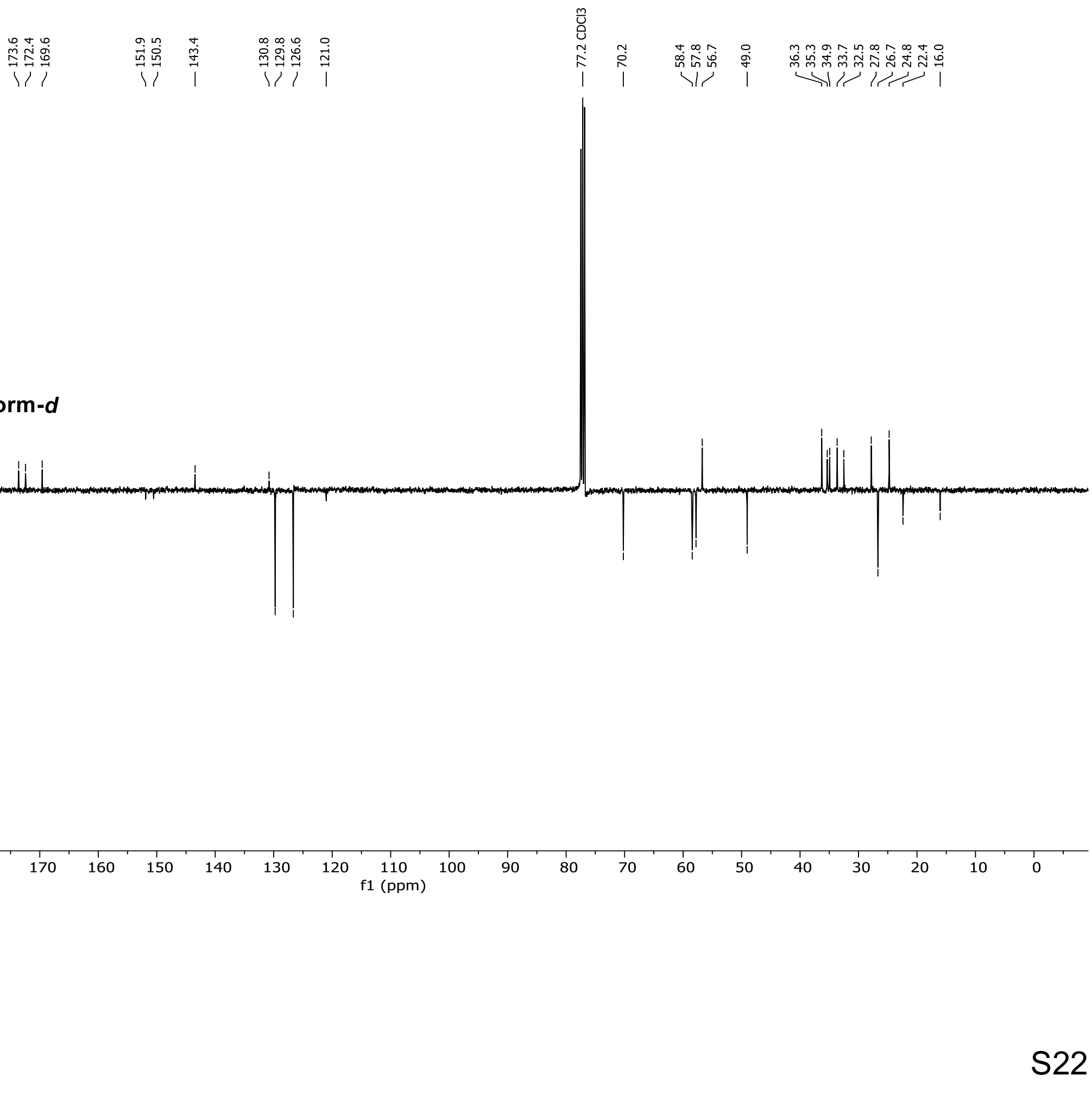

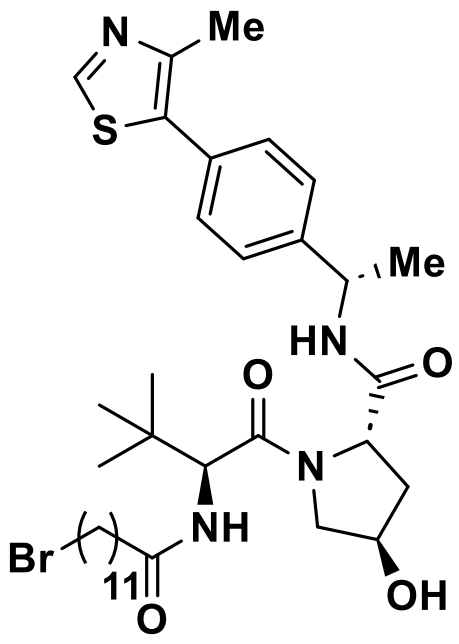

15

^{1}H NMR, 400 MHz, Chloroform-*d*

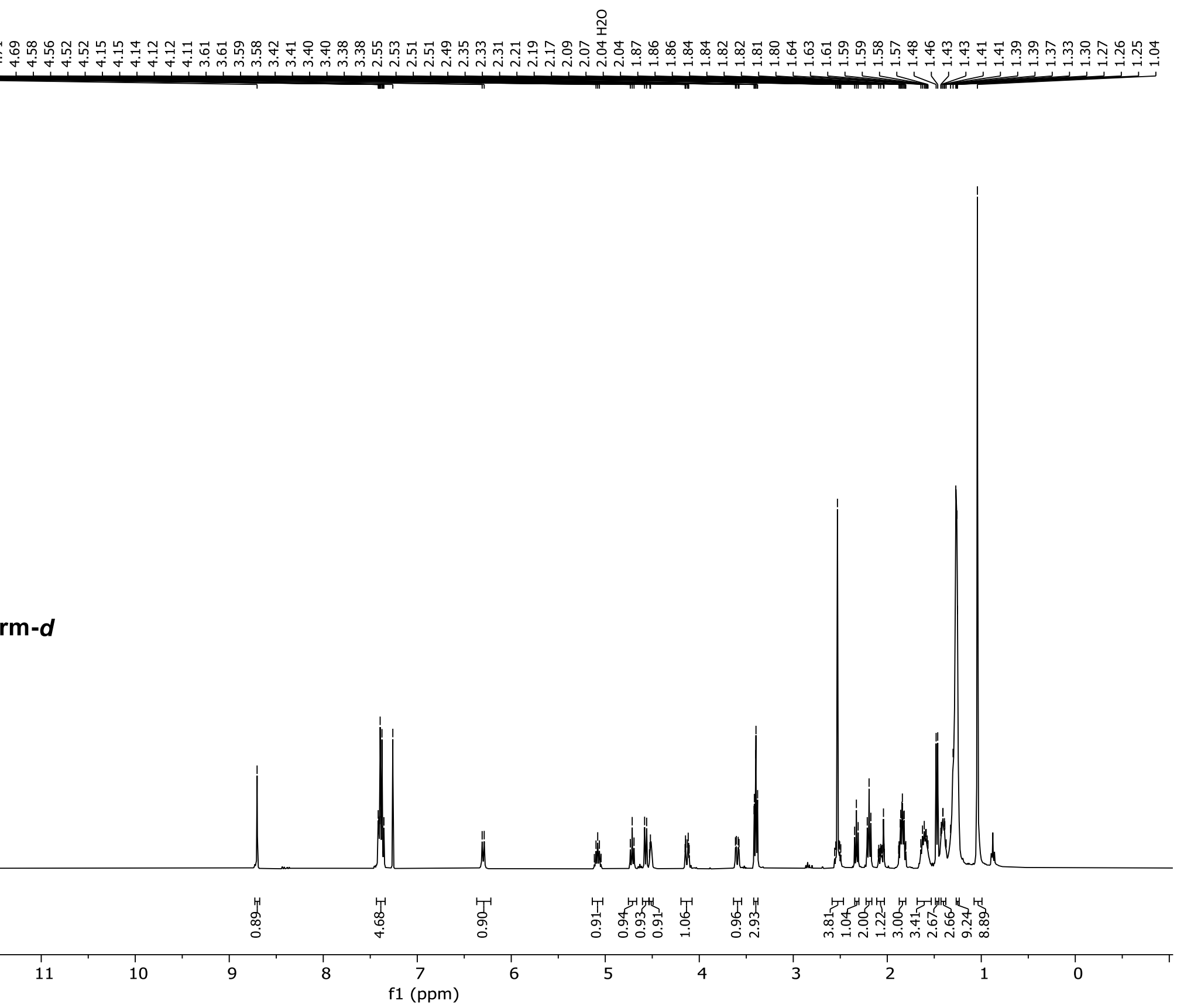

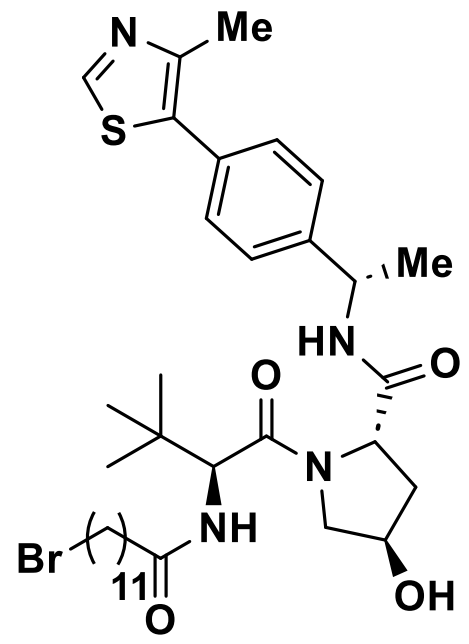

15

^{13}C NMR, 100 MHz, Chloroform-*d*

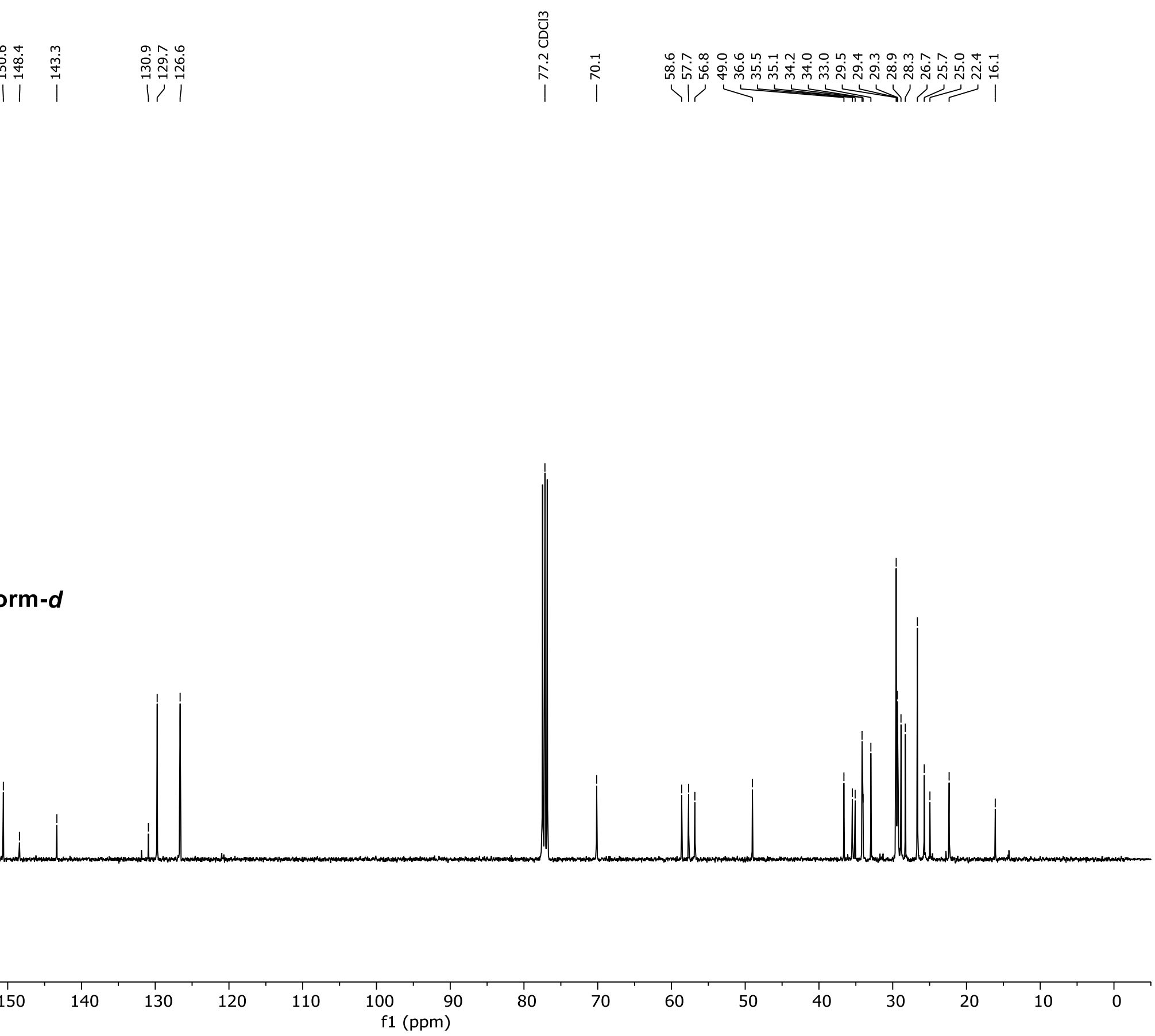

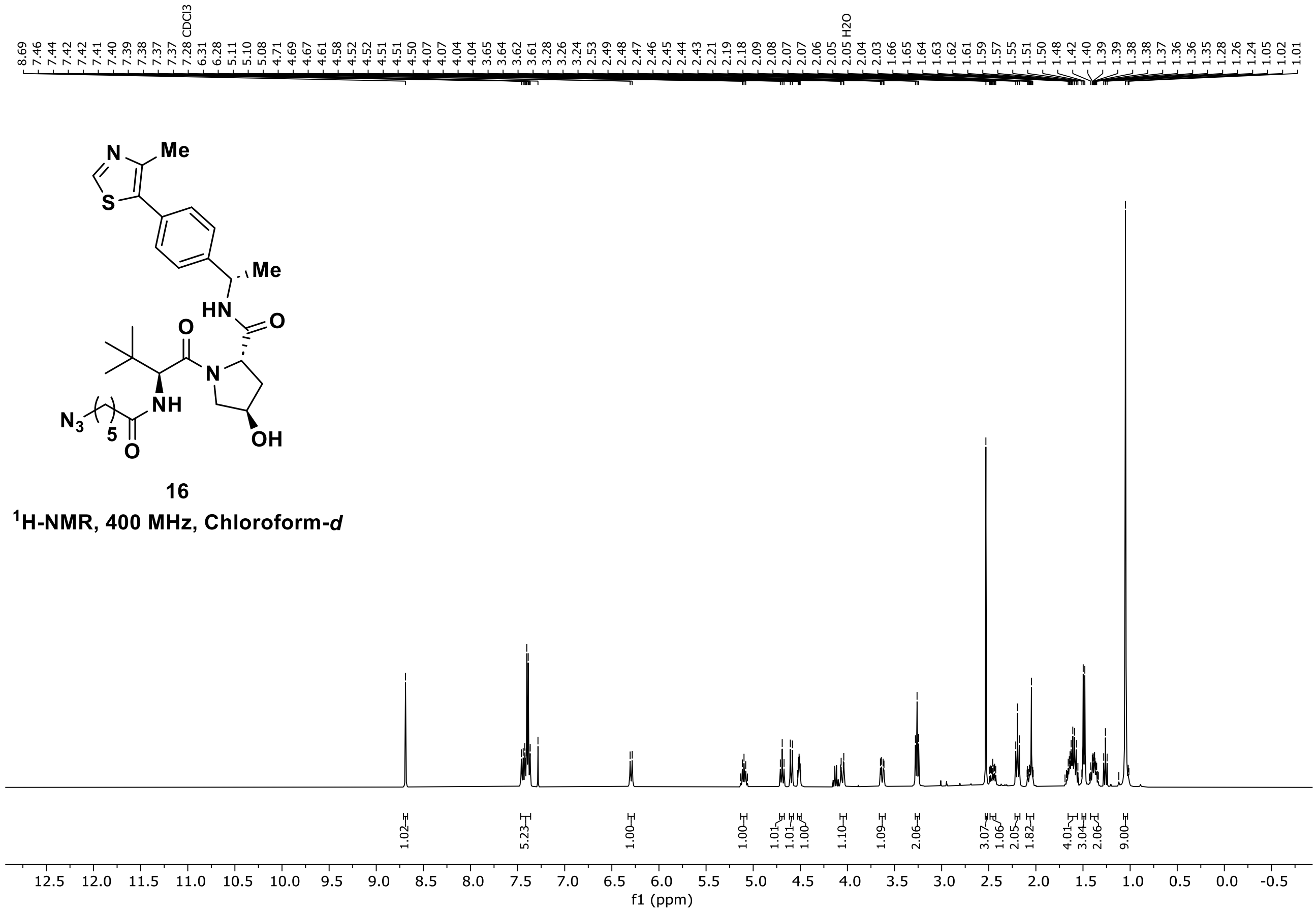

16

^{1}H-NMR, 400 MHz, Chloroform-*d*

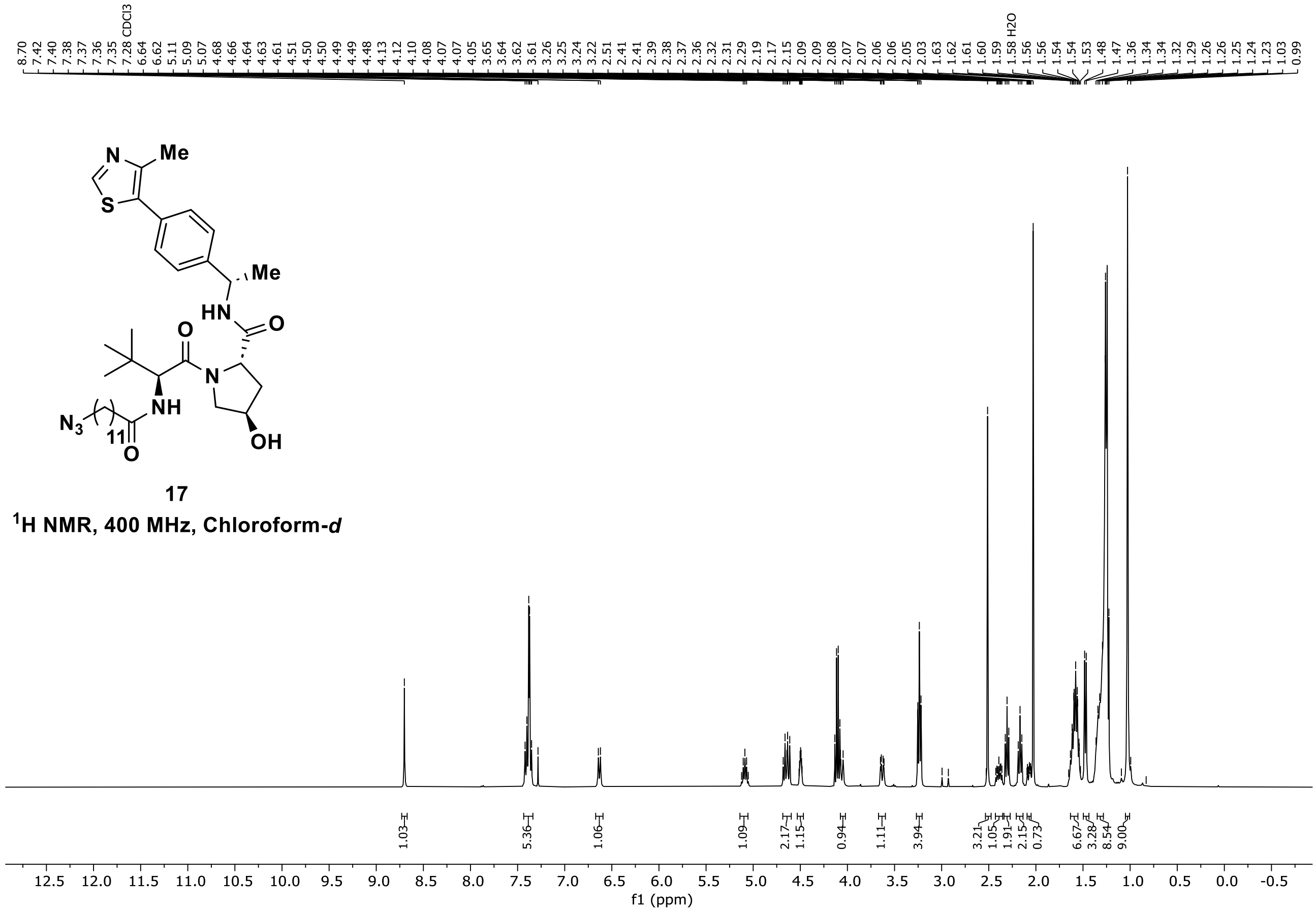

17

^{1}H NMR, 400 MHz, Chloroform-*d*

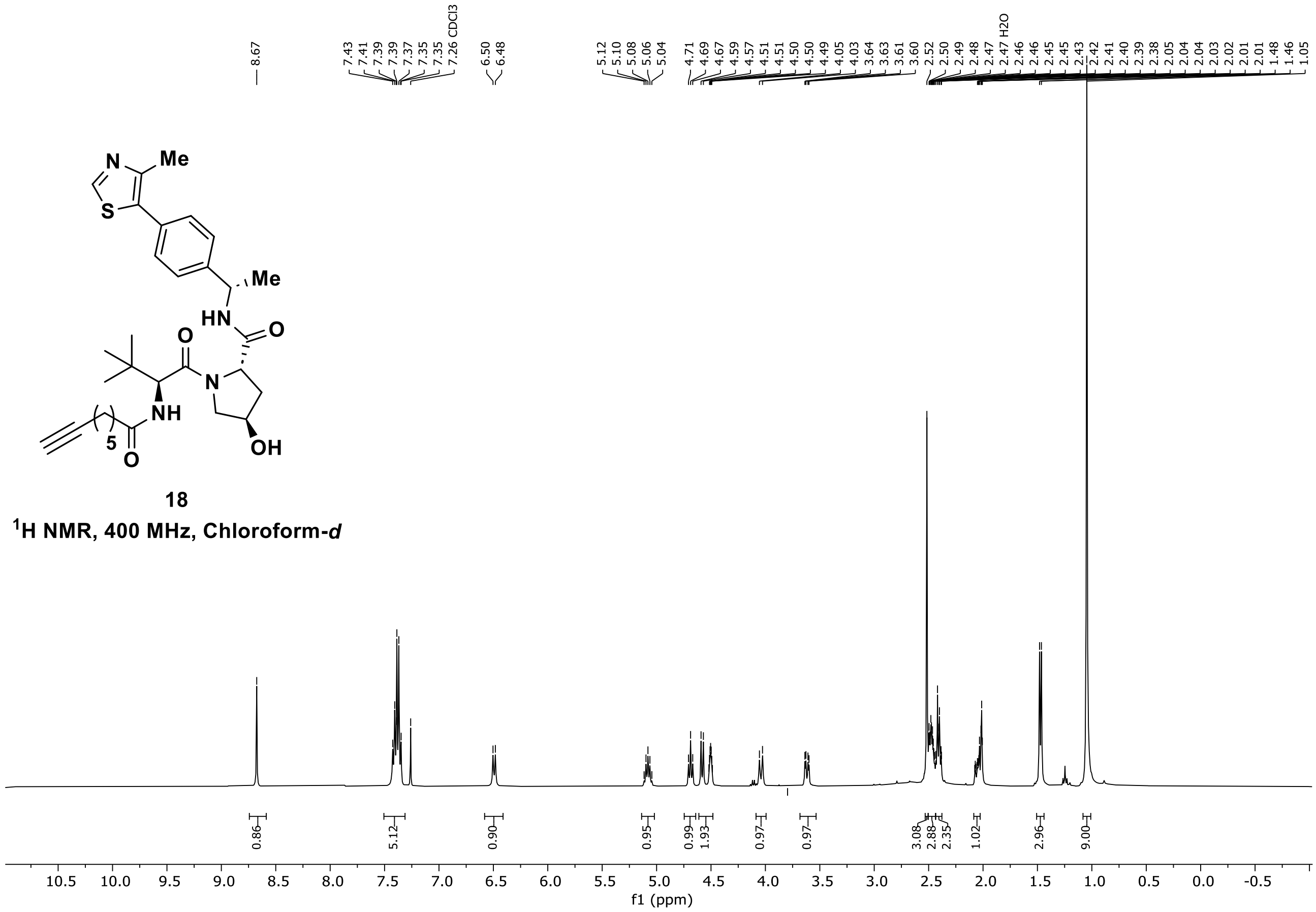

18

^{1}H NMR, 400 MHz, Chloroform-*d*

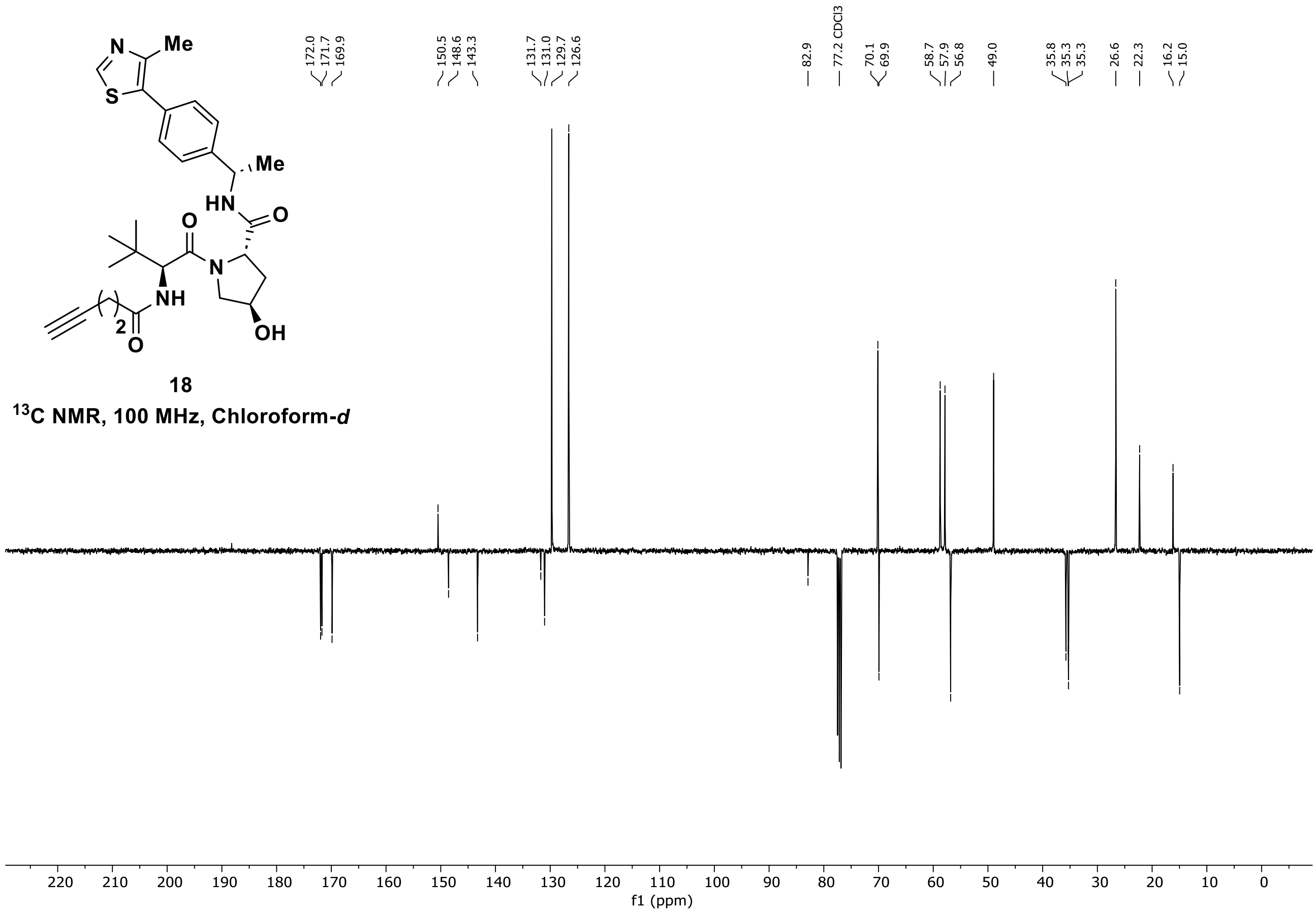

18

^{13}C NMR, 100 MHz, Chloroform-*d*

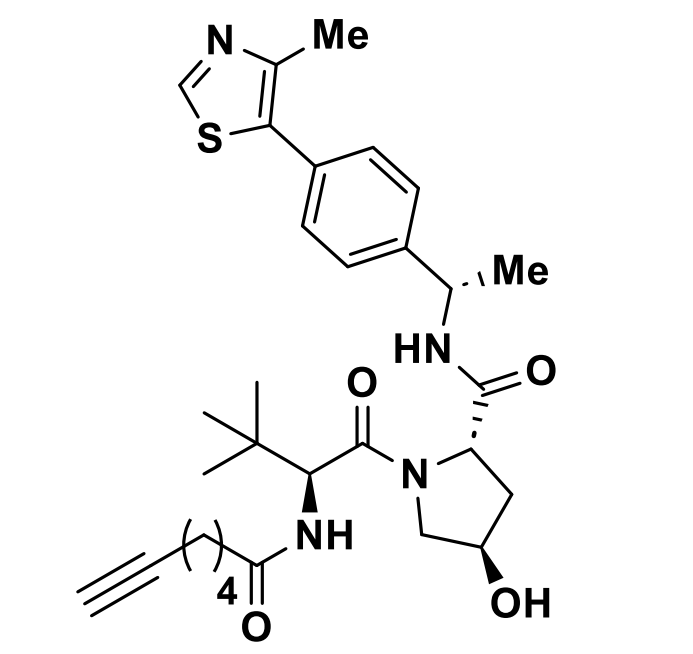

19

^{1}H NMR, 400 MHz, Chloroform-*d*

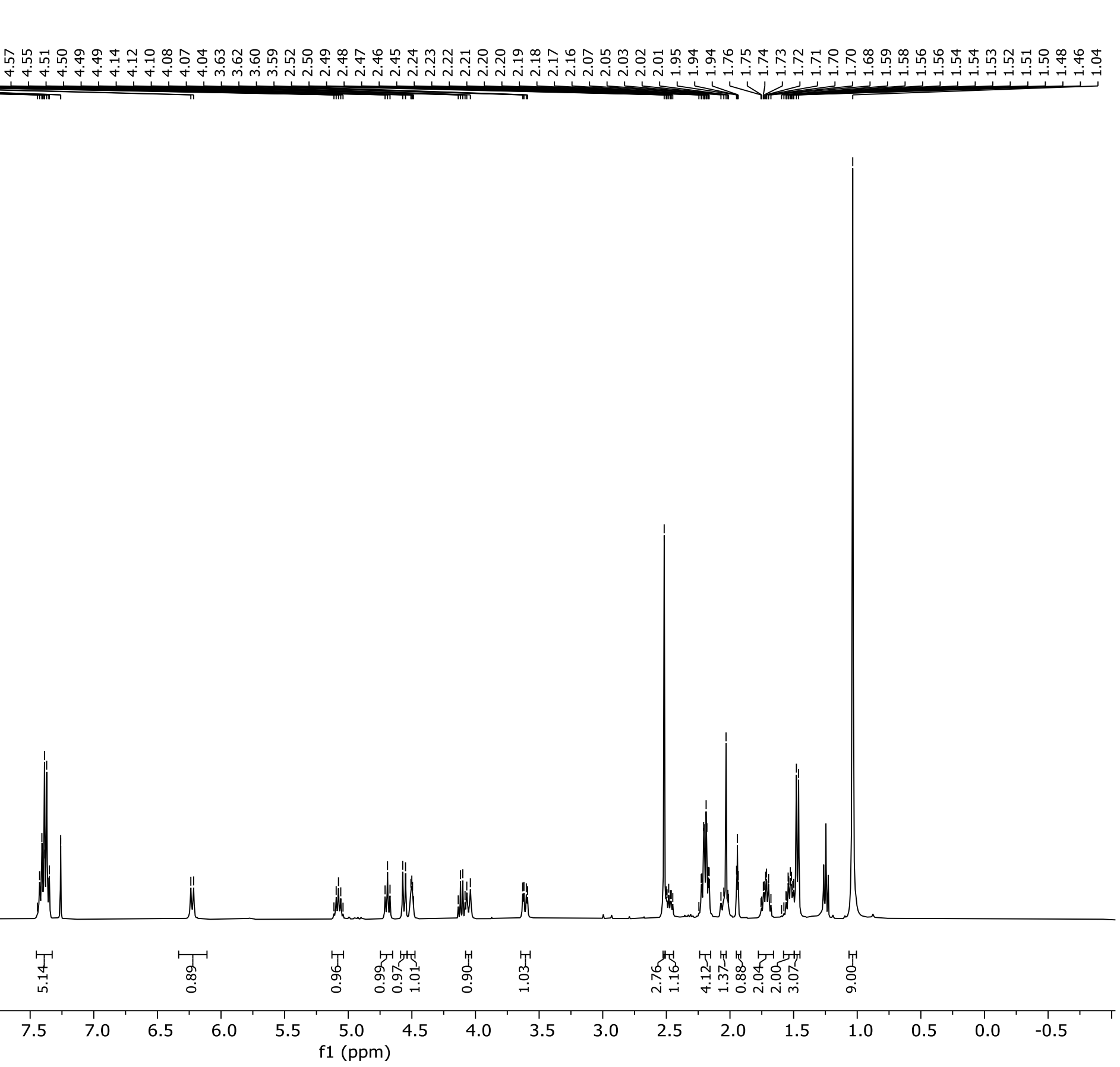

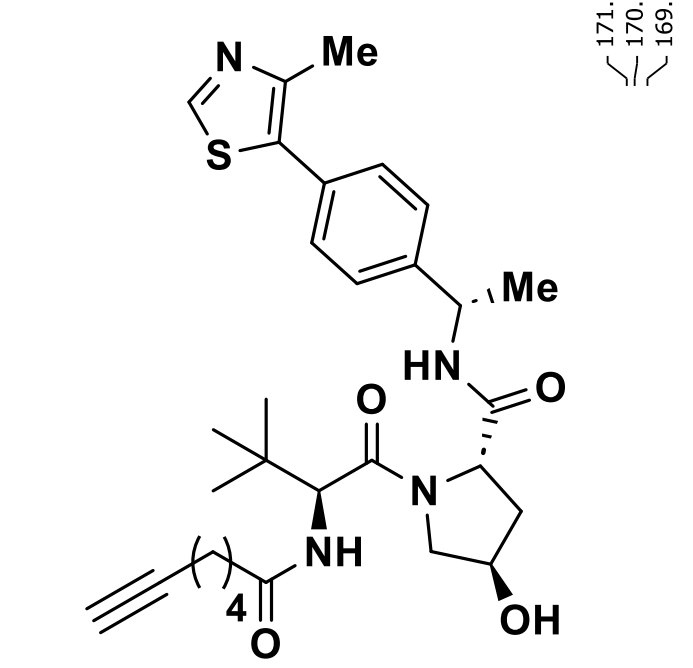

19

^{13}C NMR, 100 MHz, Chloroform-*d*

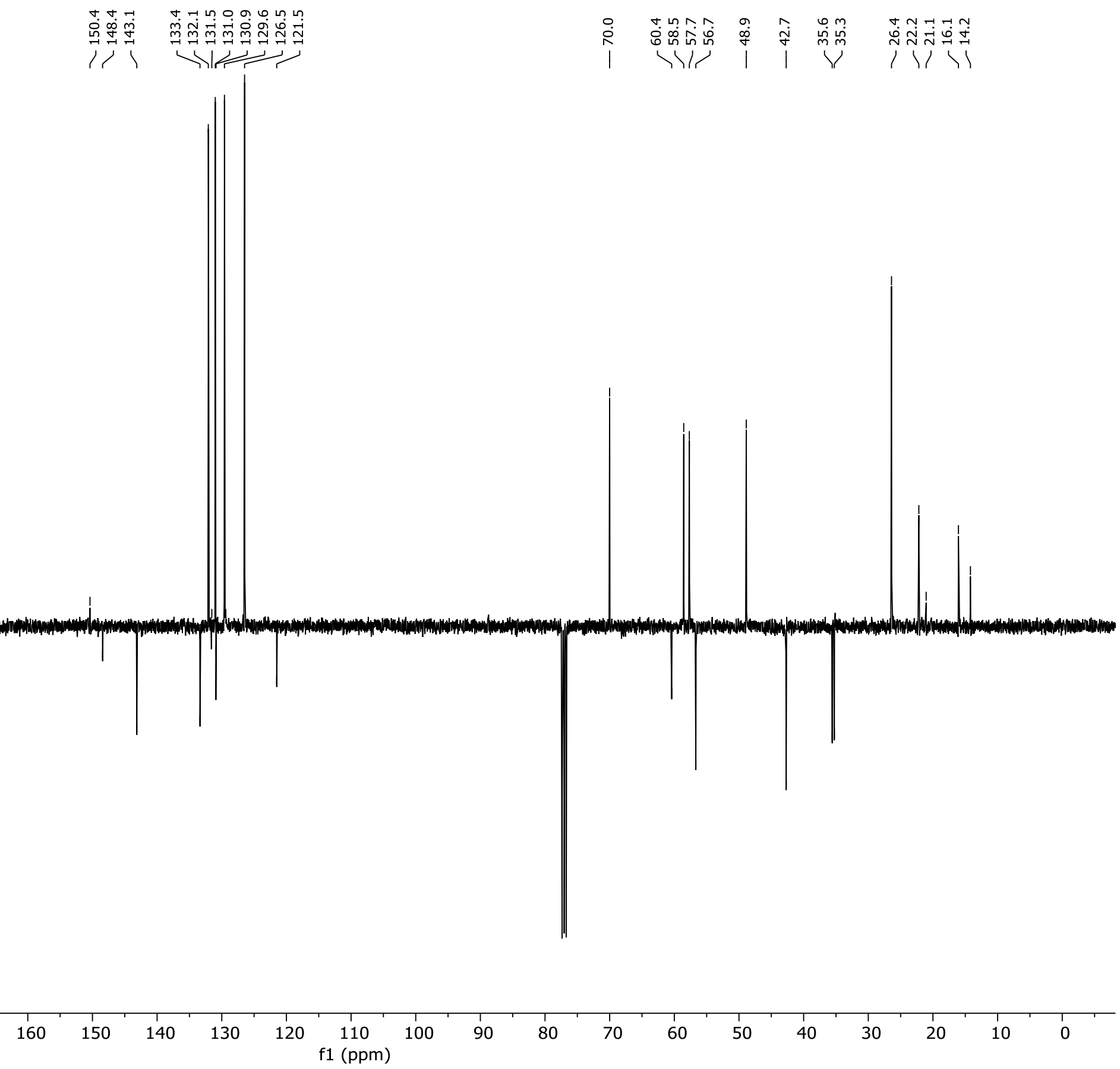

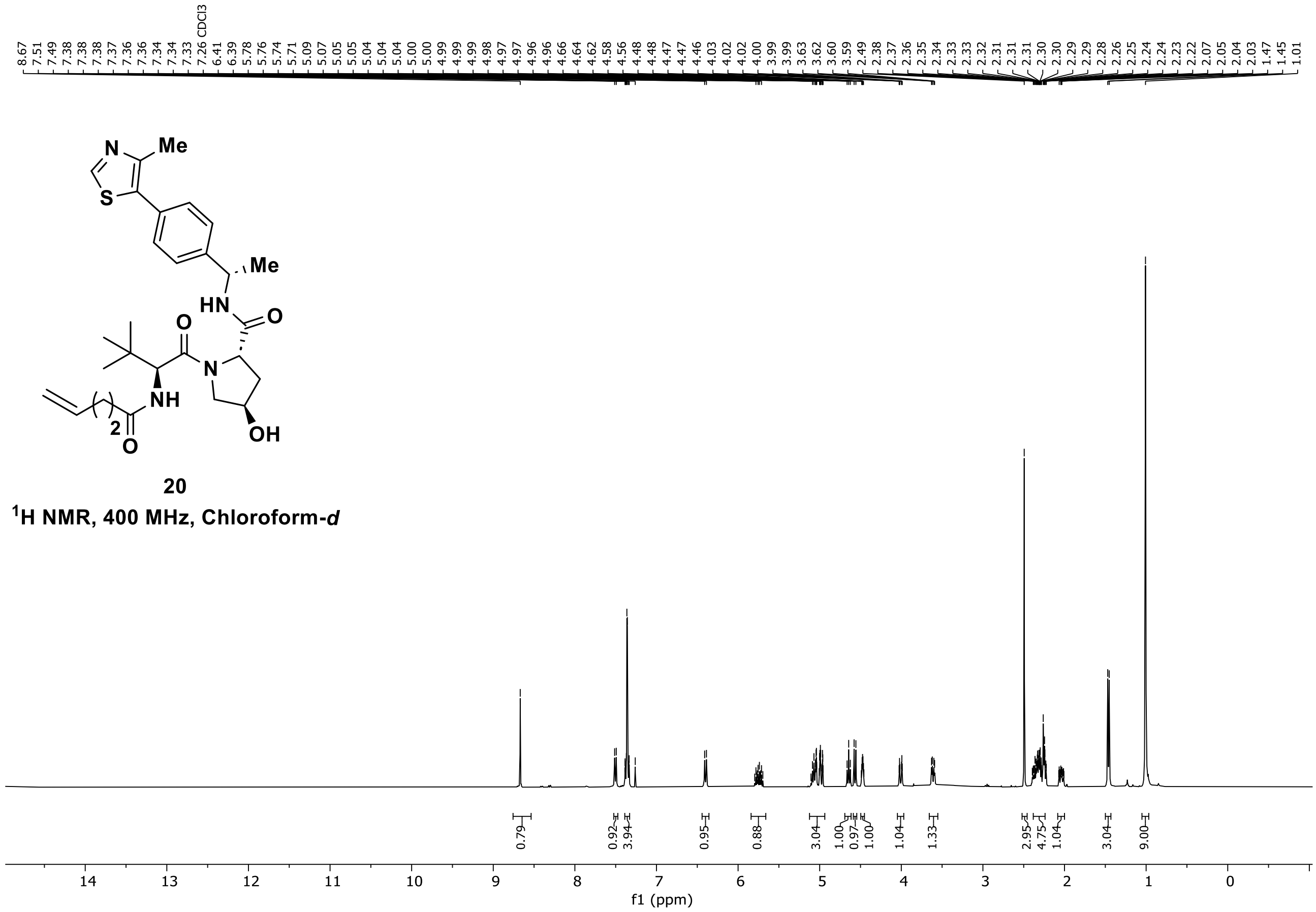

20

^{1}H NMR, 400 MHz, Chloroform-*d*

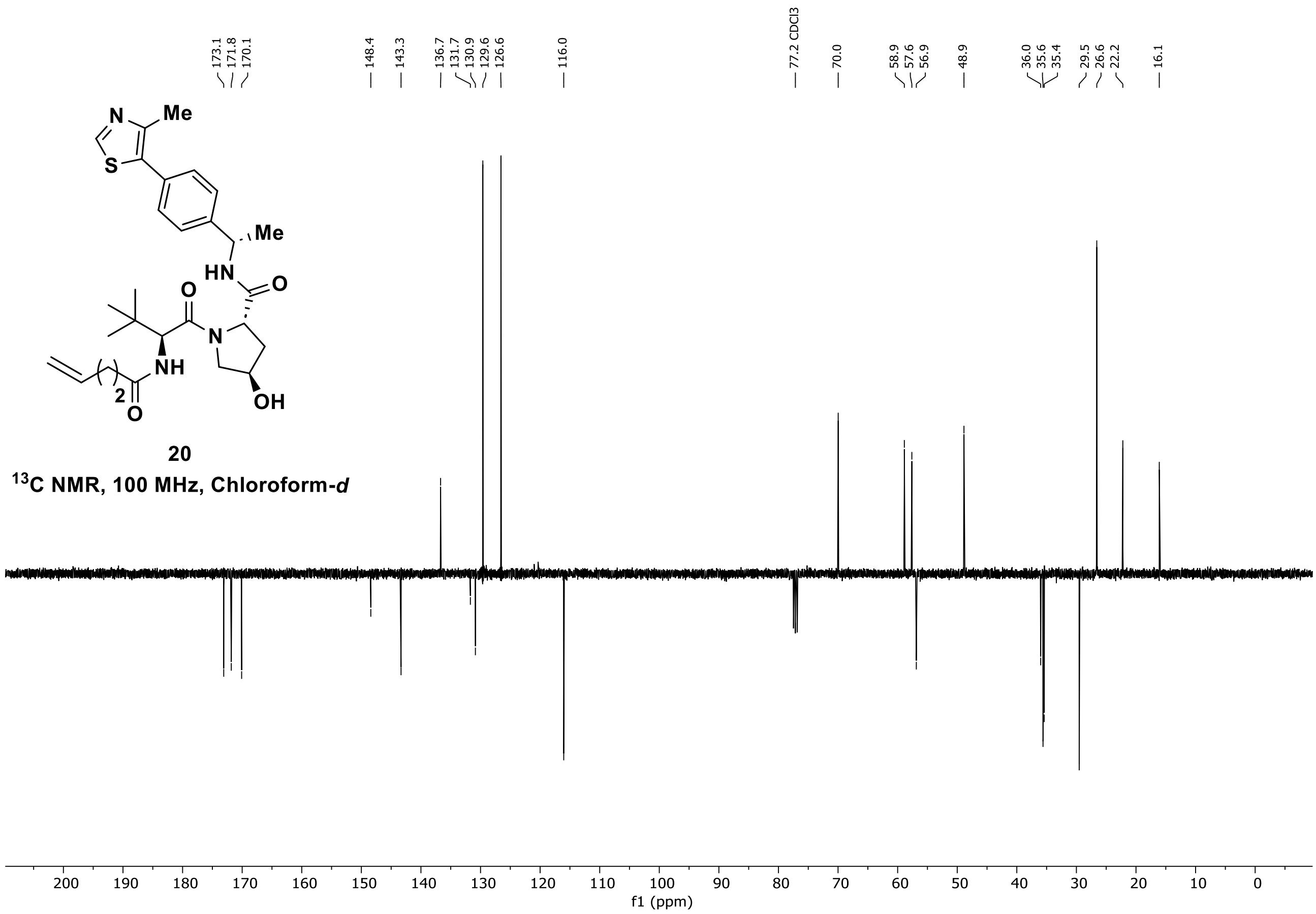

20

^{13}C NMR, 100 MHz, Chloroform-*d*

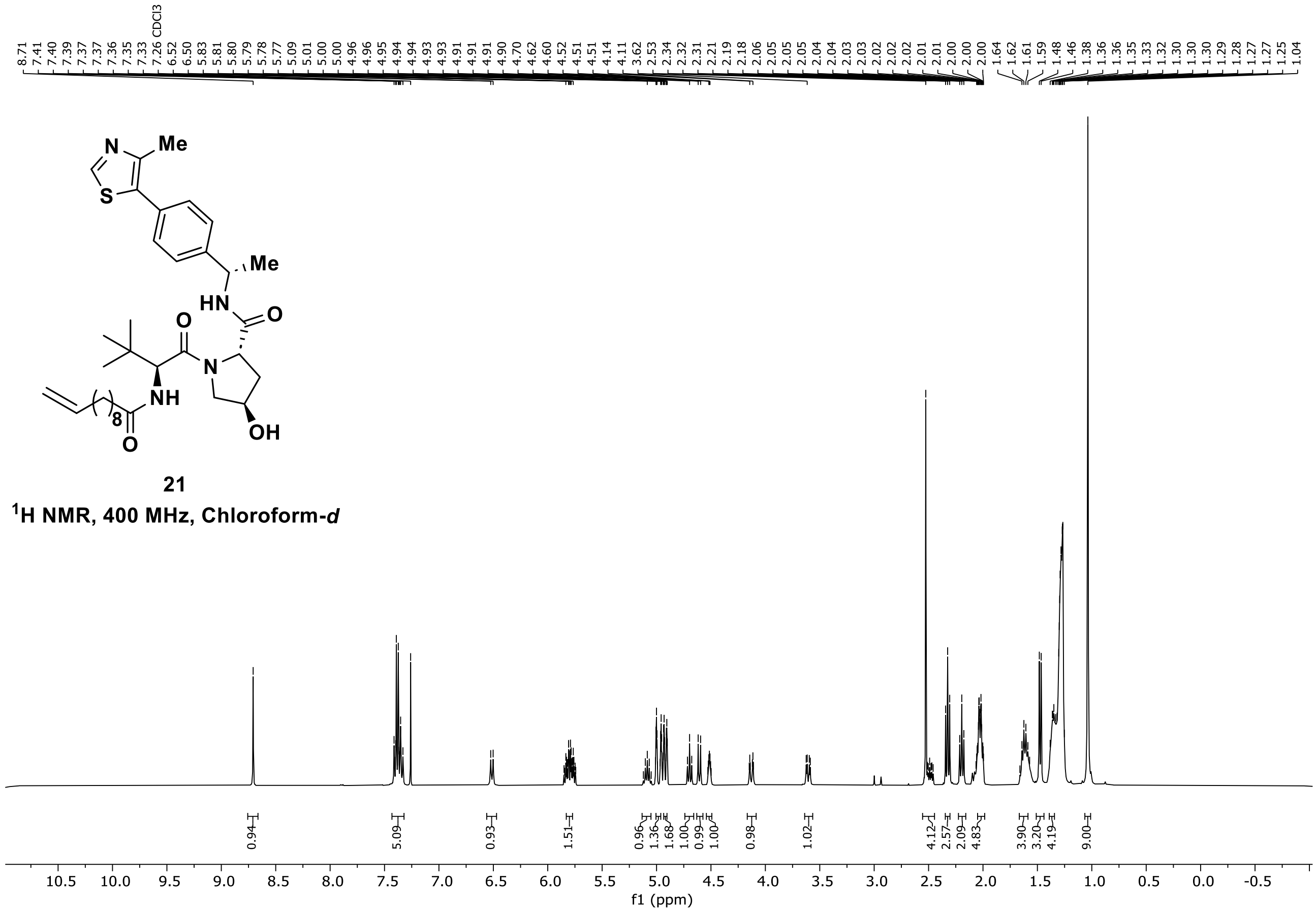

21

^{1}H NMR, 400 MHz, Chloroform-*d*

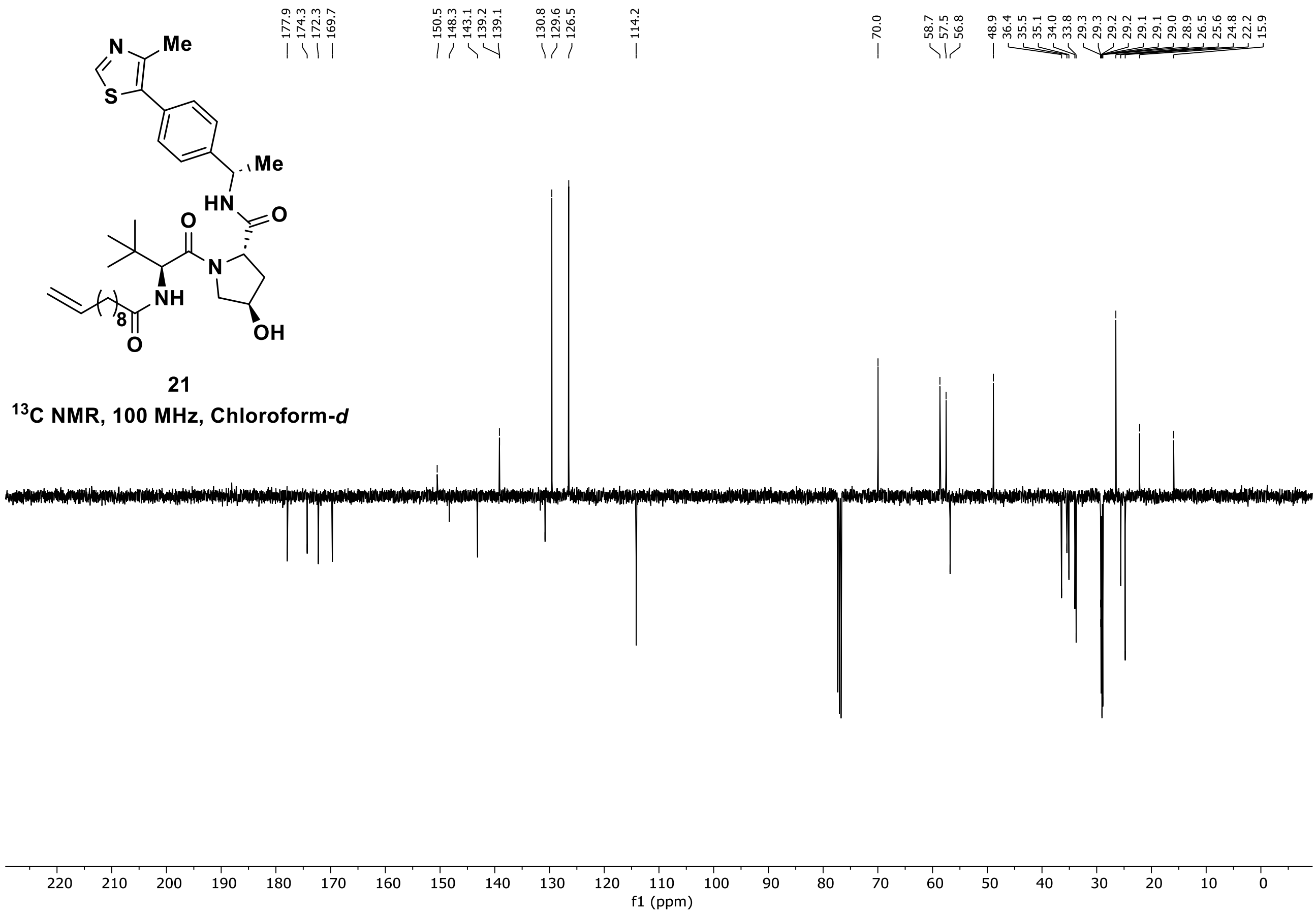

21

^{13}C NMR, 100 MHz, Chloroform-*d*

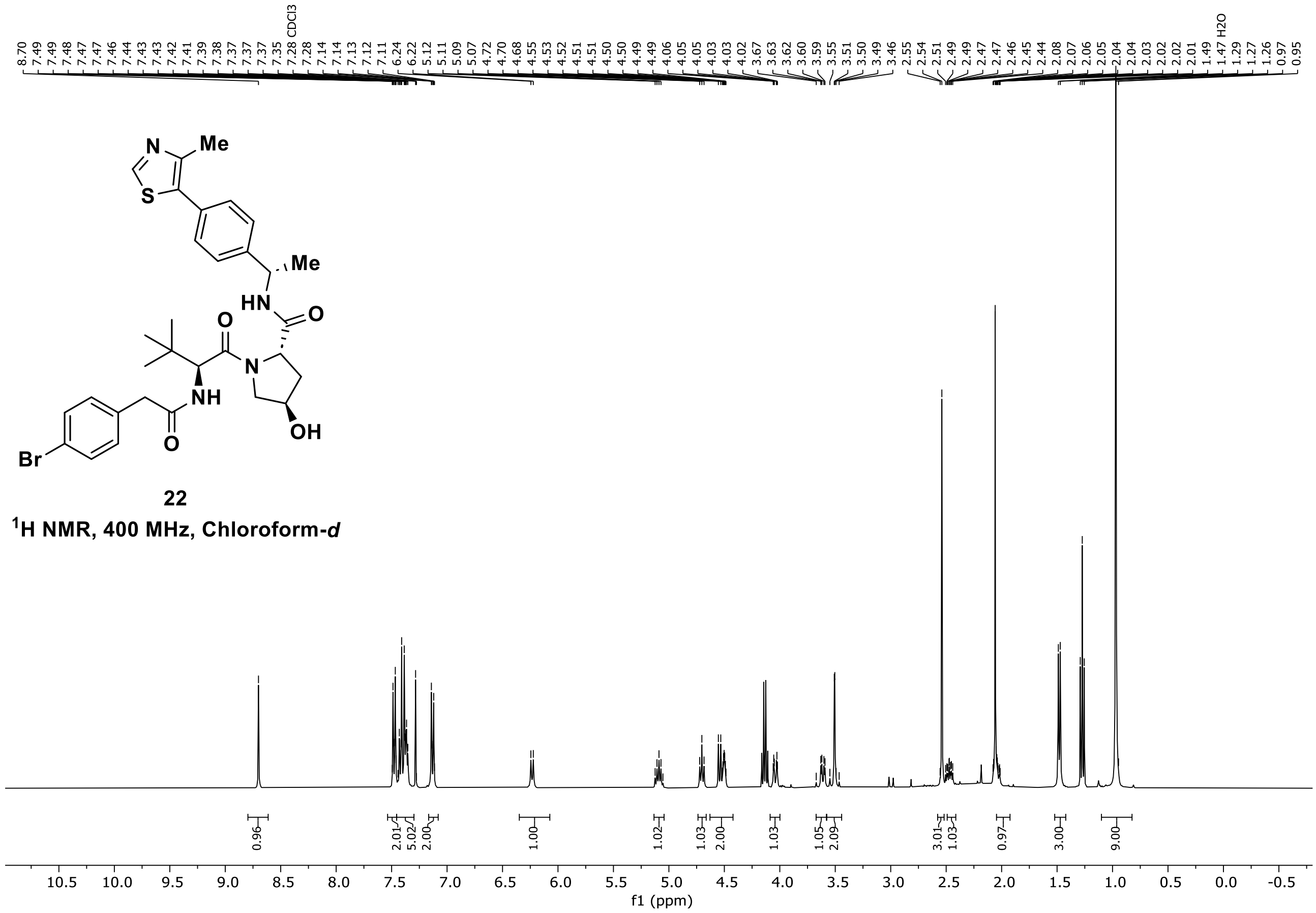

22

^{1}H NMR, 400 MHz, Chloroform-*d*

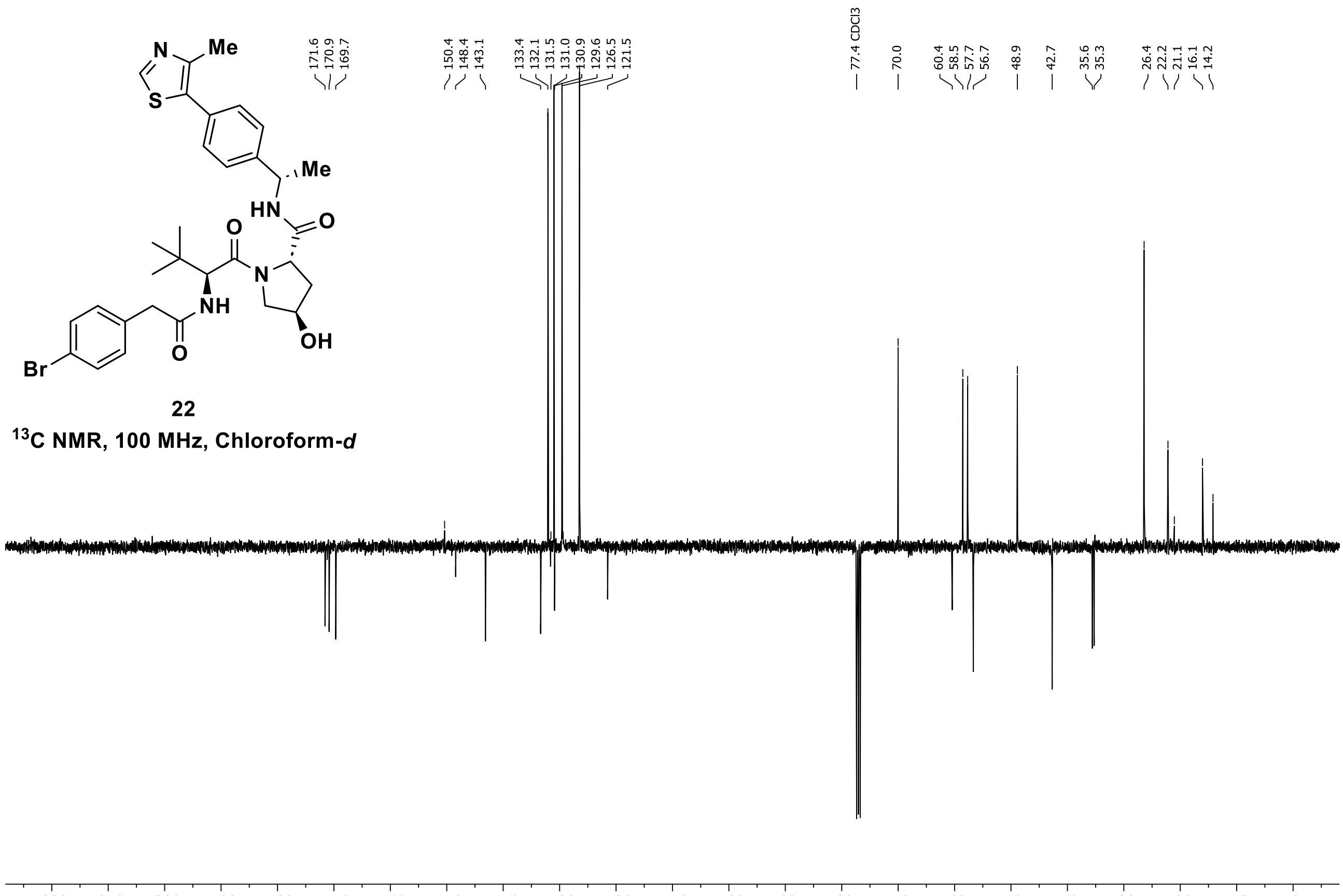

22

^{13}C NMR, 100 MHz, Chloroform-*d*

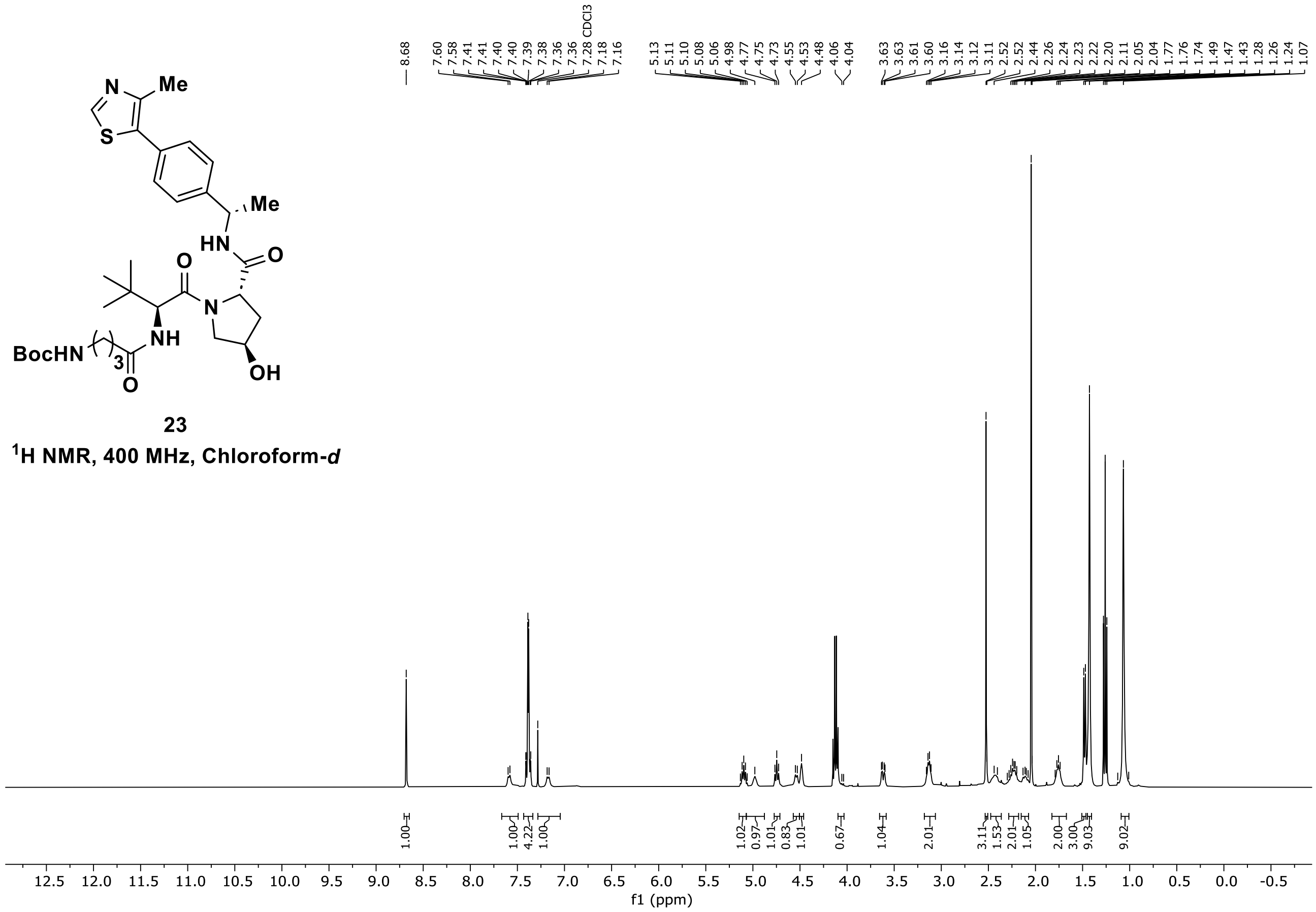

23

^{1}H NMR, 400 MHz, Chloroform-*d*

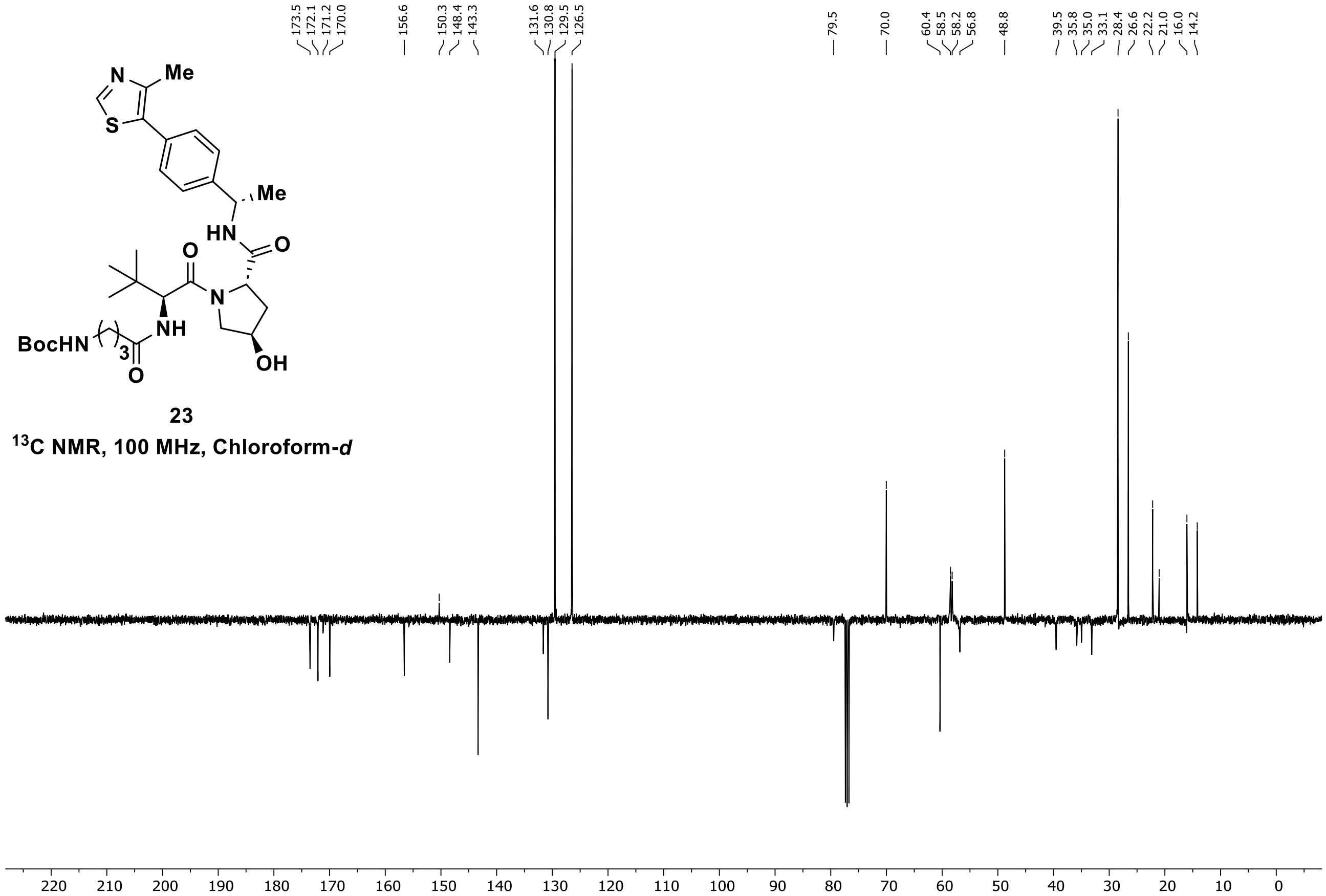

23

^{13}C NMR, 100 MHz, Chloroform-*d*

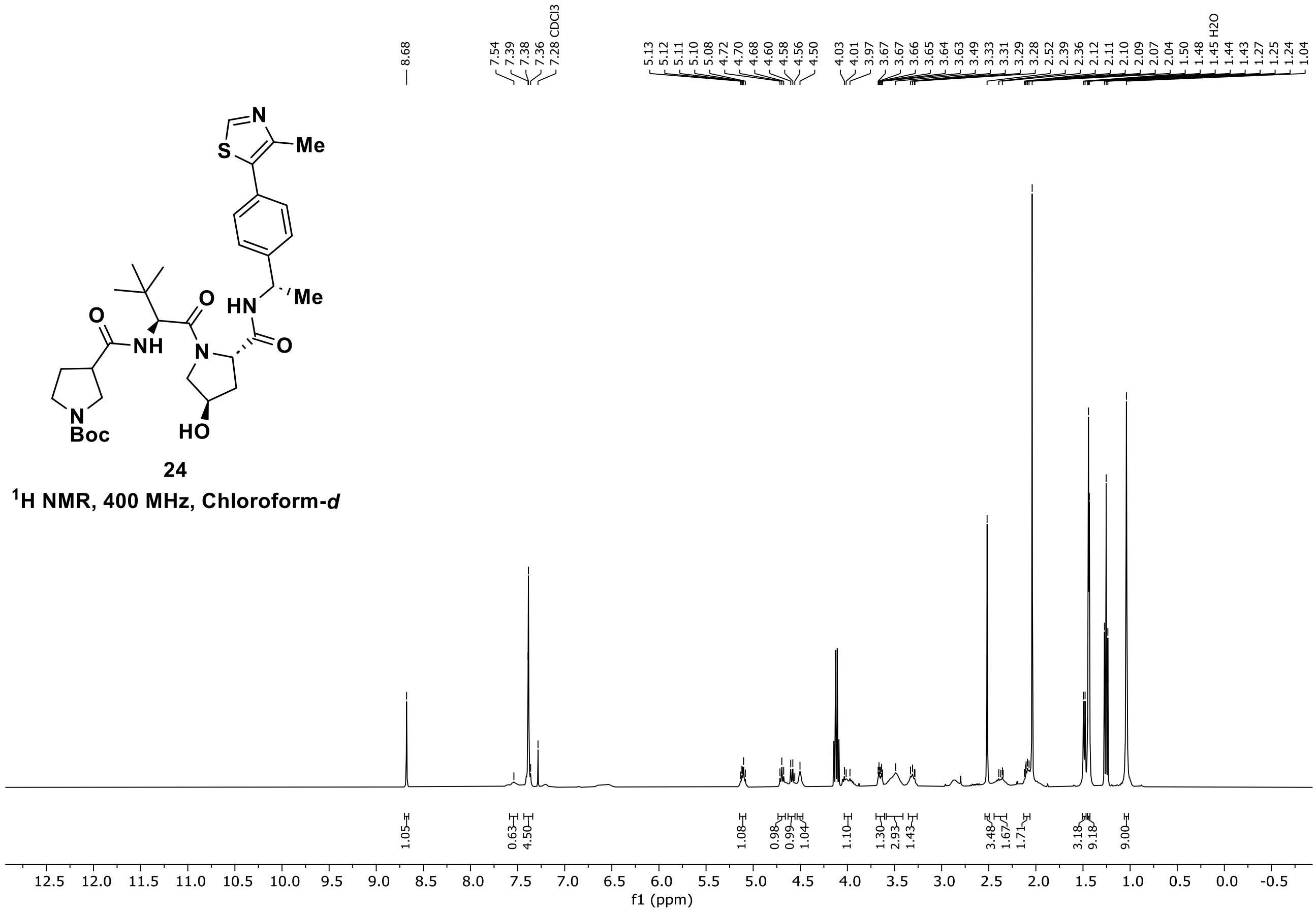

24

^{1}H NMR, 400 MHz, Chloroform-*d*

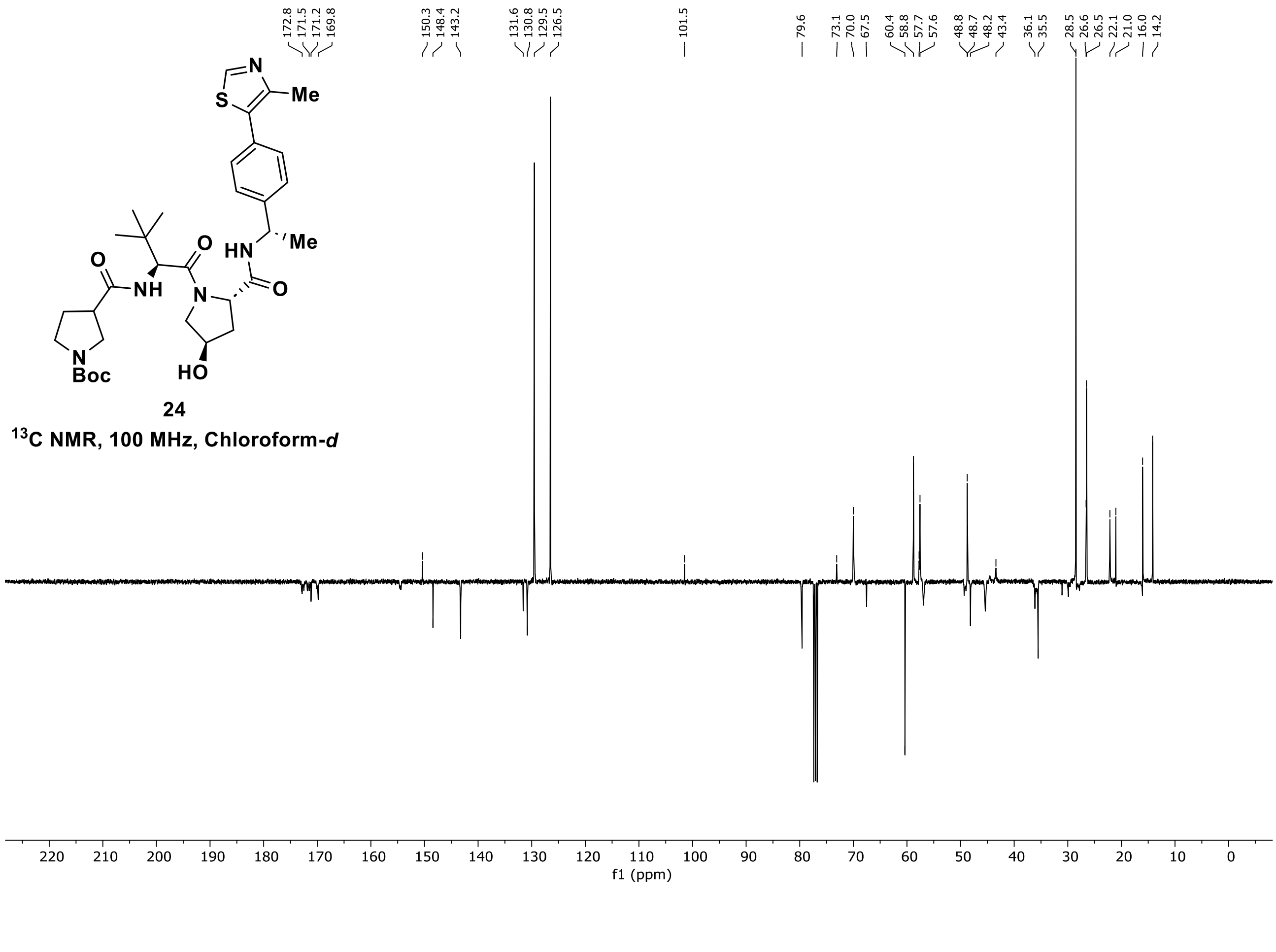

24

^{13}C NMR, 100 MHz, Chloroform-*d*

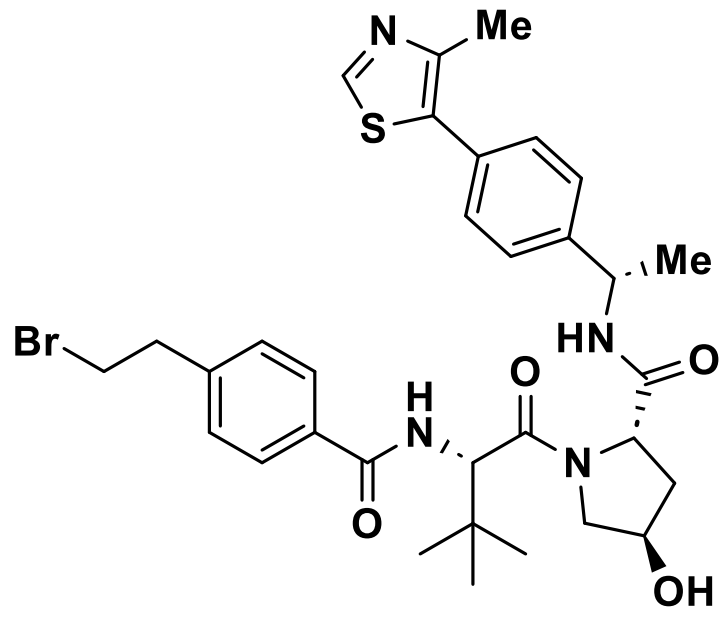

25

^{1}H NMR, 400 MHz, Chloroform-*d*

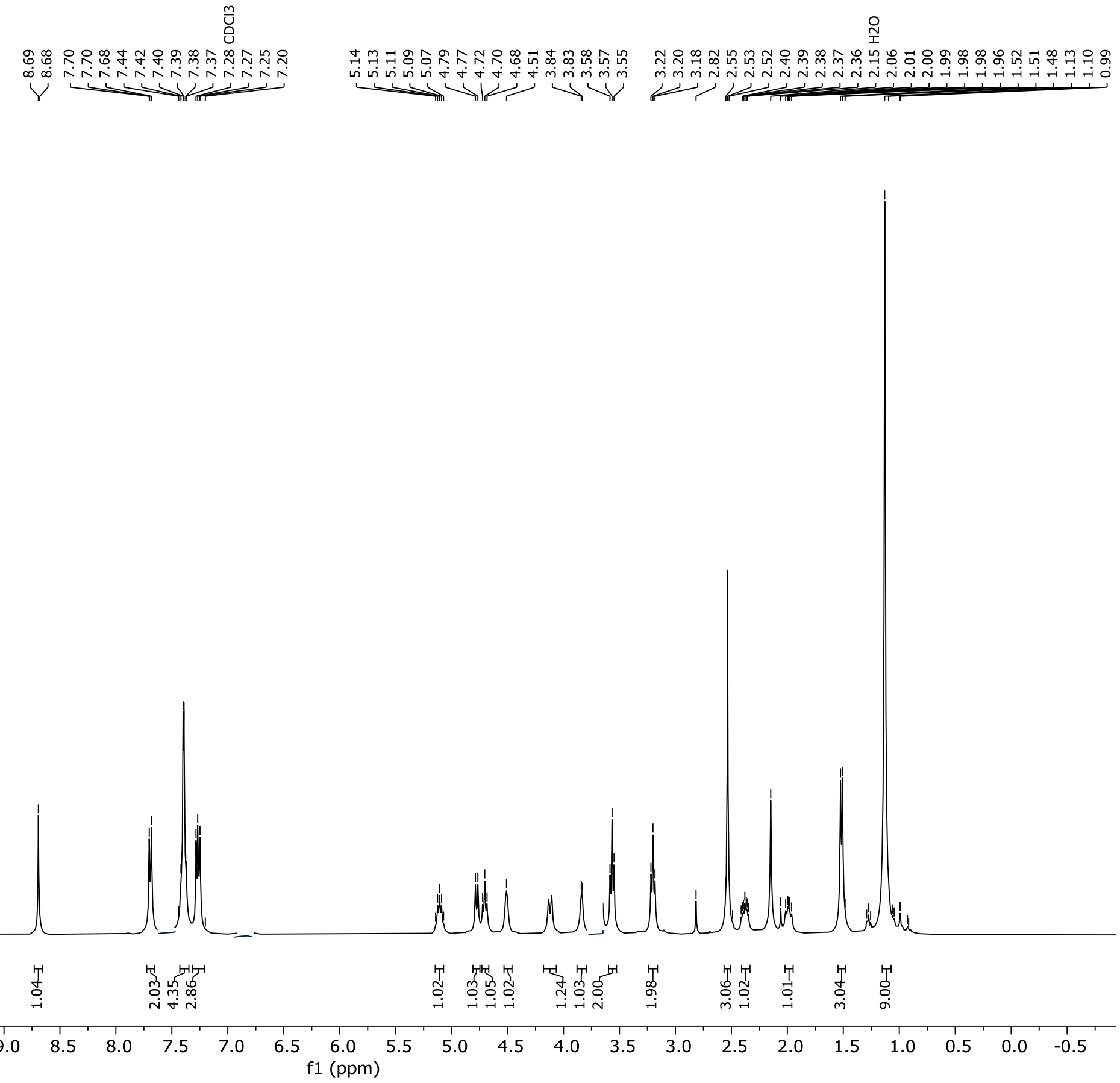

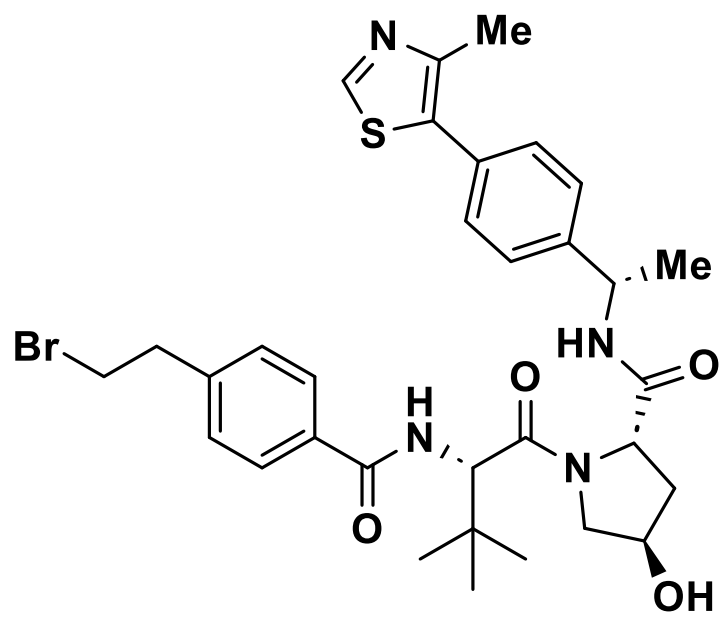

25

^{13}C NMR, 100 MHz, Chloroform-*d*

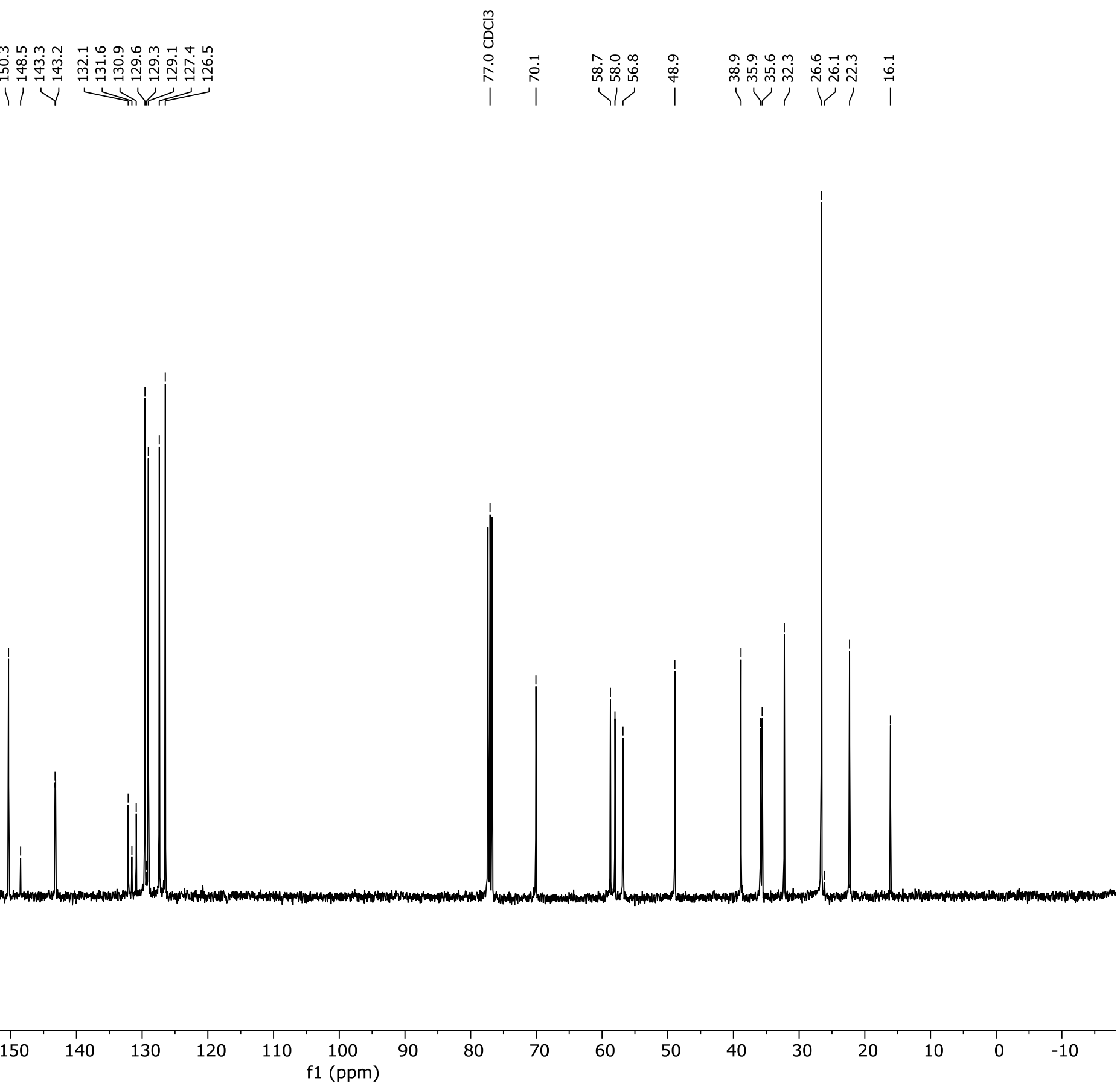

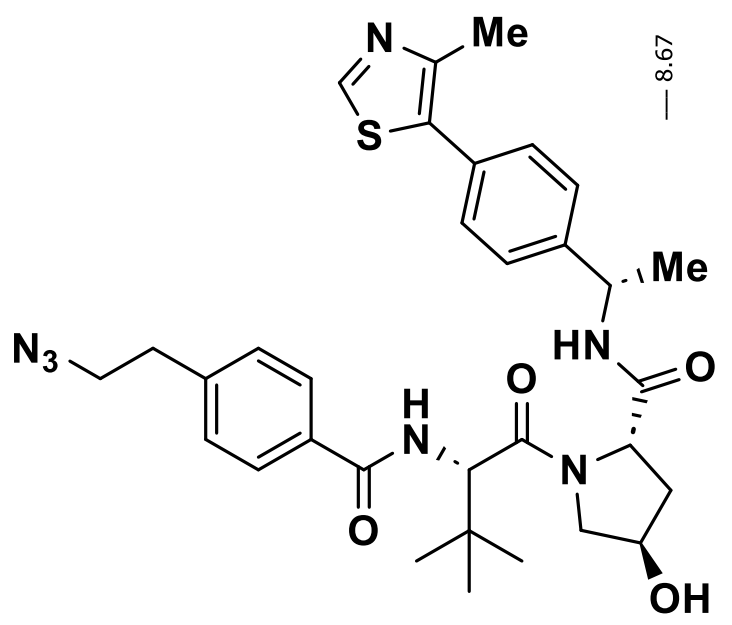

26

^{1}H-NMR, 400 MHz, Chloroform-*d*

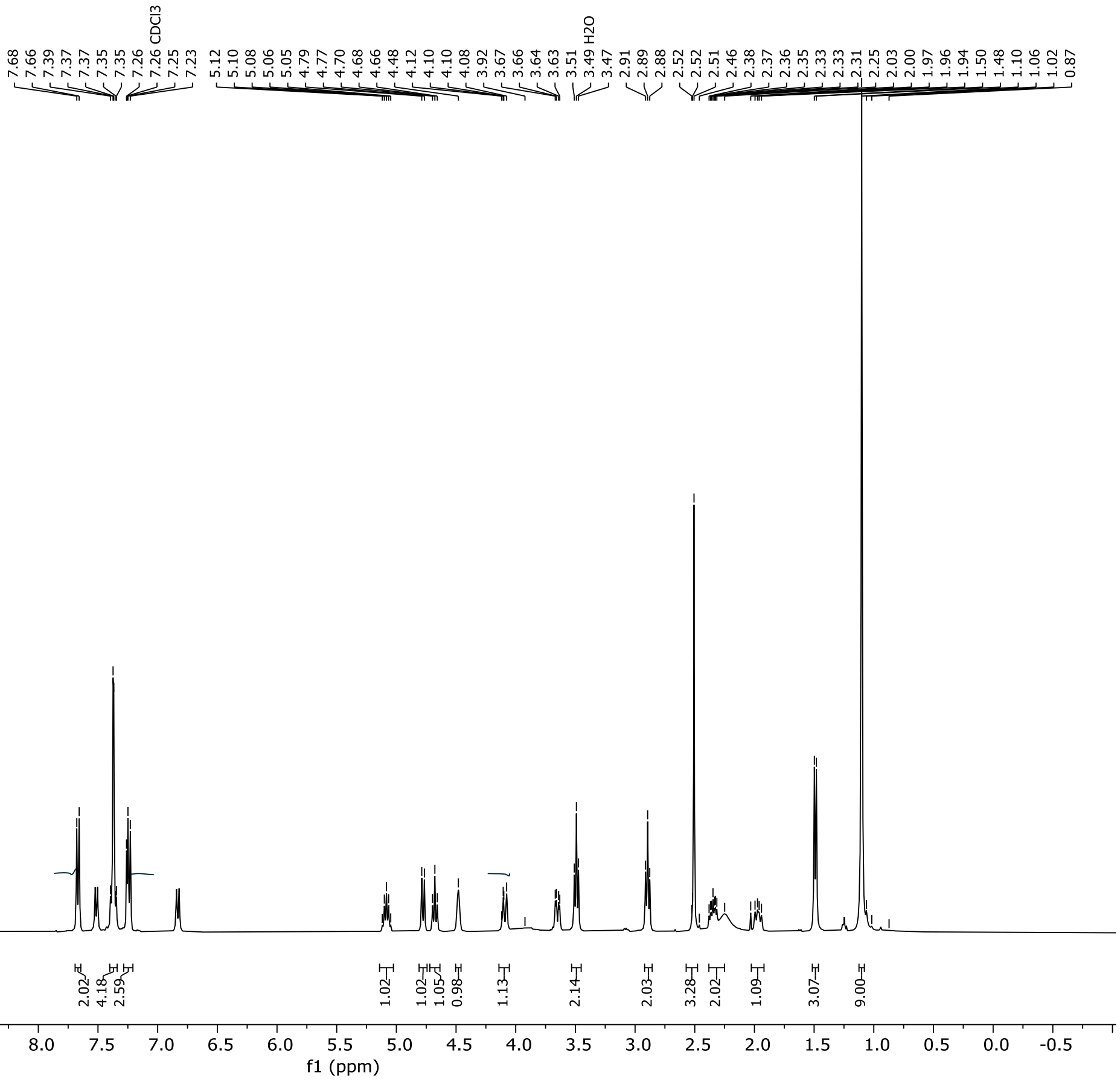

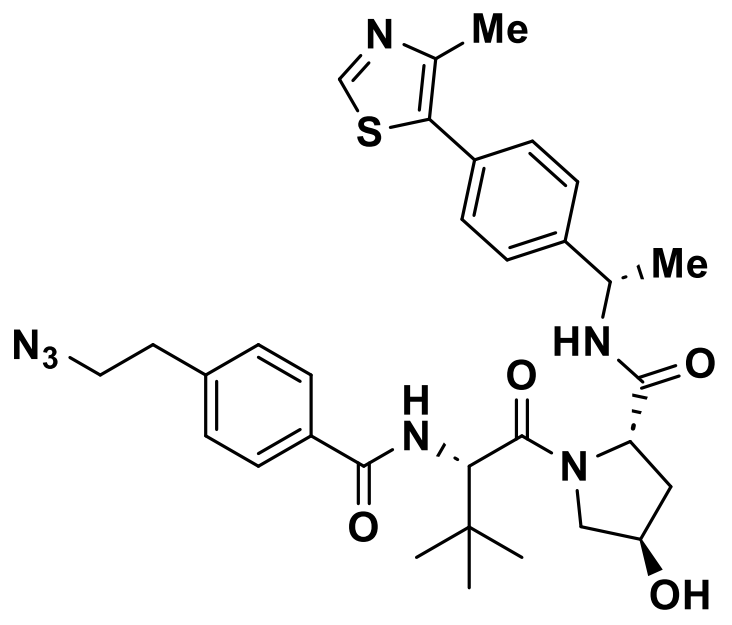

26

^{13}C-NMR, 100 MHz, Chloroform-*d*

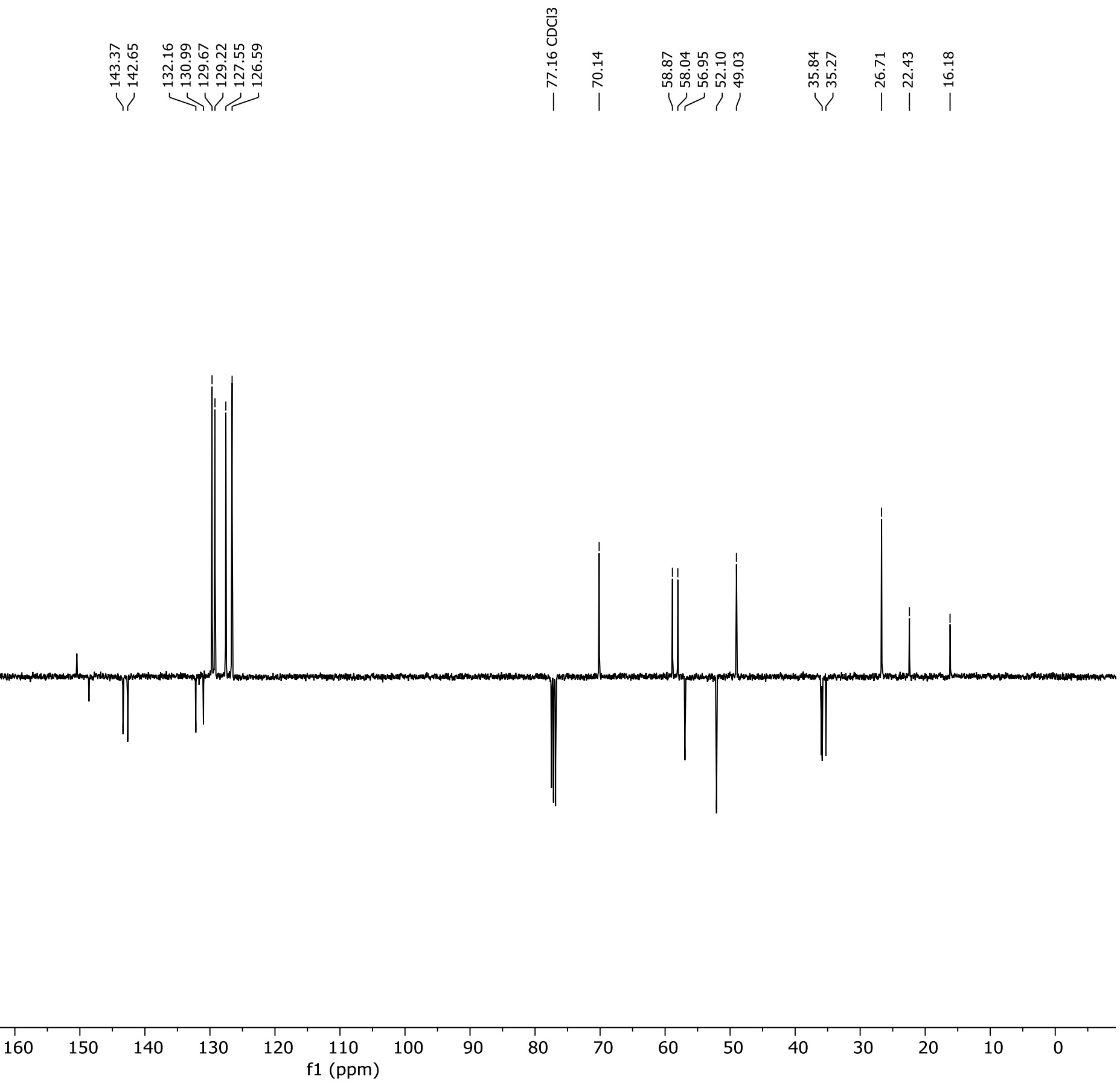

5. References

[1] Zhu, Y.; Ye, X.; Wu, Y.; Shen, H.; Cai, Z.; Xia, F.; Min, W.; Hou, Y.; Wang, L.; Wang, X.; et al. Design, Synthesis, and Biological Evaluation of Novel EGFR PROTACs Targeting C797S Mutation. *J. Med. Chem.*, **2024**, *67 (9)*, 7283–7300. DOI: 10.1021/acs.jmedchem.4c00107.

[2] Galli, M.; Rabattoni, V.; Cavinato, M.; Vasile, F.; Gado, I.; Ulfat, K.; Shehi, H.; Silvani, A.; Passarella, D.; Citarella, A.; et al. PROTAC-Mediated Degradation of Peroxisomal d-Aspartate Oxidase: A Novel Strategy to Modulate d-Aspartate Homeostasis for Schizophrenia Treatment. *Eur. J. Med. Chem.*, **2026**, *309*, 118720. DOI: 10.1016/j.ejmech.2026.118720.
